# Cold and hot tumors: from molecular mechanisms to targeted therapy

**DOI:** 10.1038/s41392-024-01979-x

**Published:** 2024-10-18

**Authors:** Bo Wu, Bo Zhang, Bowen Li, Haoqi Wu, Meixi Jiang

**Affiliations:** 1 Department of Neurology, The Fourth Affiliated Hospital, China Medical University, Shenyang, China; 2 Department of Youth League Committee, The Fourth Affiliated Hospital, China Medical University, Shenyang, China; 3 Department of Pancreatic and Gastrointestinal Surgery, Ningbo No. 2 Hospital, Ningbo, China; 4 Department of Gynaecology and Obstetrics, The Second Hospital of Dalian Medical University, Dalian, China

**Keywords:** Cancer microenvironment, Drug development

## Abstract

Immunotherapy has made significant strides in cancer treatment, particularly through immune checkpoint blockade (ICB), which has shown notable clinical benefits across various tumor types. Despite the transformative impact of ICB treatment in cancer therapy, only a minority of patients exhibit a positive response to it. In patients with solid tumors, those who respond well to ICB treatment typically demonstrate an active immune profile referred to as the “hot” (immune-inflamed) phenotype. On the other hand, non-responsive patients may exhibit a distinct “cold” (immune-desert) phenotype, differing from the features of “hot” tumors. Additionally, there is a more nuanced “excluded” immune phenotype, positioned between the “cold” and “hot” categories, known as the immune “excluded” type. Effective differentiation between “cold” and “hot” tumors, and understanding tumor intrinsic factors, immune characteristics, TME, and external factors are critical for predicting tumor response and treatment results. It is widely accepted that ICB therapy exerts a more profound effect on “hot” tumors, with limited efficacy against “cold” or “altered” tumors, necessitating combinations with other therapeutic modalities to enhance immune cell infiltration into tumor tissue and convert “cold” or “altered” tumors into “hot” ones. Therefore, aligning with the traits of “cold” and “hot” tumors, this review systematically delineates the respective immune characteristics, influencing factors, and extensively discusses varied treatment approaches and drug targets based on “cold” and “hot” tumors to assess clinical efficacy.

## Introduction

Immunotherapy aims to restore the innate antitumor immune response, revitalizing and maintaining the tumor-specific immune pathway.^1–5^ The development and clinical application of ICB therapies for various cancers have shown promising results.^6,7^ The efficacy of immunotherapy can vary among patients with the same cancer type, possibly due to the immune characteristics of the tumor.^8,9^ As a result, the concept of “cold-hot” tumor immune phenotypes has been proposed to characterize the immune status of tumors and predict the anticipated response to ICB therapy based on the type, quantity, and distribution of immune cell infiltration in the tumor microenvironment.^10^ This classification provides valuable insights for overcoming the shortcomings of initial treatment strategies.^11^

The complexity of the tumor microenvironment (TME) arises from the intricate interactions between tumor cells and various factors within the TME, involving both promoting and inhibitory mechanisms.^12^ Immune cells play a crucial role within the TME, with CD8^+^ T cells serving as pivotal participants in the immune response against tumors, effectively executing immune surveillance.^13^ Structurally, components such as tertiary lymphoid structures and the tumor stroma not only provide support for cellular activities but also play a significant role in shaping the TME.^14,15^ Moreover, the activities of tumor cells and immune cells are not independent but regulated by intrinsic factors such as epigenetic modifications and signaling pathways of the innate immune system.^16,17^ Both tumor cells and immune cells respond to external factors through metabolism, controlling tumor immune characteristics through metabolite-driven gene expression and interactions with the microenvironment.^18^ Also, various immune inhibitory components in cells and body fluids actively regulate the tumor immune microenvironment (TIME).^19^ Furthermore, microorganisms as hosts contribute to immune regulation and surveillance in the TME; their metabolites can reach distant tumor sites via the bloodstream, activating inherent immune responses against tumors or promoting tumor development.^20^ Overall, these factors collectively serve as key determinants influencing the diverse and intricate landscape of the TME.

The tumor microenvironment (TME) plays a significant role in determining the efficacy of immunotherapy in eliminating cancer cells. Extensive research has been devoted to incorporating immune factors into predictive models to evaluate the efficacy of individual or combined ICB therapies. Tumors can be categorized into three primary immune phenotypes based on the distribution and abundance of cytotoxic immune cells in the TME: immune-infiltrated, immune-excluded, and immune-deserted.^21,22^ Immune-inflamed tumors, also known as “hot” tumors, are characterized by high levels of T cell infiltration, increased PD-L1 expression, and elevated tumor mutational burden (TMB), making them more responsive to immune checkpoint inhibitors. Conversely, tumors transitioning towards immune-excluded and immune-deserted states are termed as “altered” and “cold” tumors, respectively. Immune-excluded tumors confine CD8^+^ T lymphocytes to the periphery, impeding their infiltration into the central tumor mass. On the other hand, immune-deserted tumors lack CD8^+^ T lymphocytes both within the tumor and its surroundings. These tumors often harbor immune-suppressive cell populations such as tumor-associated macrophages (TAMs), regulatory T (Treg) cells, and myeloid-derived suppressor cells (MDSCs). The characteristics of “altered” and “cold” tumors suggest a deficiency in intrinsic anti-cancer immune capabilities, resulting in limited responses to ICB.^6,7,23,24^

Our objective is to investigate the intricate interplay among cancer cells, immune cells, and a plethora of intrinsic and extrinsic elements, focusing on the “cold-hot” tumor immunophenotypes. This article thoroughly examines various potential immunotherapeutic strategies, including immune checkpoint inhibitors, T-cell immunotherapy, cancer vaccines, cytokines, and other tactics. By exploring current immune modulation techniques, we underscore the pivotal role of combination therapy and personalized approaches in problem-solving and improving treatment outcomes. This article endeavors to advance the field of cancer immunotherapy by scrutinizing the complex mechanisms of immunology and exploring strategies to convert immunologically inert “cold” tumors into active “hot” tumors, aiming to enhance clinical results and improve the quality of life for patients with tumors that have shown resistance to conventional therapies.

## “Hot and cold” tumors: immune escape mechanisms

**Fig. 1:**
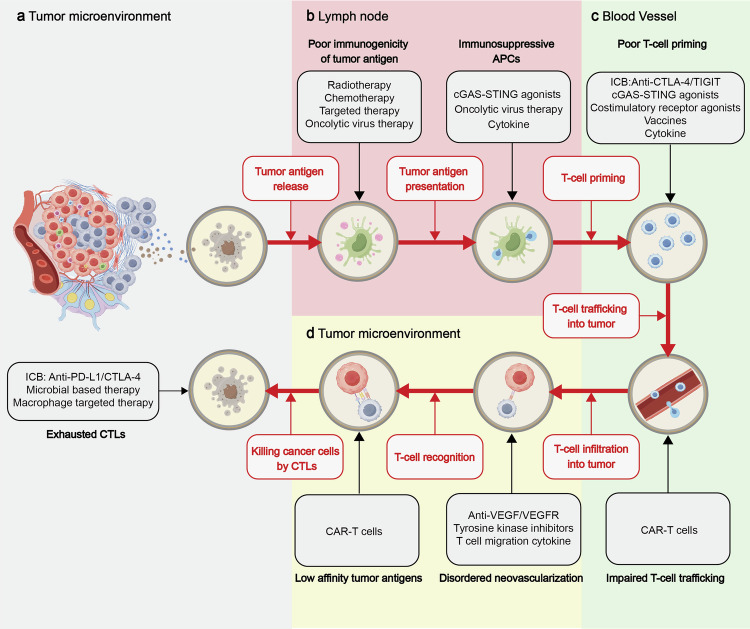
Illustrates the mechanisms underlying the anti-tumor immune response and immune evasion. The effectiveness of the anti-tumor immune response hinges on the activation, infiltration, and cytotoxic activity of effector T cells. These crucial processes encompass: **a** initiation of the T cell-mediated anti-tumor immune response through recognition of tumor-specific antigens (TSAs) in tumor microenvironment; (**b**) uptake and processing of tumor-specific antigens by dendritic cells (DCs); facilitation of cross-presentation in lymph node draining areas; (**c**) priming of naive T cells; recruitment of T cells by chemokines in blood vessels; (d) and identification and elimination of tumor cells in tumor microenvironment. Mechanisms of tumor immune evasion include characteristics that (a) diminish tumor immunogenicity, such as the absence of novel antigens, reduced expression of HLA molecules, or interference with antigen presentation to HLA molecules; **b** defects in antigen presentation possibly linked to dysfunctional DCs, affecting recruitment, activation, maturation, antigen cross-presentation, and T cell priming; **c** within the tumor microenvironment (TME), restrictions on T cell migration due to inadequate chemokine secretion and compromised chemotactic function of peripheral T cells are observed. Furthermore, abnormal vascular structures and a matrix rich in collagen/fibroblasts impede T cell infiltration. Genetic abnormalities in tumors also hinder T cell migration and infiltration; **d** Tumors and their immunosuppressive TME play a significant role in inducing T cell dysfunction and apoptosis. Immunotherapy is grounded in principles like tumor antigen release and presentation, T cell priming and activation, T cell migration and infiltration into tumors, and activation of T cell effector functions. Various therapeutic modalities, including chemotherapy, radiotherapy, targeted therapy, and anti-angiogenic therapy, aim to modulate the immune microenvironment and augment the efficacy of immunotherapy. In Fig. 1, the red dashed arrows symbolize effector T cells advancing anti-tumor responses, while the black dashed bars depict obstacles encountered by effector T cells during the anti-tumor response. This figure was created using Figdraw

The effectiveness of anti-tumor immune responses, within the framework of cell-mediated immunogenic cell death (ICD),^25^ hinges on the activation, mobilization, infiltration, viability, detection, and elimination of tumors by effector T cells. Any breakdown in the process of ICD can result in immune evasion (Fig. 1). This study delves into the mechanisms through which tumors evade immune detection by leveraging intrinsic adaptability and external support, culminating in the manifestation of an immune “cold” phenotype as evidenced in recent research findings.

### Defects in tumor cell antigen presentation

Tumor-specific antigens (TSAs) play a critical role in initiating T cell-mediated anti-tumor immune responses.^26,27^ TSAs primarily arise from non-synonymous mutations in tumor cells, collectively known as the tumor mutation burden (TMB).^28^ Beyond DNA coding sequence mutations, the generation of new antigens can result from gene fusion events, mutations in non-coding regions, alternative splicing, and deficient mismatch repair (dMMR), leading to microsatellite instability (MSI).^29,30^ Individuals with high TMB or dMMR/MSI tumors typically exhibit enhanced responses to immunotherapy with programmed death-1 (PD-1)/programmed cell death ligand 1 (PD-L1) inhibitors or cytotoxic T lymphocyte-associated protein 4 (CTLA-4) checkpoint blockade.^31–35^ Tumor-derived neoantigens are displayed on the tumor cell surface by human leukocyte antigen (HLA) molecules for interaction with T cells. Altered expression of the major histocompatibility complex class I (MHC-I) molecules on tumor cells can occur via genetic, epigenetic, and transcriptional mechanisms.^36–40^ Loss of heterozygosity in HLA-I genes contributes to immune evasion against tumor neoantigens in approximately 17% of pan-cancer analyses.^36^ Epigenetically, the polycomb repressive complex 2 (PRC2) methylates lysine 27 on histone H3 (H3K27) to repress the transcription of MHC-I antigens, aiding in immune evasion.^41,42^ Various genes, including *B2m, H2-K1, Tap2, Nlrc5, Tapbp* as positive regulators, and *Ezh2, Med13, Tada3, Traf3* as negative regulators, control MHC-I transcription at the level of gene regulation.^38^ Experimental evidence has shown that *Traf3*-deficient melanoma cells exhibit increased MHC-I expression, enhancing T cell-mediated killing.^38^ Autophagy-mediated degradation of MHC-I on tumor cells can be targeted for therapeutic intervention to restore MHC-I presentation and increase cytotoxic T lymphocyte (CTL) presence.^39^ Combined use of autophagy inhibitors with PD-1 or CTLA-4 immune checkpoint blockade enhances CTL presence in pancreatic ductal adenocarcinoma (PDAC) and non-small cell lung cancer (NSCLC) murine models, suggesting a potential treatment strategy for HLA-I-deficient tumors.^39,43^ Human leukocyte antigen-G (HLA-G), abundantly expressed on various cancer cells, plays a crucial role in immune evasion and immunosuppressive cell proliferation. The release of HLA-G from cell membranes can modulate the tumor microenvironment, highlighting its potential as a next-generation immune checkpoint for cancer therapy.^44^ This discovery illuminates the strategies employed by tumors to evade immune surveillance and underscores the value of T cell-focused immunotherapies for HLA-I-deficient tumors.

### Dendritic cell antigen presentation deficits

Dendritic cells (DCs) play a crucial role in initiating immune responses against tumors by capturing and processing tumor-specific antigens, facilitating cross-presentation, and activating naïve T cells. The recruitment of DCs is mediated through C-C chemokine ligand 5 (CCL5) and X-C motif chemokine receptor 1 (XCL1), with their activation triggered by various danger signals, such as cytosolic DNA, RNA, ATP, calreticulin (CRT), and high mobility group box 1 (HMGB1).^45,46^ For instance, DCs recognize intracellular tumor-derived DNA through the cyclic GMP-AMP synthase (cGAS) receptor, which activates the stimulator of interferon genes (STING) pathway, leading to the production of type I interferon (IFN-I) and enhancing DCs maturation.^47^ However, the maturation of DCs within tumors is often limited and hindered by various mechanisms, including T-cell immunoglobulin and mucin domain 3 (TIM3)-mediated inhibition of extracellular DNA uptake, disruption of cGAS-STING pathways, and the inhibitory effects of sialic acid-binding immunoglobulin-type lectins (SIGLEC) through intracellular immune receptor tyrosine phosphorylation sites.^48^ Tumor cells can also avoid DC phagocytosis by upregulating the “don’t eat me” signal CD47 or expressing proteins like stanniocalcin 1 (STC1) and glycosylated B7-H4 to mask calreticulin, which is essential for the exposure of the “eat me” signal.^49,50^ Additionally, the secretion of gelsolin (sGSN) by tumor cells can impede the recognition and cross-presentation of tumor antigens by conventional type 1 dendritic cells (cDC1s) through the DNGR-1 receptor, hindering the stimulation of anti-tumor CD8^+^ T cells.^51^ Studies suggest that DCs can process novel antigens via the N^6^-methyladenosine (m^6^A)-binding protein YTHDF1.^52^ Inhibition of YTHDF1 in DCs has shown potential therapeutic benefits in enhancing tumor-specific CD8^+^ T cells, as evidenced in melanoma-bearing *Ythdf1*^−/−^ mice and colorectal cancer patients with low YTHDF1 expression, highlighting a promising approach for immunotherapy.^52^

In a murine model of pancreatic ductal adenocarcinoma (PDAC), conventional type 1 dendritic cells (cDC1s) within the tumor microenvironment (TME) exhibit reduced numbers, increased apoptosis, and impaired maturation. These attributes result in compromised antigen presentation, consequently influencing the activation of CD8^+^ T cells.^53^ Notably, the incomplete maturation of DCs leads to a deficiency in the generation of costimulatory molecules, accompanied by an upregulation of coinhibitory receptors aiming to suppress T cell activation.^54^ Moving to lung cancer, mature DCs display a significant presence of PD-L1, which negatively impacts co-stimulation by hindering the interaction between CD80 on DCs and CD28 on T cells.^54^ In a murine model of hepatocellular carcinoma (HCC) characterized by high immunogenicity and driven by MYC, but with a deficiency in *Trp53*, the overexpression of β-catenin within tumor cells obstructs the recruitment of DCs, leading to immune evasion and resistance to ICB therapy targeting the PD-1 antibody.^55^

Dendritic cells (DCs) found in tumors expressing chemokine receptor 7 (CCR7) demonstrate lower immunogenicity compared to DCs in non-malignant tissues.^56^ This reduced immunogenicity is marked by decreased interleukin 12 (IL-12) production, heightened levels of PD-L1 expression at both mRNA and protein levels, and upregulated CMTM6 expression—a critical regulatory factor responsible for stabilizing PD-L1.^57^ Studies suggest that the efficacy of PD-L1 ICB therapy is influenced by mature dendritic cells expressing PD-L1 and CD8^+^ T cells expressing PD-1, primarily located in tumor-draining lymph nodes (TDLNs).^58^ Evaluation in murine models of mesothelioma and colorectal cancer demonstrates that local administration of a small dose of PD-L1 monoclonal antibody effectively disrupts the PD-L1/PD-1 pathway in regional TDLNs, resulting in significant tumor regression and enhanced survival rates.^58^ These findings indicate potential functional deficiencies in DCs concerning recruitment, activation, maturation, antigen presentation, and immune initiation, potentially elucidating the phenomenon of high TMB but low T cell inflammation in specific cancer patients. Further investigations are required to unravel the precise underlying mechanisms.

### Impediments to T cell trafficking and infiltration

Following initial activation, chemokines and their corresponding receptors in draining lymph nodes recruit T cells. The process of T cell infiltration and persistence in the tumor microenvironment is complex and tightly regulated due to barriers posed by blood vessels and tumor stroma.^59,60^ Precise control is necessary to coordinate the behavior of immune cells as they travel to the tumor site.^61^ Chemokines such as C-X-C motif chemokine ligands 9 (CXCL9) and C-X-C motif chemokine ligands 10 (CXCL10) recruit CD8^+^ T cells, natural killer (NK) cells, and helper T cell 1 (Th1) cells by binding to the C-X-C motif chemokine receptor 3 (CXCR3).^60^ Notably, the secretion of CCL5 by NK cells is crucial for recruiting cDC1s, leading to the production of CXCL9 and CXCL10, which promote T cell migration.^45^ Tumor-derived CCL5 enhances the migration of CD8^+^ T cells, while DC-produced CXCL9 facilitates the infiltration of tumor-infiltrating lymphocytes (TILs).^62^ TAMs recruit naive CD4^+^ T cells through CCL18, driving their differentiation into Treg cells.^63^ However, cancerous tumors often exploit chemokines to attract immunosuppressive T cells.^64^ For instance, colorectal cancer cells upregulate the secretion of chemokines like CCL17, CCL22, and CXCL12 to attract regulatory T cells, as well as CXCL1, CXCL2, and CXCL3 to recruit MDSCs.^64^ Some chemokines, like CCL5, simultaneously recruit both anti-tumoral and immunosuppressive T cell subsets.^65^ The complexity of cell migration responses to chemokine networks is influenced by various factors, such as cell diversity, tumor cell types and quantities, and interactions among different chemokines within the tumor microenvironment. It is important to note that while chemokines are pivotal in the tumor microenvironment, they do not solely dictate T cell recruitment. Research has demonstrated that the protein GTPase activator regulator of G protein 1 (RGS1) in tumor-specific circulating T cells acts as a suppressor of chemokine G protein-coupled receptors (GPCRs) signaling, leading to reduced T cell motility and decreased levels of infiltration by CTL and Th1 cells in mouse models, breast cancer, and lung cancer patients,^66^ underscoring the significance of regulating chemokine receptor function.

Tumor blood vessels display characteristics such as incomplete vascular development and increased leakiness, accompanied by stromal elements including fibroblasts and extracellular matrix (ECM). These factors collectively create barriers that impede the infiltration of T cells into the tumor.^67,68^ Studies have shown that CD8^+^ T cells are excluded from metastatic urothelial carcinoma (mUC) patients with abundant fibroblasts and collagen.^69^ Additionally, inadequate responses to ICB have been observed in patients with mUC and colorectal cancer (CRC), possibly due to the activation of transforming growth factor β (TGF-β).^69,70^ In a mouse model simulating triple-negative breast cancer (TNBC), researchers have identified the involvement of discoidin domain receptor family member 1 (DDR1), a collagen receptor, in promoting the rearrangement of collagen fibers within the ECM. This restructuring of the tumor microenvironment hinders the migration of CD4^+^ and CD8^+^ T cells towards the central tumor zone.^68^ Studies have shown that inhibiting TGF-β with neutralizing antibodies or genetic deletion of DDR1 can enhance T cell infiltration in murine models of mUC and TNBC, effectively overcoming immune exclusion within the central tumor area.^68^ Understanding these mechanisms sheds light on immune exclusion, providing avenues for identifying new immune therapeutic targets to enhance immune cell infiltration. Moreover, studies in a mouse model of PDAC have demonstrated the crucial role of CXCL1 released by tumor cells in diminishing the CD8^+^ T cell population. Abolishing CXCL1 in tumor cells could improve the migration of CD8^+^ T lymphocytes, thereby boosting the effectiveness of ICB and effectively managing tumor progression.^71^ Various intrinsic factors in tumors, such as PTEN loss combined with PI3K pathway activation in tumors, can obstruct T cell infiltration.^72,73^ Overall, the components within the tumor microenvironment (TME) regulate the movement and infiltration of anti-tumor T cells through intricate processes, leading to immune evasion and the development of “cold” tumors. These pivotal molecules present promising prospects for enhancing the efficacy of ICB therapies.

## “Hot” tumors: tumor immune response

In contemporary tumor immune characterization, the presence of immune-infiltrating cells is crucial. CD8^+^ T and NK cells represent acquired and innate immunity against tumors, collaborating in immune surveillance. Studies show that enhancing NK cell activity to impede tumor growth can be achieved through the use of PD-1/PD-L1 inhibitors, offering a promising strategy for ICB therapy.^74,75^ Given the intricate mechanisms of tumor evasion from the immune system and the considerable heterogeneity across tumor types, it is advisable that immune profiling techniques encompass not only cytotoxic CD8^+^ T cells but also NK cells.

### CD8^+^ T cells mediate “hot” tumors

CD8^+^ T cells play a crucial role in mediating the immune response within “hot” tumors. The effectiveness of immune checkpoint blockade (ICB) therapy stems from the rejuvenation of CD8^+^ T cells, which detect tumor antigens presented by MHC-I molecules on tumor cells through a peptide-specific mechanism.^76^ Interferon (IFN) can enhance antigen presentation and MHC expression.^77^ Upon recognizing antigens, CD8^+^ T cells secrete perforins, granzymes, and IFN-γ, leading to the death of tumor cells.^78^ The significant infiltration of CD8^+^ T lymphocytes serves as an indicator of an immune response within “hot” tumors. Nonetheless, the definition of “hot” tumors remains vague, often merely characterized by the proximity of T cells to tumor cells.^79–81^ Recent advanced studies utilizing single-cell sequencing and spatial transcriptomics have unveiled substantial diversity in the composition and features of infiltrating CD8^+^ T cells in the tumor microenvironment (TME).^82,83^

#### Differentiation and functional dysfunction of tumor CD8^+^ T cells

Tumor growth can persist despite the presence of CD8^+^ T cells that target tumors specifically, mainly due to the overall dysfunction and exhaustion of T cells. This condition is marked by increased expressions of suppressive immune checkpoint proteins such as PD-1, TIM-3, TIGIT, and LAG-3.^6,84–86^ Tumor-specific CD8^+^ T cells may remain in a quiescent and immature state at the onset of tumor development, anticipating a significant number of tumor cells and antigens for activation.^87^ The lack of assistance from CD4^+^ T cells impedes antigen-presenting cells from effectively displaying tumor antigens, thus hindering the activation of innate immune responses prompted by pathogen-associated molecular patterns and resulting in a state of tolerance characterized by diminished responsiveness akin to “starvation”.^88^ Research has revealed a correlation between a higher CD8^+^/Treg cells or Th1/Th2 ratio and more robust anti-tumor immune responses, reduced tumor cell proliferation, and enhanced overall survival.^89,90^ The activation of CD8^+^ T cells requires the simultaneous stimulation of the T cell receptor (TCR) and CD28, initiating various signaling pathways like MAPK, JNK, PI3K/AKT, and IKK. Subsequent activation of transcription factors, including nuclear factor of activated T cells (NFAT).^91^ Calcineurin dephosphorylates cytoplasmic NFAT, enabling its translocation to the nucleus, where it collaborates with activating protein-1 (AP-1) to initiate transcription associated with effector function genes such as IL2 and IFNG. In the absence of co-stimulatory signals, TCR interaction inhibits the activation of MAPK, PI3K/AKT, and IKK signaling pathways, thereby reducing AP-1 functionality. Once activated, NFAT complexes cannot synergistically amplify transcriptional programs linked to the effector functions of CD8^+^ T cells along with molecules like AP-1. Instead, they interact with inhibitory transcription factors (such as EGR2, EGR3, IKZF2, IRF4, and TOX) and additional negative regulators (like CBL-B), thereby fostering T cell dysfunction.^92^

As tumors progress, T cells undergo stimulation by antigens, leading to a state of late-stage functional exhaustion, marking the second stage of T cell differentiation. Revitalizing exhausted CD8^+^ T (Tex) cells to regain functionality poses a significant therapeutic challenge, given the regulation of their epigenetic program by the transcription factor Tox.^93^ Tex^prog^ cells, also identified as stem-like CD8^+^ T cells, exhibit distinct characteristics such as the presence of TCF1 and the absence of TIM3 and PD-1 markers.^94,95^ These cells are noted for their longevity, robust proliferative capacity, adaptable differentiation potential, and heightened sensitivity to ICB. As Tex cells transition from progenitor (Tex^prog^) to intermediate (Tex^int^) and terminal (Tex^term^) subsets, there is a restructuring of their epigenetics, emphasizing their developmental plasticity.^96^ STAT5 plays a crucial role in the formation of Tex^int^ cells. Prolonged activation of STAT5a can propel exhausted CD8^+^ T cells towards a durable effector state, enhancing their capacity to combat tumors.^96^ Cells exhibiting a TCF1^-^TIM3^+^PD-1^+^ phenotype, categorized as Tex^term^ cells, manifest increased expression of various immune checkpoint molecules, including LAG3 and TIGIT, rendering them unresponsive to ICB.^97,98^ In a B16-OVA tumor mouse model, the transplantation of Tex^prog^ cells results in more sustained tumor control compared to Tex^term^ cells.^99^ The presence of TCF1^+^CD8^+^ Tex^prog^ cells in melanoma patients potentially signifies favorable outcomes and prolonged responses to PD-1 blockade.^99^ TCF1 acts as a transcription factor maintaining the undifferentiated state of Tex^prog^ cells, while TOX drives their differentiation into Tex^term^ cells, underscoring the roles of transcriptional and epigenetic regulation in governing T cell differentiation and offering avenues for enhancing cancer immunotherapy.^100^ Studies indicate that Id2 is implicated in the regulation of the differentiation process from effector progenitor (Tex^prog^) cells to terminal effector (Tex^term^) cells through transcriptional and epigenetic mechanisms. Deletion of Id2 suppresses CD8^+^ T cell-mediated immune responses and the persistence of stem-like CD8^+^ T cell subsets, consequently compromising the effectiveness of PD-1 blockade and increasing susceptibility to tumor development.^101^ The maturation of CD8^+^ T cells is modulated by various transcriptional regulators, including NFAT, NR4A, and TOX.^102^ Conversely, in a melanoma mouse model, transcription factors BATF and IRF4 impede T cell differentiation.^103^ Essentially, the goal of ICB therapy is to alleviate immune exhaustion and T cell decline in anti-cancer efforts. However, not all depleted T cells exhibit responsiveness to ICB treatment. Exploring additional regulatory pathways could provide insights into the factors contributing to specific T cell resistance to ICB, thereby unveiling new opportunities for immunotherapeutic interventions.

Diverse T cell immune phenotypes coexist within the tumor microenvironment (TME), collectively exerting a significant influence on shaping adaptive immune responses against tumors. In the context of cutaneous squamous cell carcinoma, it has been observed that CD8^+^ T cells and Treg cells are closely located in the tumor stroma, while CD8^+^ T cells are notably scarce in the central zone of the tumor. This observation implies the potential recruitment of Treg cells in conjunction with CD8^+^ T cells, a process that may disrupt the communication between CD8^+^ T cells and tumor cells.^104^ Additionally, a study revealed that in tumors, activated CXCR3^+^ Treg cells interact with BATF3^+^ DCs, leading to the production of CXCL9. The deletion of CXCR3 in Treg cells disrupts their interaction with DC-Treg cells but enhances their interaction with DC-CD8^+^ T cells.^105^ Through mass spectrometry analysis and single-cell TCR sequencing in human melanoma, CRC, and lung cancer patients, it was discovered that certain CD8^+^ T cells respond to non-tumor-related antigens, termed bystander T cells.^106,107^ Despite infiltrating tumors, these memory-like CD39-CD8^+^ T cells exhibit no signs of chronic antigen stimulation, thereby reducing their response to ICB.^106,107^ Although bystander T cells in NSCLC and melanoma patients sometimes manifest cross-reactivity with tumor antigens,^108,109^ their exact role in anti-tumor immune responses remains largely unknown. Consequently, merely classifying tumors as “hot” based on CD8^+^ T cell infiltration is inadequate, highlighting the necessity for a more precise characterization of CD8^+^ T cell subpopulations within the TME.

#### Differential death of CD8^+^ T cells in tumor microenvironment

The significance of T cell apoptosis in shaping the strength and duration of immune responses cannot be overstated; however, their specific role in immune evasion within the tumor microenvironment is often disregarded.^110,111^ Apoptosis activation in CD8^+^ T cells is triggered by TCR activation and external signals, such as receptors like FAS.^111^ Research indicates that conditional deletion of KRAS^G12D^ in a PDAC genetic mouse model can reactivate FAS, induce CD8^+^ T cell-driven apoptosis, and completely eliminate tumors.^112,113^ In the tumor setting, tumor endothelial cells selectively eradicate CD8^+^ T cells through the FasL-Fas interaction without harming Treg cells.^114^ Furthermore, elevated NF-κB levels suppress the expression of the long non-coding RNA NKILA, increasing the susceptibility of activated cytotoxic T cells (CTLs) and Th1 cells to activation-induced cell death (AICD) compared to T cell subsets known for tumor suppression.^115^ Targeted interventions against NKILA are anticipated to safeguard effector T cells from apoptosis, potentially heralding new advances in T cell therapy. Another form of T cell demise is ferroptosis, a pathway reliant on iron accumulation that triggers the generation of reactive oxygen species (ROS) and brings about cell death by lipid peroxidation of the cell membrane.^116^ Extending the longevity of CD8^+^ T cells is achievable by administering the ferroptosis inhibitor ferrostatin-1.^117^ CD36 enhances the ferroptosis process in CD8^+^ T cells by promoting lipid and fatty acid uptake from the tumor microenvironment (TME), leading to amplified lipid peroxidation and eventual cell death.^116,117^

Recent investigations have demonstrated that Interferon-gamma (IFNγ) promotes the expression of ACSL4, leading to alterations in lipid profiles within tumor cells, consequently increasing the presence of arachidonic acid (AA) in phospholipids containing C16 and C18 acyl chains. This process enhances ACSL4-mediated tumor ferroptosis triggered by the combined action of IFNγ and AA.^118^ In contrast, in mice with YUMM1.7 melanoma, the survival of Treg cells within the tumor is also reliant on CD36, implying notable variations in the regulation of T cell death among distinct subgroups.^119^ Overall, different T cell subsets exhibit varied responses to apoptotic signals, influencing the infiltration of CD8^+^ T cells, which can impact the progression of “cold” tumors. The identification of tumors highly responsive to ICB necessitates critical assessments, including the evaluation of CD8^+^ T cell infiltration level, the specific CD8^+^ T cell subsets found within the tumor, their interaction with the tumor, and their functional status. This evaluation is indispensable for pinpointing tumors that are genuinely “hot” and more likely to demonstrate enhanced outcomes following cancer immunotherapy.

### NK cells mediate anti-tumor immunity in “hot” tumors

In various types of cancer, NK cells, known for their cytotoxic abilities against tumor cells, have been associated with improved overall survival rates. The significance of NK cells is underscored by the limited T cell responses imposed by HLA-I in most tumors that lack HLA-I expression.^94,120^ Studies have shown that the presence of NK cells expressing both PD-1 and PD-L1 plays a crucial role in determining the efficacy of PD-1/PD-L1 blockade therapy in mouse tumor models.^74^ The efficacy of PD-L1 blockade in suppressing tumor growth and impeding tumor progression substantially diminishes with a reduction in NK cell levels.^74^ Moreover, cancer patients exhibit shared inhibitory receptors, such as TIGIT,^121^ NKG2A,^122^ KLRB1,^123^ and IL18BP,^124^ on both NK cells and T cells, suggesting the potential for a dual targeting approach in the context of ICB therapy. Flow cytometric analysis in patients with renal cell carcinoma (RCC) has revealed two distinct subgroups: one group characterized by increased CD3 expression and the other group demonstrating elevated levels of NK cells.^125^

The NK-high subgroup showed a significant association with reduced intratumoral T cell infiltration in patients.^125^ These findings suggest that tumors characterized by high NK cell infiltration may potentially exhibit a favorable response to immune checkpoint inhibition, regardless of T cell infiltration levels and HLA-I expression. In addition to their direct cytotoxic effects, NK cells also modulate adaptive immune responses by producing IFN-γ, which notably promotes DCs maturation.^126^ In melanoma, NK cell-derived CCL5 and XCL1 play a crucial role in recruiting cDC1s. Dendritic cells play a key role in presenting tumor-specific antigens effectively and activating CD8^+^ T cells, underscoring the significance of NK cells in enhancing T cell-driven immune responses against tumors. This underscores the substantial importance of NK cells in boosting T cell-driven anti-tumor immune responses in the context of melanoma.^45^ NK cells and CD8^+^ T cells are vital effector cells in the anti-tumor immune response, serving as crucial targets for immune checkpoint molecules. They contribute to two distinct forms of anti-tumor immunity: adaptive immunity mediated by T cells targeting HLA-I-expressing tumors and innate immunity mediated by NK cells targeting HLA-I-deficient tumors. Furthermore, targeting NK cells has demonstrated efficacy against tumors lacking HLA-I expression. Therefore, exploring the potential of NK cell therapy and inhibiting NK cell immune checkpoints show promise and warrant further investigation.

## The cGAS-STING pathway: innate immunity in tumor “cold-to-hot”

The crucial role of the tumor microenvironment (TME) in tumor initiation, growth, and inhibition has been highlighted.^127^ This importance is manifested in the control exerted over both tumor cells and immune cells through innate immune cell signaling cascades. Research has emphasized the complex interactions taking place within the TME and the influence they have on tumorigenesis and immune responses.^128^ Therefore, tumor monitoring heavily relies on the innate immune system’s ability to detect, control, and eliminate malignant cells.^129^ The innate immune system functions through pattern recognition receptors, which play a vital role in detecting and responding to tumor cells even in the absence of specific molecular markers indicating cancer presence. Conversely, the accumulation of damaged associated molecular patterns can actively trigger these receptors.^130^ The cGAS-STING pathway, which is essential for recognizing abnormal DNA, shows potential for application in anti-cancer immunotherapies.^17,130^

### Activation and regulation of the cGAS-STING pathway

The cyclic GMP-AMP synthase (cGAS) recognizes double-stranded DNA in a sequence-independent manner, leading to the activation and production of cyclic GMP-AMP (cGAMP).^131^ Subsequently, cGAMP binds to and activates the Stimulator of Interferon Genes (STING). This activation initiates the transcription of Interferon Regulatory Factor 3 (IRF3) and NF-κB, culminating in the generation of inflammatory cytokines and chemokines.^130^ The cGAS-STING signaling pathway also plays a critical role in enhancing endogenous antigen presentation by upregulating the levels of co-stimulatory molecules.^132,133^ Prolonged STING pathway activation is associated with the modulation of gene transcription in the immune system.^134^ Apart from inducing transcriptional responses, STING activation can stimulate processes like autophagy and cell death, aiding in the clearance of pathogens or their derivatives during infections.^135,136^

Various mechanisms have evolved to prevent the unintended activation of the internal immune system by limiting self-DNA recognition and termination of downstream signal transduction. These mechanisms include cellular and extracellular clearance processes for self-DNA, such as three prime repair exonuclease 1 (TREX1), lysosomal DNase II, and adenosine deaminase 2 (ADA2) to prevent cGAS-dependent autoimmunity.^137,138^ Interactions between nucleosomes and chromatin structure proteins also play a role in evading intact genomic DNA sensing.^139,140^ Transport restrictions by ABCC1 and other transport proteins limit intracellular STING-dependent activation at the level of cGAMP.^141^ Extracellularly released cGAMP is enzymatically degraded by the membrane-bound extracellular nucleotide phosphodiesterase ENPP1.^142^ The intracellular transport of STING regulates its crucial role in activation and regulation, as it moves from the endoplasmic reticulum to the Golgi apparatus for efficient degradation in lysosomes.^143^ Cells utilize various intracellular and extracellular protective mechanisms to maintain a balance and promptly resolve immune responses triggered by the cGAS-STING pathway.

### Mechanisms of involvement of cGAS-STING pathway in tumors

The report highlights the activation of cyclic GMP-AMP synthase (cGAS) during malignant transformation and treatment processes by DNA originating from various sources. Defects in DNA damage recognition, signaling, or repair, including the DNA damage response (DDR), are considered hallmarks of cancer.^144^ Numerous studies emphasize the relationship between intrinsic DDR defects and the immuno-stimulatory properties of tumor cells activated through the cGAS-stimulator of interferon genes (STING) pathway.^144^ The interaction of DNA damage with cellular cGAS primarily relies on two key mechanisms: the excessive production of abnormal DNA fragments in the nucleus and the formation of micronuclei (MN) or age-related chromatin fragments in the cytoplasm are plausible events.^131,145,146^ Extensive DNA damage caused by intrinsic carcinogenic processes or exposure to mutagens such as radiation or chemotherapy leads to the generation of atypical double-stranded DNA segments involved in DNA repair.^131,147^ Cancer cells dependent on the cGAS-STING pathway display prominent characteristics of type I interferon in these segments. In this context, the distinct structure of extracellular DNA generated in tumor cells can elicit a strong type I interferon response, as it relies on the cGAS-STING pathway.^148^ Considering the sustained presence of cGAS in the nucleus, the induction of nuclear DNA may also partly promote cGAS activation, although cytoplasmic sources are predominantly acknowledged.^149^

Unrepaired genomic damage or chromosome segregation defects may lead to nuclear and cytoplasmic abnormalities, giving rise to so-called micronuclei.^147^ Additionally, unresolved DNA breaks and chromosome fragments during mitotic arrest provide additional chromosome substrates for cGAS activation and can be observed in pre-cancerous senescent cells or tumor cells.^147^ Micronuclei or chromosomal fragmentation expose chromatin to the cytoplasm, causing DNA damage and chromosome breakage, providing highly immune-stimulatory double-stranded DNA segments for cGAS binding and activation.^150^ Increased DNA damage is attributed to the loss of nuclear membrane integrity, cleavage of chromosomal bridging DNA mediated by three prime repair exonuclease 1 (TREX1), and DNA processing mediated by apurinic/apyrimidinic endonuclease 1 (APE1) in the absence of excision repair cross-complementation group 1 (ERCC1) or breast cancer type 1 and 2 (BRCA1/2).^137,141^ Studies suggest that DNA recognition within micronuclei or chromosomes explains the phenomenon of autonomous cGAS activation in various cancers.^146,151^

DNA damage induced by radiation exposure can lead to micronuclei formation and trigger cGAS-mediated innate immune response in tumor cells in various environments,^152^ including those with ERCC1 or BRCA1/2 deficiencies. PARP inhibitors exploit the cGAS-STING signaling pathway to boost the immunogenicity of tumor cells.^153^ Further investigation into micronuclei sheds light on their immunogenic potential. Upon nuclear membrane degradation, regulatory factors are activated, capable of either inhibiting or stimulating cGAS activity. Specifically, the nuclear exonuclease TREX1 degrades DNA within micronuclei, thereby suppressing type I interferon responses.^154^ Conversely, abnormal ESCRT-III mechanisms increase damaged micronuclei levels, stimulating the expression of pro-inflammatory genes linked to heightened micronuclear membrane permeability.^155^ Additionally, the inherent properties of chromatin can influence the recruitment and activation of cGAS, as different types of induced micronuclei exhibit notable variances in attracting cGAS.^155^

Besides aberrant DNA sources, other potential DNA origins also play a role in triggering the cGAS-STING pathway in cancer. Disruption of transcription networks by endogenous retrotransposon elements within the “viral response” process is observed to promote cancer progression.^156,157^ This process involves nucleic acids from activated retrotransposon elements participating in type I interferon responses through STING and mitochondrial antiviral signaling protein (MAVS) in tumor cells.^158^ Retrotransposon elements may compromise genome integrity, indirectly enhancing cGAS-STING activation, suggesting a potential strategy for enhancing cancer immunogenicity via epigenetic drugs.^158^

In conclusion, various forms of “foreign DNA” are commonly implicated as promoters of cancer development and intrinsic immune triggers within cells. These instances underscore the significance of abnormal DNA in cancer immunity; however, further research is warranted to deepen our understanding of how tumor-related processes influence the immunostimulatory properties of endogenous DNA. For example, downregulation of TREX1 expression in tumor cells may elevate DNA accumulation, ribosomal collisions, and translational stress, thereby augmenting cGAS-dependent DNA recognition.^138^

### Involvement of cGAS-STING pathway in non-tumor cells

**Fig. 2:**
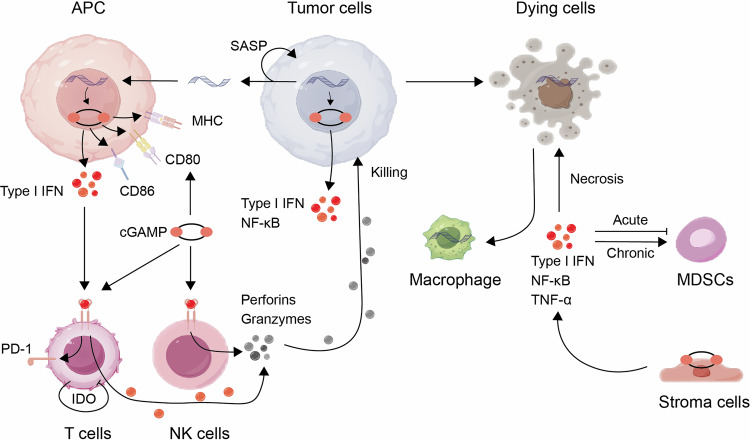
Regulation of tumor immune efficacy by the cGAS-STING pathway. Activation of the cGAS-STING pathway in tumor cells plays a crucial role in inducing the secretion of cytokines and chemokines, thereby promoting the immune-mediated elimination of early-stage tumor cells. Additionally, tumors have the capability to produce cGAMP, which initiates the transcription of STING in neighboring cells within the tumor microenvironment (TME). Following uptake of abnormal extracellular DNA from dying tumor cells, dendritic cells (DCs) and macrophages engage directly with cGAS. This interaction results in increased expression of co-stimulatory molecules (CD80 and CD86) and MHC molecules in these immune cells, enhancing their capability to activate a cytotoxic T-cell response. By releasing type I interferons, antigen-presenting cells (APCs) augment the cytotoxic potential of natural killer (NK) cells. Furthermore, cGAMP mitigates immunosuppression by inhibiting the recruitment of M2 macrophages and myeloid-derived suppressor cells (MDSCs). Conversely, sustained activation of the STING pathway suppresses dendritic cells (DCs) while attracting myeloid-derived suppressor cells (MDSCs), thereby tilting the balance towards an immunosuppressive tumor microenvironment (TME). Moreover, the involvement of STING in stromal and endothelial cells elicits anti-tumor effects by enhancing the inflammatory milieu, attracting immune cells, and guiding tumor necrosis. The cGAS-STING signaling pathway exhibits a dual role in both promoting and inhibiting tumor growth, with its effects predominantly influenced by the intensity and duration of the stimuli. In this context, black arrows represent promotion, while black bars symbolize inhibition. This figure was created using Figdraw

The DNA accumulated in apoptotic tumor cells and non-neoplastic cells within the tumor microenvironment (TME) is believed to act as the initiator for the cGAS pathway **(**Fig. 2**)**.^159^ This has led to proposals for enhancing the activation of the cGAS-STING pathway in the immune response against tumors through radiotherapy, targeted therapy, and ICB therapy. While there is an understanding of the relationship between DNA release from dying cells and the triggering of cGAS-STING signaling in phagocytic cells, the precise regulatory mechanisms remain unclear.^160^ The transfer of engulfed tumor cell fragments by macrophages to DCs rather than to other macrophages may contribute to the immunostimulatory effects. The efficiency of cell corpse processing in DCs may be relatively low, potentially leading to DNA leakage into the cytoplasm and subsequent immune stimulation.^161^ The interaction between cGAS and other innate signaling pathways, particularly the recognition of DNA by Toll-like receptor 9 (TLR9), plays a crucial role in the immune response to apoptotic cells.^162^ Tissue-resident macrophages specialized in clearing apoptotic cells exhibit limited responsiveness to TLR9 stimulation in vivo.^163^ This limited responsiveness may extend to tumor-infiltrating macrophages, explaining the heightened effectiveness of the cGAS-STING pathway compared to the TLR9 cascade upon exposure to extracellular DNA released by apoptotic tumor cells within the TME.^163,164^

### Intercellular cGAMP signaling in the tumor microenvironment

The intercellular transfer of diffusible cyclic dinucleotide cGAMP facilitates a variety of cGAMP-dependent effects within the tumor microenvironment (TME) (Fig. 2). Tumor cells have the capability to transmit cGAMP directly to astrocytes or dendritic cells via gap junctions, thereby influencing either inhibitory or promotive effects on tumor growth.^165^ Moreover, continuous activation of cGAS within tumor cells leads to the constant release of cGAMP into the extracellular space, consequently promoting STING transcription activation in adjacent immune cells.^166^ It is important to note that the ability of tumor cells to secrete cGAMP is significantly heightened by ionizing radiation (and other cancer therapies), which significantly contributes to the rationale behind the STING-dependent immune response against the tumor.^166^ The movement of cGAMP into and out of cells is facilitated by specific transmembrane channels that vary between cell types. Notably, members of the solute carrier group such as SLC19A1 and SLC46A2 play a crucial role in facilitating the entry of cGAMP into diverse cell populations like monocytes and macrophages. Conversely, chloride channel complexes are pivotal in controlling the uptake of cGAMP in vascular cells and bone marrow-derived macrophages.^167–169^ Regulating the influx and efflux of cGAMP provides an additional mechanism to fine-tune the innate STING responses within cancer cells, subsequently influencing immune responses in the TME. For example, the expulsion of cGAMP from malignant cell cytoplasm may attenuate internal STING signaling.^141^ Dendritic cells (DCs) and macrophages possess the ability to internalize external DNA released from apoptotic tumor cells, leading to direct interaction with cGAS. This interaction enhances the expression of co-stimulatory markers (such as CD80 and CD86) and MHC molecules, thereby bolstering their capacity to activate cytotoxic CD8^+^ T cells.^161,170^

### Tumor-suppressing and promoting functions of cGAS-STING

In the realm of cancer, the cGAS-STING pathway plays a pivotal role in facilitating a variety of functions that may sometimes be conflicting. These functions have a significant impact on tumor immunogenicity, influencing both the responses of tumor cells and the communication between tumor cells and nearby cells in the TME **(**Fig. 2**)**. The cGAS-STING pathway is crucial for enhancing therapeutic antitumor immune responses, particularly through the triggering of a robust IFN response. This response is crucial for activating immune cells, particularly DCs, present in the tumor microenvironment.^171^ Notably, this pathway’s activation has been observed in DCs, T cells, and NK cells, indicating a broad effect.^172^ Apart from IFN, cytokine signals regulated by NF-κB are also vital for modulating tumor growth as they work synergistically with IFN responses to boost the activity of NK cells in controlling tumor progression.^173^ Furthermore, the expression of cGAS and STING is not limited to immune cells but also extends to stromal cells, where STING triggers a cytokine response that enhances the inflammatory environment, leading to tumor necrosis and additional anti-tumor effects.^174^

The cGAS-STING pathway is critical in various aspects of cancer progression, with a key role in regulating the senescence-associated secretory phenotype (SASP).^175^ This signaling pathway functions to suppress the proliferation of damaged cells and improve the elimination of precancerous cells by immune cells through cytokine signaling.^176^ Cells that manage to evade senescence face additional hurdles in transitioning to cancer, including replicative crisis characterized by telomere shortening, chromosomal abnormalities, and significant cell mortality. The enzyme cGAS, serving as a detector of telomeric DNA damage, is believed to promote cell death linked to replicative crisis. This process relies on STING’s enhancement of autophagy rather than its involvement in cytokine signaling.^177^ The full cGAS-STING signaling pathway inhibits tumor development by stimulating the production of NK cell ligands (e.g., RAE1) on the cell surface as tumor cells advance.^178^ Recent studies have demonstrated that the cGAS-STING signaling pathway autonomously triggers a direct anti-proliferative effect or cellular apoptosis, particularly notable in T-cell leukemia.^179,180^ These responses boost the production of IFN and chemokines crucial for tumor cells, fostering an immune stimulating milieu within the tumor microenvironment.^181^

The cGAS-STING signaling pathway has been implicated in promoting tumor proliferation and metastasis. Initial studies showed that mice lacking STING displayed resistance to carcinogen-induced skin cancer.^182^ The continued engulfment of dying cells by phagocytic cells is believed to trigger inflammatory cytokines, thus contributing to the pro-tumor effect of STING. This observation is consistent with the widely accepted concept that untreated chronic inflammation aids in cancer development.^183^ The cGAS-STING pathway not only initiates cancer but also stimulates pro-tumor actions post-tumor formation. In cases of increased chromosomal instability in tumors, persistent activation of the cGAS-STING pathway promotes invasion and metastasis by transitioning from type I interferon and classical NF-κB signaling to noncanonical NF-κB pathways.^150^ Studies have shown that non-traditional NF-κB signaling pathways or prolonged type I interferon stimuli can dampen the anti-tumor immune responses triggered by radiation, achieved through dendritic cell suppression and myeloid-derived suppressor cell enhancement.^184^ The immunosuppressive effects are counteracted by cGAMP, inhibiting M2 macrophage and MDSC recruitment. Conversely, continuous STING pathway activation impairs dendritic cell function and facilitates myeloid-derived suppressor cell mobilization, creating an immunosuppressive tumor environment. Moreover, within stromal and endothelial cells, STING-mediated responses aid in enhancing the anti-tumor effect by boosting the inflammatory setting, attracting immune cells, and guiding tumor necrosis. Thus, ongoing STING pathway stimulation in tumors may advance cancer progression by altering the immune-suppressive tumor environment.

In acute scenarios, lymphocyte depletion caused by STING-induced cell death responses can impede tumor clearance. Studies on tumor transplants suggest that tumor cells effectively exploit the cGAS-STING pathway to eliminate T cells, possibly through cGAMP release.^185,186^ Similarly, endothelial cells exhibit a tendency to undergo apoptosis when exposed to cGAS-STING triggers.^187^ The documented immune response modulation by various cell types may diminish the therapeutic efficacy of STING activation against tumors. The overall impact of STING on tumor progression and regression is heavily influenced by the surrounding microenvironment, the timing, and the intensity of STING activation. The response of target cells to STING stimulation plays a critical and distinct role in shaping treatment outcomes. Consequently, the overall impact of STING on tumor progression and regression is shaped by these factors. Acute and moderate STING involvement is beneficial for inhibiting tumor effects, while prolonged or excessive STING activation results in immune suppression and adverse outcomes. Furthermore, targeting STING in dendritic cells while avoiding T cell exposure is crucial to enhance persistent tumor-specific T cell responses. This knowledge is currently being applied to enhance the effectiveness of STING agonist treatments in cancer immunotherapy. Initially, the use of small molecule inhibitors to block cGAS or STING was primarily aimed at mitigating harmful inflammatory aspects linked to autoimmune disorders.^188^ Recent findings suggest that targeting cGAS or STING inhibition could play a pivotal role in preventing inflammation-induced tumor progression or serve as a therapeutic strategy against metastasis in cancers with high chromosomal instability.^189^ Lastly, cGAS is also believed to influence tumor progression and suppression independently of STING. It has been proposed that cGAS accelerates tumorigenesis and increases DNA damage by interfering with homologous recombination DNA repair.^190^ Conversely, the absence of cGAS, regardless of STING, compromises the integrity of the intestinal barrier and impedes colon cancer progression, underscoring cGAS’s unique role in guarding against inflammation-induced tumorigenesis.^191^

### New paradigms in treating cancer with cGAS-STING

Research on the anti-tumor properties of STING agonists obtained from cGAMP has highlighted the crucial role of IFN in their effectiveness against tumors.^192^ It has been found that the production of type I interferon by dendritic cells is essential for eliciting endogenous T cell responses. Additionally, endothelial cells have been observed to contribute to local type I interferon responses.^193^ In addition to promoting activated immune phenotypes, cGAMP has shown the ability to counteract immune-suppressive phenotypes, such as polarizing M2 macrophages.^161^ Therefore, targeted manipulation of innate immune cells, particularly dendritic cells, to avoid non-specific targeting within the tumor microenvironment, requires further investigation. Current research suggests encapsulating cGAMP into extracellular vesicles derived from viruses or through genetic engineering as a strategy to selectively target antigen-presenting cells to prevent immune cell dysfunction. Moreover, nanocarriers containing STING agonists have demonstrated improved efficacy in preclinical cancer models. These advancements hold promise for enhancing drug bioavailability, optimizing pharmacokinetics, and enabling systemic delivery.^194^ While challenges persist in utilizing STING agonists as adjunct therapeutic approaches, significant progress has been achieved in this realm. Therapeutic mRNA vaccine strategies leveraging the STING pathway to enhance tumor immunogenicity have been explored. This approach involves customization of specific lipid components for mRNA delivery, independent of cGAS activation of STING.^195^ Furthermore, the use of mRNA encoding cGAS or STING has been found to activate antigen-presenting cells involved in tumor infiltration, enhancing IFN signaling pathways to enhance CD8^+^ T cell responses.^196^ Despite the highest adjuvant activity observed in patients with the STINGV155M mutation associated with early-onset STING-associated vasculopathy of infancy (SAVI), potential T cell cytotoxic side effects should be considered.^196^

Various cellular protective mechanisms along the DNA-cGAS-cGAMP-STING pathway have been explored as potential therapeutic targets, in addition to STING agonists.^138^ Upstream, enhancing DNA levels by inhibiting the degradative activity of extracellular nucleases like TREX1 has been shown to improve tumor control by radiotherapy. Altering radiation dosage protocols has been demonstrated to enhance type I interferon production, dendritic cell activation, and endogenous cytotoxic T lymphocyte responses against tumors by preventing TREX1 induction.^138^ Cancer cells have been found to downregulate cGAS or STING expression to effectively evade immune surveillance, inhibiting intracellular pathway activation. This has led to the development of strategies to reactivate natural cGAS-STING signaling in tumor cells, such as introducing mRNA encoding STING or utilizing pharmaceutical agents to antagonize epigenetic suppression mechanisms to enhance cancer cell immunogenic properties.^197^ Another strategy to boost STING activation in the tumor microenvironment is by inhibiting ENPP1, an enzyme that degrades extracellular cGAMP.^198^ Initial studies have indicated that genetic knockout or pharmacological inhibition of ENPP1 increases tumor immunogenicity. Enhancing local anti-tumor immunity by targeting ENPP1 significantly reduces systemic toxicity resulting from excessive cGAMP degradation. Furthermore, blocking the anti-phagocytic signal mediated by CD47 can activate STING, leading to dendritic cell stimulation and CD8^+^ T cell activation.^199,200^ Blocking TIM3 can enhance DNA uptake by dendritic cells and modulate STING-mediated immune signaling.^48^ Similarly, defects in LC3-related autophagic processes are crucial for cellular balance and immune responses and can enhance T cell activation in a STING-dependent manner.^201^ Effective integration of cancer treatment methods that activate cGAS-STING activity is essential for treatment optimization.

The diverse roles of the cGAS-STING pathway as an innate immune signaling pathway in detecting intracellular imbalances and initiating strong anti-tumor immune responses have garnered considerable attention. Moreover, the interplay between cGAS-STING and cancer is intricate, encompassing functions in immune suppression as well as facilitation of metastasis. Researchers have commenced the development of next-generation STING agonists with the goal of creating pharmaceuticals possessing enhanced bioavailability and tailored toxicity profiles. The prospect of translating these research findings into clinical applications is indeed promising.

## Epigenetics: intrinsic factors in tumor “cold-to-hot” transition

The innate immune system serves as the primary defense mechanism against invading pathogens, predominantly relying on epigenetic regulatory pathways to facilitate rapid immune responses that are independent of previous pathogen exposures.^202^ Epigenetic mechanisms play a crucial role in the differentiation and function of immune cells, allowing for the control of gene activity in response to diverse environmental signals across various tissue types. These mechanisms are essential for regulating the diversity of immune cells and stromal cells within the TME, profoundly influencing its cellular composition.^16^ Notably, epigenetic markers in tumor cells are increasingly recognized as reliable prognostic indicators for patients undergoing immunotherapy. Furthermore, combining epigenetic treatments with other therapeutic approaches can exploit the changing nature of epigenetic modifications, potentially transforming immunological characteristics of “cold” tumors into “hot” tumors.^203,204^ Identifying specific epigenetic biomarkers also provides a foundation for patient stratification and personalized treatment, thereby increasing the chances of successful treatment outcomes.

### Epigenetic biomarkers: a cost-benefit analysis

Molecular biomarkers are indeed crucial in predicting the response to tumor immune therapy, especially in the realm of personalized cancer immunotherapy.^205^ Understanding the regulatory mechanisms of epigenetic events sheds light on the significance of specific epigenetic changes as potential biomarkers for immunotherapy.^206,207^ Inherent epigenetic modifications within tumor cells are strongly linked to cancer progression, development, and resistance to treatment.^208,209^ Additionally, it is important to note that therapy itself can trigger epigenetic changes, such as variations in DNA methylation patterns of CD8^+^ T cells post-immunotherapy.^210^ Epigenetic biomarkers offer numerous advantages, including minimal invasiveness, the possibility of liquid biopsy, and the ability to measure DNA methylation changes in body fluids.^211^ Particularly in diseases like lung cancer, epigenetic biomarkers can fulfill various roles as diagnostic indicators, prognostic factors, predictive markers, and tools for treatment monitoring. This drives progress in therapeutic diagnostics and precision medicine. While the potential of using epigenetic changes as biomarkers is substantial, it is crucial to exercise caution and adhere to specific prerequisites. Testing candidate epigenetic biomarkers in well-defined and homogeneous patient cohorts is essential. This necessitates the development of sensitive and precise detection methods to uncover novel epigenetic features that could further enhance our understanding of cancer biology and treatment outcomes.

### Epigenetic alterations in tumor and immune cells

The development of cancer is primarily driven by genetic mutations and abnormal changes in chromatin structure, disrupting the usual functioning of cells and potentially initiating and fostering tumor growth.^212^ Common mutations found in cancer often involve alterations in the activity of chromatin-modifying enzymes, stemming from abnormal expression or mutations.^213,214^ Tumor cells frequently exhibit heightened acetylation of promoters, leading to the overexpression of oncogenes. Many cancer types also display a widespread loss of DNA methylation, with a particular trend towards gaining DNA methylation at CpG island sites.^215^ As tumor cells undergo significant alterations in the epigenome and the chromatin landscape of the tumor microenvironment and immune cells, the regulation of the intensity and efficacy of anti-tumor immune responses come into play. This intricate interplay may have implications for responses to immunotherapy and overall disease outcomes.^216,217^ Immune suppression within the tumor microenvironment can promote tumor progression and impact immune cells at an epigenetic level, potentially facilitating tumor evasion.^218^ In tumors characterized as “hot” effective anti-tumor immune responses, including those involving helper type I T cells and linked to the interferon response, are observed in malignancies like melanoma and head and neck squamous cell carcinoma (HNSCC).^219,220^ Therefore, understanding how tumors influence immune suppressive tumor microenvironments is pivotal. CD8^+^ T cells commonly exist in a state of exhaustion due to prolonged antigen stimulation,^221^ featuring upregulation of genes like PTCD1 (encoding PD-1) and activation of the IL-10 signaling pathway.^222,223^ Recent clinical evidence underscores that solely applying anti-PD-1 antibodies locally is insufficient to fully reverse CD8^+^ T cell exhaustion.^224^ Emphasizing the necessity of bolstering a robust and effective immune response against tumors, it is crucial to recognize that, as tumors progress, CD8^+^ T cells targeting specific tumor antigens may experience dysfunction, as evidenced by the release of IFN-γ and TNF-α.^93^ Additionally, the accumulation of double-stranded RNA (dsRNA) in tumor cells can trigger IFN responses in the tumor microenvironment, bolstering a potent antitumor immune response.^225^ Inhibiting the functions of methyltransferase G9a and DNA methyltransferases (DNMTs) in ovarian cancer cell lines can enhance the expression of endogenous retrovirus (ERV) transcripts, consequently prompting the activation of virus defense genes such as IRF7 and STAT1.^226^ Notably, treating colon cancer cell lines with the demethylating agent 5-Azacytidine (5-AZA) boosts the expression of interferon response factors like IRF7 and OASL by increasing dsRNA levels and initiating the MDA5/MAVS/IRF7 signaling cascade.^227^ Therefore, leveraging epigenetic therapies to target tumor cells may induce a viral-like response, enhancing antitumor reactions and improving therapeutic outcomes when combined with ICB therapy.

### Epigenetic modifications as promising biomarkers for immunotherapy

**Fig. 3:**
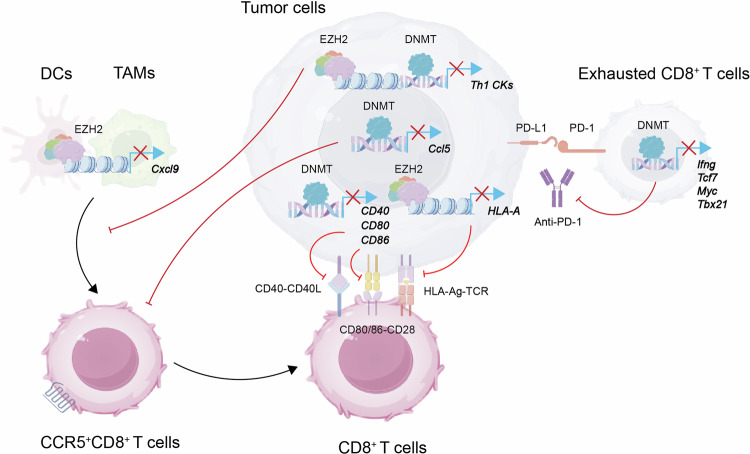
Epigenetic modulation and tumor immune efficacy. DNMT1 and EZH2 play critical roles in DNA and histone methylation, respectively. This epigenetic modification leads to the downregulation of chemokine genes *Cxcl9* and *Cxcl10*, impeding the recruitment of CD8^+^ T cells. Reduced *Cxcl9* secretion by antigen-presenting cells (APCs) following interferon (IFN)-γ exposure is associated with *Ccl5* methylation in cancer cells, resulting in diminished CD8^+^ T cell infiltration. Leukemia inhibitory factor (LIF) promotes the recruitment of EZH2 to the *Cxcl9* promoter in tumor-associated macrophages (TAMs), contributing to epigenetic silencing. The formation of immunological synapses and antigen presentation is essential for mounting effective cytotoxic responses against tumors. Nevertheless, epigenetic mechanisms, notably DNA methylation, can silence this process within tumor cells. Methylation of genes in PD-1^+^CD8^+^ T cells may induce an exhaustion state, leading to resistance to therapies targeting the PD-1 pathway, such as anti-PD-1 antibodies. In this context, black arrows represent promotion, while red bars symbolize inhibition. This figure was created using Figdraw

Epigenetic biomarkers linked to immune evasion or response traits are valuable indicators for evaluating the efficacy of immunotherapy.^228^ While most studies have focused on exploring at the tissue level, a deeper understanding at the cellular level is still ongoing (Fig. 3). Employing transcriptomic and epigenomic data for systematic analysis proves to be a powerful method in discovering epigenetic markers. Through comprehensive whole-genome bisulfite sequencing (WGBS) on virus-targeting CD8^+^ T cells from a mouse model with persistent infection by LCMV strain 13, the DNA methylation profile regulated by the newly generated DNMT3a for important genes (such as *Ifng, Myc, Tcf7, Ccr7*, and *Tbx21*) was revealed, with this methylation pattern associated with fatigue.^229^ DNA methylation analysis has unveiled abnormal activation of the TGF-β pathway in fibroblasts associated with tumors, potentially leading to immune suppression in initially “hot” tumors and hindering effective responses to ICB.^230^ In a study focusing on the cohesive tissue of tumor cells in the murine ID8 model of ovarian cancer, inhibiting the epigenetic controllers EZH2 and DNMT1 increased expression levels of Th1 chemokine genes *Cxcl9* and *Cxcl10* within the tumor cells.^62^ Through concurrent application of epigenetic and immunotherapy via inhibition of EZH2 and DNMT1, enhanced infiltration of CD8^+^ T cells and improved efficacy of the anti-PD-L1 antibody in treatment were observed.^231^ Epigenetic regulation has the potential to counteract immune avoidance due to epigenetic suppression of Th1 chemokine gene expression.^231^ Inhibiting the LSD1 histone demethylase gene in melanoma cells resulted in increased penetration of CD4^+^ and CD8^+^ T lymphocytes in tumor tissues, enhancing tumor immunogenicity compared to normal controls.^232^ In ovarian cancer in humans, heightened expression of *CCL5* and *CXCL9* led to increased infiltration of CD8^+^ T cells into tumors, prolonging patient survival outcomes and augmenting the effectiveness of anti-PD-1 antibody ICB treatment compared to cases with lower chemokine expression.^62^ Methylation status of *Ccl5* DNA in ID8 mouse ovarian cancer tissues led to reduced attraction of tumor-infiltrating lymphocytes and macrophages, presenting a ‘cold’ tumor characteristic with impact on animal survival and anti-PD-1 antibody treatment response.^62^ In a small-cell lung cancer (SCLC) mouse model, tumor cells showed increased resistance to CD8^+^ T cells by inhibiting MHC-I genes through PRC2, while inhibiting EZH2 restored HLA gene expression^42^. Melanoma cells treated with IFN-γ displayed elevated STAT-1 binding at sites such as MHC-II and CD274, dampening the effectiveness of anti-CTLA-4 antibodies.^233^ Epigenetic mechanisms regulate molecules like Adora 2 A and galectin-3 in HNSCC, influencing immune modulation and suggesting potential as biomarkers for patient stratification.^234^ Modulation of TNFRSF9 expression in immune cells infiltrating tumors through DNA methylation boosts anti-tumor immune responses, associated with extended progression-free survival (PFS) and positive responses to PD-1 antibody treatment.^235^ These findings support the use of epigenetic “biomarkers” and transcriptomic programs to enhance or synergize with anticancer therapies, particularly in immunogenic malignancies like melanoma.In immunogenic malignancies such as melanoma, the DNA hypomethylation of genes associated with the immune synapse, namely *HLA, CD40, CD86*, and *CD80*, contributes to the attraction of effector CD4^+^ and CD8^+^ T cells. Additionally, this mechanism regulates immune tolerance within the TME.^236^ The upregulation of Ezh2 expression in the tumor has been demonstrated in three distinct melanoma mouse models (B16-F10, RIM3, and NrasQ61KInk4a^−/−^) following anti-CTLA-4 antibody therapy.^237^ The upregulation leads to the epigenetic silencing of immune-related genes in tumors, such as *Cxcl9*, in contrast to the control group. Consequently, the efficiency of antigen processing and presentation to immune cells is diminished.^237^ Additionally, a subgroup of CD8^+^ T cells with high PD-1 expression and decreased chromatin accessibility in the TCF7 locus was pinpointed using single-cell transcriptomic profiling. These cells are associated with the suboptimal outcomes observed in melanoma patients experiencing fatigue and unresponsiveness to checkpoint inhibitor therapies.^238^ This evidence may support the use of epigenetics as “biomarkers” and transcriptional programs to dissect potential drivers or complement treatment strategies for cancer therapy.

In clinical settings, patients undergoing immunotherapy often display related epigenetic modifications. A comparative analysis of histone modification profiles in human gastric tumors showed a decrease in tumor antigenicity due to altered H3 lysine 4 methylation and H3 lysine 27 acetylation, indicating an immune evasion mechanism regulated by epigenetic factors.^239^ A study on non-small cell lung cancer patients receiving treatment with EGFR tyrosine kinase inhibitors and nivolumab (an anti-PD-1 antibody) unveiled a correlation between the methylation levels of the PDCD1LG1 promoter in tumor cells and the development of resistance to anti-PD-1 antibody therapy.^240^ Furthermore, lower methylation levels of CTLA4 in malignant melanoma tissues have been linked to improved treatment outcomes with anti-PD-1 and anti-CTLA-4 antibodies, leading to enhanced overall survival rates.^241^ This underscores the significant role of epigenetics in predicting and influencing immune responses to immunotherapy. Another study identified a relationship between reduced DNA methylation and the presence of CD8^+^ T cells within tumors.^242^ Experiments with CAR T cells targeted against CD19 (CART19) demonstrated heightened cytotoxicity upon re-stimulation in laboratory tests and resulted in complete recoveries in patients.^243^ Additionally, distinct activation levels were observed at 2732 promoter sites associated with resistance to immune checkpoint inhibition by anti-PD-1 antibodies, suggesting specific promoters could potentially serve as predictive biomarkers for immunotherapy.^244^ Utilizing epigenetic changes that govern tumor-associated immune responses to bolster the effectiveness of immunotherapy in cancer patients holds potential and warrants additional investigation, given the myriad unknown factors, especially in forecasting responses among various tumor types.^245^ Recognizing the significance of identifying reliable biomarkers linked to favorable outcomes in immunotherapy, investigating epigenetic changes that might indicate treatment efficacy or predict responses could prove beneficial and help refine precise diagnostic and stratification strategies.

## Immune metabolism: the switch for tumor “cold-to-hot” transition

Tumor cells possess the ability to sense metabolic changes and initiate a cascade of responses involving cell signaling and epigenetic modifications.^246^ Since Warburg first identified aerobic glycolysis as a metabolic hallmark in these cells, researchers have dedicated significant efforts to probing the metabolic alterations of tumor cells.^247^ Metabolic reprogramming is a prevalent trait in tumors, enabling cells to adapt their glucose, lipid, and amino acid utilization to meet specific growth needs and modulate behaviors accordingly.^248^ Tumor metabolism serves a dual role by supporting tumor cell proliferation and shaping an immunosuppressive tumor microenvironment (TME).^249^ Within the TME, tumor cells release immunosuppressive by-products, deplete nutrients crucial for immune responses, and trigger various immunosuppressive mechanisms that impede the effector functions of anti-tumor immune cells.^18^ As a result, targeting cancer cell metabolism has emerged as a promising approach to counterbalance or reverse immune suppression in “cold” tumors, enhance intrinsic anti-tumor immune responses in patients, or optimize the efficacy of immunotherapeutic interventions.

### Competition for glucose within tumors drives immune suppression in the TME

**Fig. 4:**
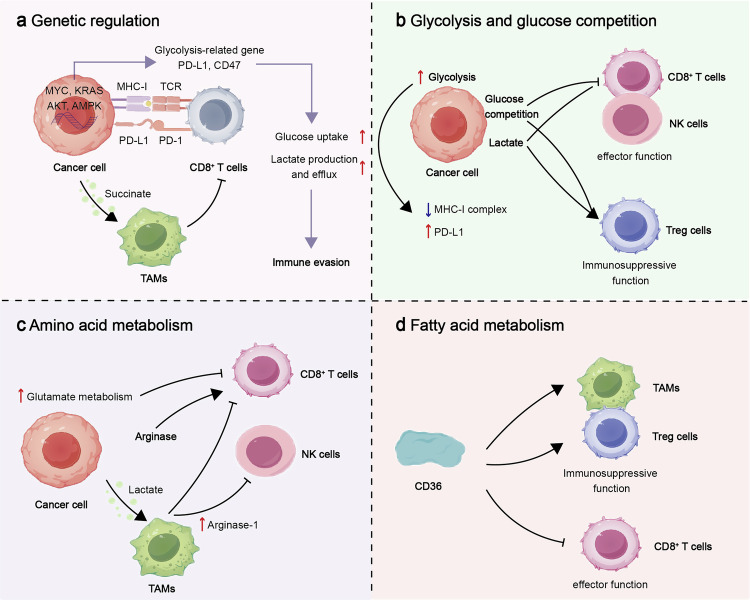
Illustrates the mechanisms of metabolic regulation in tumor immune evasion. Tumor cells and immune cells adapt to the tumor microenvironment by modifying their metabolic programs in response to conditions such as hypoxia and nutrient deprivation. **a** Tumor oncogenic signaling pathways and transcription factors play a crucial role in regulating the expression of immune checkpoint molecules and genes associated with glycolysis, ultimately contributing to tumor immune evasion. Additionally, metabolites can directly influence the expression of immunosuppressive molecules. **b** Dysfunctions in immune cells may arise due to alterations in metabolites. The upregulation of glycolysis in tumor cells affects the expression levels of MHC-I and PD-L1 proteins, while glucose deprivation and increased lactate levels inhibit the function of NK and CD8^+^ T cells but enhance the suppressive activity of Treg cells within the tumor microenvironment. **c** A competition in glutamine metabolism is observed in the tumor microenvironment, where enhanced arginine-sensing mechanisms support the survival of T cells. Furthermore, lactate produced by tumors can induce macrophages to shift towards the M2 phenotype, potentially leading to arginine deprivation in T cells and NK cells. **d** Tumor immune cells display distinct metabolic characteristics, with Treg cells and M2 macrophages maintaining their suppressive function facilitated by fatty acid transporters like CD36, while the presence of fatty acids hinders the effector function and viability of CD8^+^ T cells. In this context, black arrows represent promotion, while black bars symbolize inhibition. This figure was created using Figdraw

Metabolic shifts can induce exhaustion and dysfunction in effector immune cells, thereby influencing their differentiation (Fig. 4a, b). The progression of cancer triggers a conflict between immune cells and tumor cells, leading to decreased oxygen and glucose levels. In a series of cancer models, bone marrow cells exhibit the highest capability in absorbing glucose from the tumor, followed by T cells and cancer cells.^250^ Insufficient glucose can diminish MHC-I antigen presentation on tumor cells, reducing their responsiveness to IFN-induced cytotoxic actions.^251^ Similarly, glucose scarcity can impair the functionality of T lymphocytes infiltrating the tumor microenvironment.^252^ Specific transcription factors and oncogenic signaling pathways, including AKT,^253^ KRAS,^254^ and MYC,^255^ regulate immune checkpoint proteins like CD47 and PD-L1, impacting glycolysis-related gene expression and promoting immune evasion. Metabolites can directly influence the expression of immune inhibitory molecules.^256^ The function and viability of NK cells heavily rely on glycolysis, as evidenced by studies demonstrating NK cell dysfunction upon glycolysis inhibition and reduced fructose 1,6-bisphosphatase 1 (FBP1) expression due to TGFβ, affecting their survival.^257^ Moreover, compromised NK cell function arises from lipid peroxidation-induced oxidative stress impeding glucose metabolism.^258^ These findings underscore the critical role of glucose availability and glycolytic activity in supporting the effector functions of T cells and NK cells within the tumor microenvironment (TME).

In tumor cells, glycolysis converts excess pyruvate and NADH into lactate and NAD^+^ via lactate dehydrogenase A (LDHA) to produce ATP to some extent. In comparison to normal tissues, tumors exhibit an increased glycolytic flux, known as the Warburg effect. However, this increase is insufficient to compensate for the low flux through the TCA cycle in terms of ATP production.^259^ Consequently, solid tumors typically generate ATP at a slower rate than normal tissues, contrary to the widely held perception of them being highly metabolically active. Accumulation of lactate in highly glycolytic tumors impedes CD8^+^ T cell cytotoxicity, facilitating immune evasion.^260^ Moreover, tumor microenvironment lactate presence affects genes linked to NAD^+^ salvage pathways, impacting NK cell cytotoxicity efficacy and persistence.^261^ High lactate levels at metastatic sites hinder T cell activation and NK cell function.^262^ Increased lactate uptake through monocarboxylate transporter 1 (MCT1) upregulates PD-1 in regulatory T cells, reducing effector T cell responsiveness to immune checkpoint blockade.^263^ Additionally, lactate disrupts anti-tumor M1 macrophage metabolic activities while reinforcing pro-tumor M2 macrophage inhibitory functions.^264^ Impaired glycolysis in TAMs induces hypoxia, affecting endothelial function, subsequently leading to metastasis and nutrient deprivation.^265^ Unlike glucose-deprived activated T cells, T cells in acidic TME face lactate export challenges via MCT1, impeding proliferation. Acidosis stimulates pro-tumor neutrophil activation, elevating microenvironment acidity through proton release.^266^ The complex metabolic interplay between lactate-producing and lactate-consuming cells in tumors significantly influences resistance to anti-angiogenic treatment and shapes immune responses against tumors.^267^

### Metabolism of amino acids and immune surveillance

Besides glucose, the availability of amino acids can significantly influence the activity and differentiation of immune cells (Fig. 4c). Effector T cells and glutamine-dependent tumor cells within tumors both rely on glutamine, indicating a competition for glutamine metabolism in the tumor microenvironment.^268^ Glutamine is crucial for fueling mTORC1 activity in T cells, a process essential for their differentiation into effector T cells by providing the required energy.^269^ Restricting external glutamine can prompt the differentiation of CD4^+^ naive T cells into Treg cells, even in conditions conducive to generating other T cell subsets. Supplementation with the glutamine-derived metabolite α-ketoglutarate can boost Th1 cell differentiation.^270^ On the flip side, interrupting the glutamine metabolism of tumor cells in a triple-negative breast cancer model can amplify the expansion and cytokine secretion of effector T cells.^271^ Research suggests that increasing arginine supply may help sustain effector T cell functionality, potentially augmenting the effectiveness of checkpoint blockade therapy synergistically.^272^ Upon activation, T cells exhibit heightened arginine metabolism through the regulation of arginase 2 (ARG2), facilitating the formation of central memory-like T cells characterized by prolonged longevity and robust anti-tumor effectiveness. Additionally, enhanced arginine-sensing mechanisms aid in preserving T cell survival.^272^ Thus, the absence of external amino acids in the tumor microenvironment could impact the development, function, and maturation of immune cells.

Tumors elevate levels of crucial catabolic enzymes like ARG1 or IDO1, leading to decreased arginine and tryptophan levels in the tumor. This depletion plays a vital role in regulating T cell differentiation and proliferation.^273^ Lactate originating from tumors can drive macrophage polarization towards the M2 phenotype, potentially inducing the upregulation of arginase-1 in macrophages, causing arginine deprivation in T cells and NK cells.^274^ Both M1 and M2 polarized macrophages utilize arginine, albeit through distinct metabolic routes. While M1 macrophages employ inducible nitric oxide synthase (iNOS), M2 macrophages utilize Arg1 to metabolize arginine.^275^ The anti-tumor efficacy is attributed to nitric oxide (NO) produced by iNOS, while metabolites generated by ARG1 promote tumor cell proliferation and suppress NO synthesis.^276^ Furthermore, glutamine utilization is implicated in regulating the M2 macrophage polarization process, suggesting that inhibiting glutamine synthetase can shift M2 macrophage polarization towards an M1-like phenotype.^277^ These findings underscore the critical role of nutrient provision in modulating immune cell proliferation and specialization, thereby influencing the suppressive environment of tumors.

### Fatty acid and lipid metabolism in immune evasion

Tumor cells disrupt not only the levels of glucose and amino acids in the tumor microenvironment (TME) but also impact the types and quantities of fatty acids (FAs) (Fig. 4d).^278^ Within the TME, infiltrating immune cells may adjust their metabolism to confront metabolic constraints by increasing lipid droplets and extracellular fatty acids. This metabolic adaptation facilitates the efficient function of CD8^+^ T cells even under low glucose and oxygen levels, as fatty acid breakdown is sustained by peroxisome proliferator-activated receptor (PPAR)-α signaling. This process helps counteract the decrease in CD8^+^ T cells caused by metabolic stress.^279^ Notably, linoleic acid plays a pivotal role in enhancing the metabolic flexibility of CD8^+^ T cells, protecting them from exhaustion and promoting a transition to a memory cell-like phenotype.^280^ The utilization of oxidized fats can lead to ROS accumulation associated with lipids, hindering CD8^+^ T cell activity by upregulating the expression of scavenger receptor CD36.^117^ Studies indicate that Treg cells show a preference for utilizing fatty acid oxidation^281^ or short-chain fatty acids derived from the microbiota to regulate Treg cells differentiation.^282^ In contrast to CD8^+^ T cells, Treg cells sustain their survival and immunosuppressive function through increased fatty acid usage mediated by CD36.^119^ Mechanistically, within the tumor milieu, Treg cells selectively upregulate CD36, which, in turn, enhances mitochondrial health via PPARβ signaling, allowing for Treg cells reshaping to better adapt to the lactate-rich TME.^119^ The immune suppressive function of Treg cells can be hindered by limiting fatty acid transportation using sulfosuccinimidyl oleate (SSO) or impeding fatty acid oxidation with etomoxir.^283^ Lipid synthesis is essential for Treg cells to accumulate intracellular lipids, supporting their function by providing glycolytic products for expansion.^284,285^ Notably, the resistance of Treg cells to PD-1 therapy is strengthened by their utilization and availability of fatty acids.^286^

Moreover, lipid-rich environments also impact myeloid cells. In liver metastasis contexts, M2 macrophages contribute to tumor progression by absorbing long-chain fatty acids released by tumor cells via the CD36 receptor.^287^ Accumulation of lipids in DCs triggers endoplasmic reticulum (ER) stress, activating the XBP1 factor and consequently impairing the presentation of tumor-associated antigens.^288^ Furthermore, DCs educated by tumor cells with elevated fatty acid synthase (FASN) levels exhibit defects in T cell activation.^289^ Glycerol, a crucial component for lipid synthesis, is derived from the glycolytic intermediate dihydroxyacetone phosphate (DHAP). Similarly, to DCs, TAMs activate through lipolysis by upregulating CD36 expression.^290^ In addition to conventional fatty acid uptake, TAMs can recognize β-glucosylceramide via the Ca2±dependent Mincle receptor. This interaction not only fosters a pro-tumor phenotype but also enhances cell survival by triggering the ER stress response.^291^ With the pivotal roles played by ATP citrate lyase (ACLY) in fatty acid metabolism, cholesterol synthesis, protein acetylation, and histone acetylation, there is growing interest in developing anticancer drugs targeting this enzyme.^292^

### Metabolism-guided immune editing

The critical role in metastatic diseases is determined by the heterogeneity of tumor cells and the immune response, impacting the spatiotemporal advantages of tumor cell subclones.^293^ The selective pressure from CD8^+^ T cells drives clonal evolution within tumor cells.^293,294^ Moreover, the fate of metastatic tumor cells, whether eradicated or forced into a dormant state, depends on the presence of CD8^+^ T cells at the metastatic site.^275,295^ Factors present in the tumor environment, such as cytokines and metabolic by-products, play a pivotal role in immune modulation, potentially influencing the interplay between the tumor microenvironment (TME) and immune surveillance. Studies suggest a close relationship between epigenetic regulation, cellular metabolic activity, and immune surveillance in the TME. Specific molecules that permeate cells, like α-KG, can induce changes in DNA methylation patterns, impacting T cell differentiation.^296^ Additionally, findings have demonstrated that certain metabolic compounds, such as palmitic acid, can prompt H3K4 methylation, thereby influencing transcriptional alterations in cells.^297^

As a key regulator of metabolism and epigenetics, MYC significantly influences the TME by modulating the expression of CCL9 and IL-23, which in turn alters the immunosuppressive stroma, affecting the regulation of innate and adaptive immunity.^298^ The presence of IFNγ can induce epigenetic reprogramming in melanoma cells, leading to a rapid upsurge in MYC expression levels.^299,300^ Additionally, type I interferons can reshape the characteristics of cancer cells by inducing the demethylation of the epigenetic regulator, KDM1B, thereby influencing the reprogramming of cancer cells during immunogenic chemotherapy.^301^ Consequently, metabolic regulations may drive tumor cells into a dormant state, impacting anti-tumor immune responses, and indicating an alternative potential mechanism of immune modulation driven by metabolic changes through epigenetic modifications.

Research indicates that IFNγ from T cells can trigger malignant metabolic reprogramming, including heightened aerobic glycolysis and increased MYC expression, through the FGF2 signaling pathway. Furthermore, the enzymatic activity of PKM2 is impeded, causing a reduction in NAD^+^ levels, leading to elevated beta-catenin acetylation and fostering cancer progression.^302^ Additionally, infiltrating Th2 cells with KRAS mutations in PDAC express IL-4 or IL-13 cytokines, activating the IL2rγ–IL4rα and IL2rγ–IL13rα1 receptor complex signaling via the STAT6 pathway, driving MYC-mediated glycolytic reactions.^254^ Moreover, evidence suggests that the release of IFNγ by T cells or NK cells can alter cellular lipid composition and fatty acid metabolism, influencing sensitivity to ferroptosis by modulating long-chain acyl-coenzyme A synthetase 4 expression levels.^118^ Throughout cancer progression, tumor cells undergo metabolic adaptations to ensure survival under diverse nutritional conditions, potentially affecting the immune system’s surveillance capabilities. In essence, cytokine release by immune cells can regulate metabolic cascades involved in immune evasion, emphasizing the role of metabolic reprogramming in immune modulation and cancer therapy.

### Targeting metabolism to realign the anti-tumor immune response

Current clinical trials are increasingly focusing on targeting metabolic activity as a therapeutic approach in cancer treatment, showing promising responses that bolster the effectiveness of cancer immunotherapy. Notably, inhibitors of IDO (NCT02752074),^303^ CD73 (NCT03794544),^304^ arginase (NCT01266018),^305^ ODC (NCT01059071),^306^ iNOS (NCT02834403),^307^ mutant IDH (NCT02719574),^308^ and (NCT02577406),^309^ have exhibited good patient tolerability and are progressing towards phase II/III trials (Table 1). Enhancing the efficacy of ICB therapy could be achieved by adjusting the metabolic profile of effector T cells, curtailing glycolysis, or reducing LDHA levels in cancer cells, thereby improving the functionality of activated CD8^+^ T cells in the tumor microenvironment.^310^ Studies have illustrated that ICB can suppress immune cell metabolism by inhibiting glycolysis while boosting fatty acid oxidation and lipolysis.^311^ The integration of metabolic interventions with ICB presents a novel avenue for enhancing anti-tumor efficacy. In hepatocellular carcinoma patients with urea cycle defects, the combined use of arginine restriction and GCN2 inhibition has shown significant inhibitory effects.^312^ Treatment with dichloroacetate (DCA) can enhance oxidative phosphorylation in p53-positive tumor cells, induce the expression of stress ligands (such as MICA/B), and strengthen the efficacy of CAR T cell or allogeneic NK cell therapy. These findings underscore the value of focusing on cancer metabolism to intensify the effectiveness of immunotherapy and mitigate relapse.^313^ In cases of refractory patients, highly resistant tumor cells may require the use of metabolic medications or imposition of nutrient restrictions to heighten the vulnerability of tumor cells to cytotoxic lymphocyte attacks.^313^ However, challenges persist in implementing metabolic-targeted therapies in clinical settings. For instance, co-administration of IDO inhibitors with ICB has not resulted in amplified therapeutic efficacy.^303^

**Table 1:** Clinical trials of targeting cancer metabolism

Phase	ICIs Combination Therapy (immune checkpoint)	Doses	[n.treatment]	OS	PFS	Disease	Trial	Status	Ref.
III	**Epacadostat (IDO1) + Pembrolizumab (PD-1)**	epacadostat (100 mg) orally twice daily plus pembrolizumab (200 mg) intravenously every 3 weeks	[n = 354]	/	4.7 m	Unresectable or metastatic melanoma	NCT02752074	Completed	^302^
	Placebo + Pembrolizumab (PD-1)	pembrolizumab (200 mg) intravenously every 3 weeks	[n = 352]	/	4.9 m				
II	**Oleclumab (CD73) + Durvalumab (PD-L1)**	durvalumab (1,500 mg) every 4 weeks in combination with oleclumab (3,000 mg) every 2 weeks	[n = 21]	/	/	Resectable NSCLC	NCT03794544	Completed	^303^
	Durvalumab (PD-L1)	durvalumab (1,500 mg) every 4 weeks	[n = 26]	/	/				
II	**ADI-PEG 20 (pegargiminase)**	pegargiminase (320 IU/m2) (36.8 mg/m^2^) intramuscular every 1 weeks	[n = 22]	/	/	SCLC	NCT01266018	Terminated	^304^
I	**DFMO (ODC)**	Difluoromethylornithine (DFMO) (500-1500 mg/m^2^) every day	[n = 21]	/	/	NB	NCT01059071	Completed	^305^
I/II	**L-NMMA (p-NOS) + Docetaxel**	Docetaxel (two doses of 75 and 100 mg/m^2^) and L-NMMA (seven doses of 5, 7.5, 10, 12.5, 15, 17.5, and 20 mg/kg), L-NMMA was given intravenously on days 1 to 5 every 3 weeks, and docetaxel was given intravenously after L-NMMA on day 1 every 3 weeks. One cycle of treatment was defined as each 3-week regimen.	[n = 35]	/	/	TNBC	NCT02834403	Completed	^306^
I/II	**Olutasidenib (mIDH1)**	Olutasidenib was administered orally in doses of 150 mg once daily, 150 mg twice per day, and 300 mg once daily.	[n = 32]	/	/	AML	NCT02719574	Completed	^307^
	Olutasidenib (mIDH1) + Azacitidine (mIDH1)	Olutasidenib was administered orally in doses of 150 mg once daily, 150 mg twice per day, and 300 mg once daily. Azacitidine (75 mg/m^2^) was administered subcutaneously or intravenously daily for 7 days on, 21 days off.	[n = 46]	/	/				
III	**Enasidenib (mIDH2)**	enasidenib 100 mg per day orally (continuous); subcutaneous (SC) azacitidine 75 mg/m^2^ per day for 7 days per cycle; LDAC 20 mg twice-daily SC for 10 days per cycle; IDAC 0.5 to 1.5 g/m^2^ per day intravenous (IV) for 3 to 6 days per cycle; or BSC only	[n = 158]	6.5 m	/	AML	NCT02577406	Completed	^308^
	Conventional care regimens (CCRs)	azacitidine, intermediate-dose cytarabine (IDAC), low-dose cytarabine (LDAC), or best supportive care (BSC) only	[n = 161]	6.2 m	/				

Targeting cancer metabolism poses challenges, as certain metabolic interventions may impede crucial processes relied upon by immune surveillance. Upon T cell activation, there is a shift to aerobic glycolysis, prompting increased glucose and glutamine uptake to accelerate proliferation and cytotoxicity. Nonetheless, inhibiting glycolysis may render T cells inactive. Furthermore, complete blockage of glutamine uptake in tumors could potentially foster Treg cells expansion, limiting the effectiveness of T cell therapy. The development of resistance to ICB can induce distinct metabolic conditions in tumors, characterized by diminished CD8^+^ T cell infiltration and reduced IFN-γ gene expression, potentially influencing the efficacy of metabolic treatments. Identifying unique metabolic nodes vital for cancer cells to evade the immune system is crucial. By doing so, we can determine the optimal timing for modifying immune and cancer metabolism to significantly enhance existing immunotherapeutic strategies. Subsequently, further exploration in this evolving field will establish crucial foundations for maximizing the efficiency of metabolic-targeted cancer therapies.

## Iron metabolism: a novel target for tumor “hot-cold” switch

Cell metabolism is a fundamental cornerstone for sustaining normal cellular functions, encompassing the processing of large biomolecules like glucose, fatty acids, and amino acids, as well as the utilization of trace elements.^246,248^ Among these elements, iron metabolism can notably regulate the activity of various cellular enzymes, acting as a vital co-factor essential for normal cell growth.^314^ Recent insights into iron metabolism within the tumor microenvironment have emerged.^315–317^ Iron contributes to the generation of reactive oxygen species, potentially inducing iron-dependent cell death or fostering malignant transformation.^318^ Following alterations, tumor cells regulate immune cell infiltration into the tumor microenvironment to fulfill their iron requirements, thus influencing tumor surveillance by the immune system.^319^ Therefore, our study investigates the impact of iron metabolism on the intricate metabolic processes within the tumor microenvironment. We seek to explore the potential of manipulating iron metabolism as a novel therapeutic strategy for cancer treatment, while also addressing ongoing clinical debates.

### Immunogenicity of ferroptotic tumor cells

**Fig. 5:**
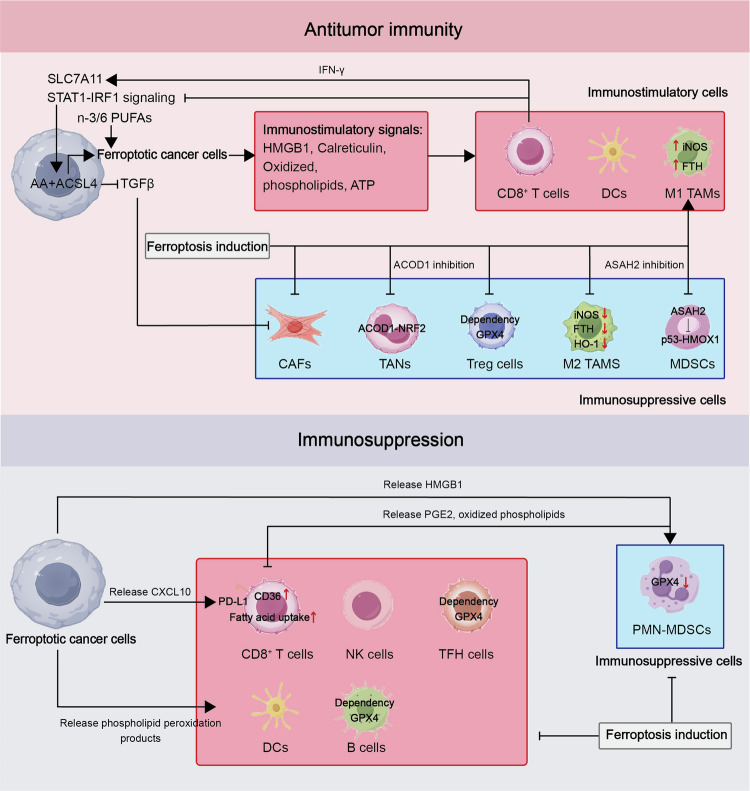
illustrates the dual role of ferroptosis in the tumor microenvironment. Concerning antitumor immunity, ferroptotic tumor cells release immunostimulatory signals that facilitate dendritic cell maturation, activate M1-polarized macrophages, and enhance T cell infiltration and activity within tumors. Both CD8^+^ T cells and neutrophils contribute to promoting ferroptosis in tumor cells. Ferroptosis in tumor cells alleviates the inhibition of cancer-associated fibroblasts (CAFs) by reducing TGF-b1 levels. Moreover, ferroptosis induction in various immunosuppressive cells, such as tumor-infiltrating neutrophils, myeloid-derived suppressor cells (MDSCs), regulatory T (Treg) cells, and M2-polarized tumor-associated macrophages (TAMs), boosts antitumor immunity. On the other hand, in terms of immunosuppression, ferroptotic tumor cells impede dendritic cell maturation through products of phospholipid peroxidation. Additionally, CXCL10 and HMGB1 released by ferroptotic cancer cells upregulate PD-L1 expression. The release of oxidized phospholipids and prostaglandin E2 (PGE2) by ferroptotic polymorphonuclear-MDSCs suppresses the function of CD8^+^ T cells. Furthermore, ferroptosis induction in various antitumor immune cells, including natural killer (NK) cells, B cells, and T follicular helper (TFH) cells, leads to inhibited antitumor immunity. In this context, black arrows represent promotion, while black bars symbolize inhibition. This figure was created using Figdraw

The release of immune stimulatory signals, such as high mobility group box 1, calreticulin, ATP, and oxidized phospholipids, by tumor cells undergoing ferroptosis is a key aspect of immunogenic cell death (Fig. 5).^320–324^ These signals play a vital role in promoting dendritic cell maturation and enhancing macrophage-mediated phagocytosis of ferroptotic tumor cells.^321,323^ For instance, oxidized phospholipids exposed on the surface of ferroptotic cancer cells can be recognized by Toll-like receptor 2 on macrophages, leading to increased engulfment of these cells.^324^ Studies have shown that these interactions can induce polarization of macrophages towards the M1 phenotype and potentially elicit responses akin to vaccines, thereby boosting anti-tumor immunity.^321,323,325^ Conversely, the inhibition of ferroptosis in tumor cells may hamper T cell infiltration and activity in the tumor microenvironment, dampening T cell-mediated anti-tumor reactions.^118,326^ Additionally, ferroptotic tumor cells could impede dendritic cell maturation and compromise their ability to present antigens, potentially negatively impacting adaptive immune responses.^327^ The impairment of dendritic cell maturation and function may be attributed to products of phospholipid peroxidation or other lipid peroxidation byproducts, such as 4-hydroxynonenal. Interestingly, in colorectal cancers lacking GPX4, there is an increase in dendritic cell infiltration, a phenomenon not observed in hepatocellular carcinoma.^328^ In conclusion, the debate surrounding the immunogenicity implications of ferroptotic tumor cells is context-specific and remains a topic of discussion in the field of ferroptosis research. Some studies have utilized ferroptotic tumor cells cultured in vitro as vaccination agents.^321^ However, achieving maximal efficacy in inducing ferroptotic cell death at the vaccination site may be challenging, potentially allowing viable tumor cells to persist and complicating tumor progression.

### Ferroptosis and innate immunity: balancing anti-tumor immune response

In the context of anti-tumor immunity, macrophages, neutrophils, and NK cells play pivotal roles in the immune response against iron-induced cell death (Fig. 5).^329^ Recent studies have revealed that M1 macrophages exhibit greater resistance to iron-induced cell death compared to M2 macrophages.^330^ This heightened resistance is attributed to the elevated expression of inducible nitric oxide synthase in M1 macrophages, leading to increased production of nitric oxide molecules. Nitric oxide radicals can effectively neutralize lipid radicals, thus protecting M1 macrophages from ferroptosis-induced damage.^331^ Iron nanoparticles have been shown to convert M2 macrophages into an M1 phenotype, enhancing their anti-cancer properties.^332–334^ Notably, the tyrosine protein kinase receptor TYRO3 significantly influences ferroptosis within the tumor microenvironment. Inhibiting TYRO3 attenuates iron-mediated cell death in cancer cells, resulting in a shift in the M1/M2 macrophage ratio and creating a tumor-promoting environment.^335^ Conversely, inhibiting TYRO3 promotes tumor ferroptosis, alters the M1/M2 macrophage balance, and improves the tumor response to PD-1 therapy.^335^ Several studies have elucidated the complex relationship between TAMs and ferroptosis. It has been suggested that ferroptosis can drive the recruitment and polarization of TAMs towards the M2 macrophage phenotype, while inhibiting ferroptosis may impede the development of an immunosuppressive tumor microenvironment.^336^ Factors that increase iron-induced cell apoptosis, such as elevated iron intake or deficiency of GPX4, could regulate macrophage migration and orientation through the STING-triggered DNA detection pathway, potentially impacting M2 macrophages and facilitating the progression of PDAC.^337^ These findings underscore the intricate and paradoxical effects of iron depletion on macrophages in the context of anti-tumor immunity.

Myeloid-derived suppressor cells (MDSCs) are well-known for their potent ability to induce immunosuppression.^338^ The resistance of MDSCs to ferroptosis is predominantly attributed to the inhibitory effect of n-acylsphingosine amidohydrolase 2 (ASAH2) on the signaling pathway involving p53 and heme oxygenase 1 (HO-1).^339^ Inhibition of ASAH2 can induce ferroptosis in MDSCs, enhance the infiltration of CD8^+^ T cells into tumors, and boost the inhibitory impact on tumor progression.^339^ Enhancing cancer immunotherapy efficacy could be achieved by combining ferroptosis induction and MDSC inhibition.^328^ Interestingly, restricted GPX4 loss in hepatocytes alone does not impede hepatocellular tumor formation; GPX4-related ferroptosis triggers immune-mediated tumor suppression in hepatocytes. Within the tumor microenvironment, neutrophils, polymorphonuclear-myeloid-derived suppressor cells (PMN-MDSCs), serve as crucial primary immunosuppressive agents. Genetically or pharmacologically inhibiting ferroptosis can abolish the suppressive function of PMN-MDSCs, resulting in reduced tumor progression. This strategy synergizes with immune checkpoint blockade to effectively impede tumor growth.^340^ Recent studies have demonstrated that tumor-infiltrating neutrophils with immunosuppressive characteristics, resembling PMN-MDSCs, manifest notable resistance to ferroptosis. This resistance may be associated with elevated levels of aconitate decarboxylase 1 (ACOD1) in neutrophils. Elevated ACOD1 levels upregulate aconitate decarboxylase expression, activating NRF2-mediated protective responses against ferroptosis. Conversely, ACOD1 deficiency suppresses neutrophil migration, augments the antitumor immune response, and enhances the efficacy of immunotherapeutic interventions.^341^ The vital need for a comprehensive understanding of the complex interplay between ferroptosis and immune cells in the tumor microenvironment is underscored by this discrepancy. Additionally, NK cells play a crucial role in the antitumor immune response.^342^ Dysfunction of NK cells in the tumor microenvironment is frequently linked to oxidative stress from lipid peroxidation. Activation of the NRF2 transcription factor could potentially rejuvenate NK cell function and bolster their effectiveness against tumors.^258^ Conversely, inhibiting ferroptosis may prolong the survival of NK cells in tumors.^340^ In essence, these investigations shed light on the susceptibility of NK cells to ferroptosis within the tumor microenvironment.

The collective results illuminate the intricate impact of ferroptosis on diverse innate immune cell subsets within the tumor microenvironment. The study highlights the resilience of M2 macrophages and infiltrating neutrophils to ferroptosis, the susceptibility of immune-stimulating NK cells to this process, and the nuanced responses of MDSCs to ferroptosis. A comprehensive comprehension of the complex interplay between iron metabolism in the tumor microenvironment and the innate immune system is vital for enhancing and refining approaches to cancer immunotherapy.

### Ferroptosis and adaptive immunity: the double-edged sword of antitumor immunity

**Fig. 6:**
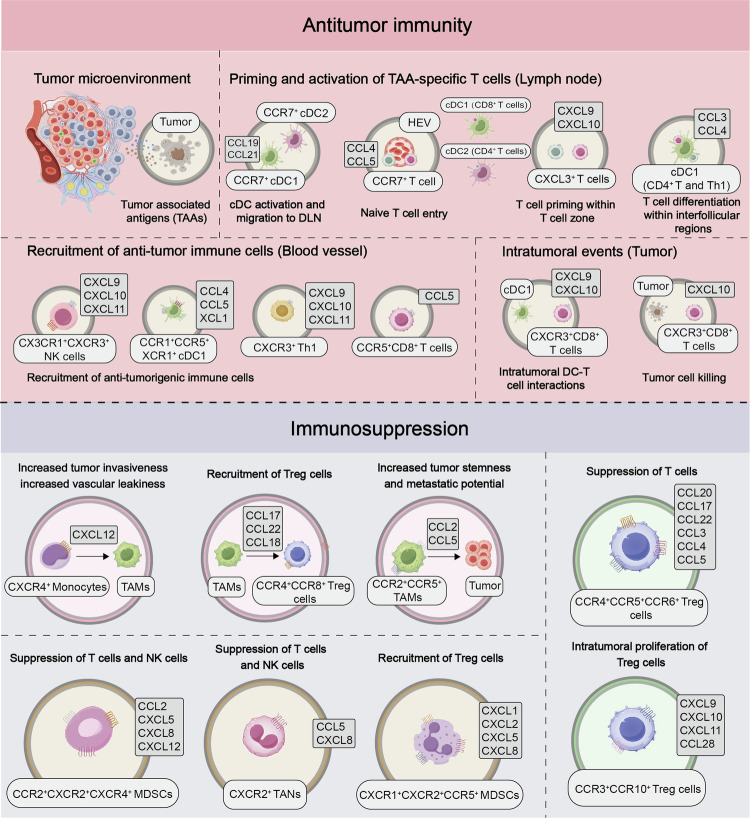
illustrates the impact of chemokines on shaping the tumor microenvironment (TME). To begin with, tumor cells release tumor-specific antigens (TAAs) and newly formed antigens designed to be captured and processed by professional antigen-presenting cells (APCs). Conventional dendritic cells (cDCs) undergo maturation and upregulate CCR7, facilitating their migration to lymph nodes that drain the tumor site. The chemotactic axis of CCR7-CCL19/CCL21 guides naïve CD8^+^ and CD4^+^ T cells towards these lymph nodes. Inside the lymph nodes, naïve T cells that recognize TAAs interact with both cDC1s and cDC2s, resulting in activation of CD4^+^ and CD8^+^ T cells, along with increased expression of CXCR3. This directs the activated T cells to specific regions known as interfollicular areas (IFRs) within the lymph nodes. Within the IFRs, CD4^+^ T cells specific to TAAs engage with dendritic cells through CXCR3-dependent mechanisms, promoting their transformation into Th1 cells. Immunological cells with anti-tumor properties, including natural killer (NK) cells, cDC1s, Th1 cells, and CD8^+^ T cells, enter the tumor microenvironment (TME) guided by chemotactic gradients originating from the bloodstream. cDC1s secrete CXCL9 and CXCL10 to attract CXCR3^+^CD8^+^ T cells and enhance the functions of intra-tumoral T effector cells. Activated CD8^+^ T cells and Th1 cells position themselves close to tumor cells to aid in the elimination of tumors either by producing cytokines or directly killing them. Additionally, within the TME, tumor-associated macrophages (TAMs) are attracted to the tumor site as activated monocytes through the chemokine pathways of CCR5-CCL5 and CCR2-CCL2. TAMs promote tumor progression by releasing CCL17, CCL22, and CCL18 to recruit CCR4^+^ and CCR8^+^ regulatory T cells (Treg cells). Interactions between TAMs and tumor cells via the pathways of CCR2-CCL2 and CCR5-CCL5 enhance tumor stemness and metastatic potential. Furthermore, myeloid-derived suppressor cells (MDSCs) and tumor-associated neutrophils (TANs) present in the TME inhibit T cells and NK cells while attracting Treg cells by secreting chemokines such as CCL3, CCL4, CCL5 (by MDSCs), and CCL17 (by TANs). Treg cells are also lured by various chemokine systems, playing a role in tumor growth by suppressing T cell responses within the TME. This figure was created using Figdraw

Within the tumor microenvironment (TME), the adaptive immune system is mainly composed of B cells and T cells, which play a crucial role in controlling tumor progression by reprogramming mechanisms.^343,344^ Iron-related products, such as lipid peroxides released by cancer cells, hold the potential to influence the functionality of adaptive immune cells and their ability to recognize tumor-specific antigens. Similar to innate immune cells, the response of adaptive immune cells to iron-induced products varies significantly based on specific conditions **(**Fig. 6**)**. B cells are pivotal in the immune response against tumors, as they produce anti-tumor antibodies and modulate T cell responses.^345^ Innate-like B cells, including B1 cells and marginal zone B cells, exhibit dynamic lipid metabolism. These cells’ vulnerability to ferroptosis triggered by GPX4 depletion stems from GPX4’s critical role in maintaining antibody response and overall cellular function.^346^ Further research is needed to fully grasp the significance of iron-induced cell death within the realm of B cell-driven anti-tumor immunity. This discovery points towards a promising avenue for exploring novel treatment strategies that target GPX4 in specific malignant B cell malignancies.

Conversely, T cells are less susceptible to ferroptosis, supporting the notion that selectively inducing ferroptosis in tumor cells does not significantly impede T cell-mediated anti-tumor immunity. In murine models, the depletion of SLC7A11 impedes tumor progression while leaving in vivo T cell expansion and anti-tumor immunity unaffected, thereby enhancing the efficacy of immune checkpoint blockade in preclinical studies.^347^ Inducing ferroptosis, activated by cystine deficiency or arachidonic acid introduction, can also impede tumor progression without affecting the expansion and functionality of T cells in the TME. On the contrary, blocking iron-induced cell death mediated by ACSL4 leads to a decrease in T cell-mediated immune responses against tumors.^348^ Additionally, stimulating ferroptosis through various nanoparticle-based methods enhances T cell infiltration in the tumor microenvironment.^349,350^ These findings underscore the potential enhancement of T cell-mediated immune responses towards tumors by simultaneously activating multiple pathways that induce ferroptosis.

T cells, particularly those lacking *Gpx4* due to T cell-specific *Gpx4* deletion in mice, exhibit heightened sensitivity to iron-induced cell death triggered by GPX4 inhibition. This increased sensitivity is attributed to the rapid accumulation of intracellular lipid peroxides, ultimately leading to cell death through ferroptosis.^351^ CD8^+^ T cells, in particular, show heightened sensitivity to iron-induced cell death caused by GPX4 inhibitors.^352^ Furthermore, the increased expression of CD36, a crucial player in enhancing the absorption of fatty acids in CD8^+^ T cells infiltrating tumors, may lead to lipid peroxidation and iron-mediated cell death in these T cells, thereby impairing their ability to combat tumors. Conversely, eliminating the CD36 gene or preventing iron-mediated cell death in CD8^+^ T cells has the potential to restore their antitumor function.^116,117^ The role of GPX4 in inducing ferroptosis also extends to CD4^+^ T cells.^353^ Regulatory T cells, a subset of immunosuppressive T cells, compromise the immune surveillance of tumors. Treg cells display inherent resistance to ferroptosis, possibly linked to their elevated GPX4 expression. Consequently, selectively deleting Gpx4 in Treg cells could enhance anti-tumor immune responses by instigating ferroptosis.^354^ Follicular helper T (TFH) cells, a specific subset of CD4^+^ T cells known for promoting anti-tumor responses, display susceptibility to iron-induced apoptosis, akin to Treg cells.^355^ Further investigation is necessary to comprehend the interaction between ferroptosis and TFH cells in the tumor microenvironment. Ferroptosis, induced by SLC7A11 depletion or GPX4 abnormalities, exerts unique effects on T cell function and the immune response to tumors. T cells seem to rely more on GPX4 than on SLC7A11, possibly due to the relatively low expression level and non-essential role of SLC7A11.^356,357^ Repressing SLC7A11 and/or stimulating iron-mediated cell death by depleting cystine or providing additional arachidonic acid seems to have no detrimental effects on the immune response against tumors by CD8^+^ T cells. Conversely, the iron-dependent cell death triggered by inhibiting GPX4 has varying effects on different T cell subpopulations: it enhances antitumor immune responses in the immunosuppressive Treg cells but dampens antitumor immune responses in CD8^+^ T cells and TFH cells.

### Ferroptosis in targeted immunotherapy

Recent research has revealed a significant discovery that inducing ferroptosis in tumors or enhancing their susceptibility to this process can significantly enhance the effectiveness of immunotherapy. Conversely, tumors that exhibit resistance to ferroptosis are also likely to be resistant to immunotherapy. A noticeable enhancement in the anti-tumor immune response, leading to a more efficient tumor inhibition, is observed when anti-PD-L1 therapy is administered concurrently with either cyst(e)inase or arachidonic acid treatment, as opposed to when each treatment is used individually.^118^ Similarly, the decreased expression of SLC7A11 increases tumors’ vulnerability to anti-PD-L1 treatment either alone or in combination with radiotherapy.^358^ Furthermore, periodic dietary restriction of methionine upregulates the glutathione-specific enzyme CHAC1, depleting GSH and exacerbating ferroptosis induced by cystine limitation, thereby rendering tumors more responsive to anti-PD-1 therapy.^359^ Surprisingly, the combined application of intermittent fasting targeting methionine, the SLC7A11 inhibitor IKE, and PD-1 therapy significantly inhibits tumor progression and prolongs the survival of animal subjects.^359^

A recent study has identified a beneficial relationship associated with the protein kinase C beta (PKCβII)-ACSL4 signaling pathway. In this process, PKCβII phosphorylates and activates ACSL4 to detect and enhance lipid peroxidation, ultimately contributing to the promotion of ferroptosis. Disruption of this signaling pathway impedes ferroptosis and the T cell-mediated immune response against tumors, leading to resistance to anti-PD-1 treatment by deleting PKCbII or ACSL4, or modifying phosphorylation sites of ACSL4.^360^ Preclinical investigations have shown that sensitizing tumors to immunotherapy by targeting GPX4 with FIN is feasible. Additionally, the synergistic effect of GPX4 inhibitors in combination with anti-PD-1/PD-L1 therapy has shown increased anti-tumor immune response and tumor inhibition.^361^ Exploring the potential integration of the newly developed effective GPX4 inhibitor JKE-1674 with anti-PD-1/PD-L1 treatment, which enhances in vivo drug metabolism, presents an enticing opportunity. Targeting ferroptosis in immunotherapy also involves other therapeutic approaches capable of inducing cancer cell ferroptosis. By suppressing phosphoglycerate mutase 1 (PGAM1), the downregulation of lipocalin-2 (LCN2) promotes ferroptosis in hepatocellular carcinoma cells and facilitates the recruitment of CD8^+^ T cells. Consequently, the combined inhibition of PGAM1 with anti-PD-1 therapy demonstrates promising synergistic effects in inhibiting tumor progression.^362^

In preclinical research, the bifunctional compound BEBT-908, targeting both PI3K and histone deacetylase, has been shown to induce immunogenic ferroptosis in cancerous cells. This mechanism involves enhancing p53 acetylation and inhibiting NRF2 activity, leading to the creation of an inflammatory microenvironment that promotes an anti-tumor immune response and enhances the efficacy of anti-PD-1 therapy.^363^ Furthermore, the effectiveness of immunotherapy has been demonstrated by certain nanomaterials possessing ferroptosis-inducing properties.^363,364^ For instance, in a preclinical model of hepatocellular carcinoma (HCC) with ascites, the use of an injectable hydrogel drug delivery system incorporating sulfasalazine has successfully induced significant immunogenic ferroptosis. Combining this strategy with anti-PD-1 treatment has synergistically inhibited the dissemination of malignant ascites in the peritoneal region.^365^ These findings highlight the potential of integrating immunotherapy with ferroptosis inducers in cancer treatment to enhance therapeutic outcomes.

When tumor cells undergo ferroptosis, they release signaling molecules such as arachidonic acid metabolites and HMGB1, which play a crucial role in enhancing the immune response against tumors by facilitating the recruitment of lymphocytes and activation of antigen-presenting cells. It is important to recognize that infiltrating immune cells within tumors can also release immunosuppressive molecules.^366^ Therefore, the overall outcomes depend significantly on the tumor-associated microenvironment. Notably, “cold” tumors are favorable for triggering ferroptosis, a process that can leverage the advantages of “immunogenic cell death” in cancer cells while mitigating immune suppression effects. Overcoming the limitations and toxicities associated with each approach is essential to ensure the efficacy of iron-targeted therapy as an effective treatment strategy. Anticipated advancements include the development of novel tools for detecting biomarkers related to iron metabolism in the tumor microenvironment, which will be instrumental in refining combined approaches to modulate iron levels in the TME and improve therapeutic outcomes for individuals with advanced cancer.

## Chemokines: intercellular communication in tumor “cold-to-hot”

Tumor metabolism is intricately linked to the dysregulated activities of immune cells and factors within the tumor microenvironment, such as chemokines and cytokines, ultimately resulting in tumor immune evasion. Chemokines exert a crucial influence on various aspects of tumor development. They affect the stemness, proliferative capacity, and invasiveness of tumor cells while also modulating key processes within the tumor microenvironment (TME), such as angiogenesis, neurogenesis, and fibrosis.^367,368^ By playing a pivotal role in coordinating the positioning and interaction between immune cells in the TME, these chemokines are expressed on immune, stromal, and tumor cells, enabling them to impact the stimulation, recruitment, properties, and functions of immune cells.^369,370^ This section primarily examines the significance of chemokines in cancer immunity, exploring their interactions within the TME along with the spatial and cellular heterogeneity. The signals mediated by these chemokines are essential for maintaining immune-cold features and are associated with the promotion of immune hot characteristics.

### Chemokines and tumor immune microenvironment

Tumors arise from acquired genetic mutations in normal cells, driving uncontrolled cell proliferation.^183^ The progression of tumors is often influenced by interactions with immune cells, fibroblasts, endothelial cells, and neurons within the tissue, which cancer cells exploit to their advantage. Furthermore, immune cells recruited to the site, exhibiting suppressive traits, aid in tumor evasion, reflecting behaviors similar to those observed in wound healing or tissue remodeling resolution stages.^371^ Various cell types within the tumor microenvironment play crucial roles in regulating cancer advancement, including TAMs, TANs, MDSCs, and Treg cells. While tumors comprise tumor cells themselves, their growth can be impeded by NK cells, which possess potent cytotoxic capabilities against distressed cells.^372^ Additionally, CD4^+^ Th1 cells and CD8^+^ T cells have the potential to eliminate cancer cells by recognizing tumor-specific or newly formed antigens.^373^ The initiation of anti-cancer T cell responses heavily relies on APCs, primarily DCs, that capture and process tumor-specific antigens to present to T cells.^374^ Throughout the process of tumor development, chemokines are indispensable for regulating the stimulation, mobilization, properties, and functions of immune cells within the tumor microenvironment (TME). Therefore, the substantial impact of chemokines and their receptors on the effectiveness of both tumor-promoting and tumor-inhibiting immune responses is clearly evident.

In “cold” tumors, myeloid cell infiltration may be present, yet CD8^+^ Teff cell infiltration is often lacking, indicating a reduced level of anti-tumor immunity. In this context, the activation of β-catenin in tumors inhibits the production of CCL4 by upregulating the transcription repressor ATF3, thus restricting the migration of conventional cDC1 towards the tumor, suggesting that tumors avoid immune surveillance by disrupting chemokine functionality.^45,375^ PGE2 diminishes the activity of NK cells, hindering their capability to produce inflammatory chemokines. Consequently, the abundance of cDC1 is limited, fostering tumor progression.^376^ Moreover, the decrease in cDC1 density within tumors over time implies that advanced tumors may possess the capacity to impede cDC1 migration.^374^ Whether a specific activation threshold is necessary to initiate CCR7-dependent migration of cDC1 towards tumors in sterile tissues remains unclear. Prior studies have highlighted the crucial involvement of atypical chemokine receptor 4 (ACKR4) in regulating DC ingress into lymphatic vessels and their localization in T cell regions within draining lymph nodes. This regulation is achieved through the modulation of the biological activity of CCL19 and CCL21, alongside the maintenance of functional chemotactic gradients.^377^ The impact of tumors on ACKR4 function may hinder DC infiltration into tumor-draining lymph nodes, though this effect remains uncertain. Certain tumors exhibit a limited presence of CD8^+^ T cells but attract substantial numbers of immunosuppressive cells like TAMs, MDSCs, and Treg cells.^378^ These tumors stimulate tumor growth by upregulating the expression of inflammatory mediators, creating a tumor microenvironment conducive to tumor survival. Notably, an elevated expression of NF-κB-dependent inflammatory genes is observed in the absence of the tumor suppressor p53.^379^ Studies suggest that NF-κB-driven chemokines may promote tumor progression, recurrence, and resistance to therapy. For instance, NF-κB activity induces the expression of CCL5, a chemokine that attracts macrophages expressing CCR5 and enhances collagen production, thereby promoting the proliferation of breast cancer cells.^380^ Activation of NF-κB in TAMs leads to the secretion of CCL22, which attracts CCR4^+^ Treg cells.^381^ Additionally, TAMs induce the release of CCL20 through NF-κB activation, recruiting CCR6^+^ Treg cells.^382^

“Hot” tumors are characterized by the abundant presence of CD4^+^ and CD8^+^ T cells surrounding tumor cells,^383^ emphasizing the critical need to effectively activate these anti-tumor T cells and produce a high concentration of T cell-attracting chemokines in the peritumoral lymph nodes.^384^ The positioning of T cells within tumors closely correlates with the expression of chemokines such as CCL2, CCL4, CCL5, CXCL9, and CXCL10. Stimulation of TNFSF14 initiates signaling via the lymphotoxin-β receptor, further enhancing an inflammatory microenvironment enriched with T cells. Notably, CD8^+^ effector T cells can enhance recruitment by releasing chemokines that attract similar cells already present in the tumor microenvironment.^385^ Tumor cell death can activate TLR3, prompting the production of type I interferons and subsequently CXCL10. Compared to “cold” tumors, “hot” tumors exhibit a higher mutational burden, leading to increased production of TAAs that enhance the immune system’s ability to recognize and eliminate tumor cells.^385^ In contrast to non-inflammatory tumors, inflammatory tumors demonstrate elevated expression of immunosuppressive genes like IDO, Foxp3, and PD-L1, activated as a response to positive anti-tumor immune reactions. Consequently, over time, T cells often face exhaustion, influenced by the chemokines present in the tumor microenvironment, the full extent of which remains incompletely understood.

Throughout various stages of tumor growth, the activation or inhibition of specific chemokine networks significantly impacts the immune milieu within the tumor microenvironment (TME), consequently influencing tumor biology and responses to therapy. However, the mere presence of chemokines cannot solely determine immune composition. The establishment of the tumor immune landscape is shaped by numerous factors, including genetic mutations within tumors, epigenetic changes, the host’s genetic makeup, the tissue origin of the tumor, microbial profile, and the intricate dynamics during cancer immune remodeling processes. Despite the complex interplay, evaluating cytokine and chemokine levels within tumors at distinct time points, along with the array of immune and non-immune cells present, is vital in specific contexts. This comprehensive evaluation assists in tailoring personalized therapeutic approaches for individual patients.

### The role of chemokines in modulating tumor immune cells

The initial recognition of the involvement of chemokines in tumor formation stemmed from investigations using viral and bacterial infection models, highlighting potential anti-tumor effects throughout tumor progression (Fig. 6). For instance, cDC1s mature through the processing of TAAs and response to inflammatory signals. This maturation process triggers the upregulation of CCR7 expression, vital for the migration of cDC1s from tumor sites to TDLNs. Deficiency in CCR7 within the cDC1 subset hinders anti-tumor T cell response, fostering tumor growth.^386^ Additionally, tumor-derived ligands binding to the liver X receptor (LXR) have been found to hinder the migration of DCs to TDLNs.^387^ Recent findings indicate that the CCR7-dependent migration of cDC2 from the TME to TDLNs is crucial for triggering the anti-tumor activity of CD4^+^ T cells. However, the recruitment of cDC2 is often obstructed by the suppressive activity of Treg cells.^388^ Tumor development induces changes in chemokine expression not only within the TME but also in surrounding tissues and lymphoid organs, which ultimately compromises immune cell activation. The TDLNs play a pivotal role in activating anti-tumor T cells, acting as a central hub for initiating this critical process. To effectively activate CD8^+^ T cells and CD4^+^ T cells targeting TAAs, a series of chemokine-mediated interactions with both mobile and stationary DCs in various lymph node regions is essential.^389^ Notably, factors originating from tumors have the ability to suppress the production of the stromal cell-secreted chemokine CCL21 in lymph nodes responsible for tumor drainage. The reduction in CCL21 expression, crucial for attracting T cells and DCs to lymph nodes, is apparent in TDLNs. This reduction in CCL21 levels is correlated with alterations in the distribution of immune cell populations, potentially leading to impaired T cell activation.^390^ Tumor-induced factors disrupt the normal process of hematopoiesis in the bone marrow, leading to the generation of granulocytic MDSCs.^391^ The regulation of immune cell development and maturation in the bone marrow is significantly influenced by the CXCR4-CXCL12 chemokine axis.^375^ Solid tumors boost the expression of CXCL12 in stromal cells, resulting in the accumulation of myeloid cells expressing CXCR4, ultimately causing disruptions in hematopoiesis.^392^ Further research is required for a comprehensive understanding of how tumor-derived elements impact the expression of chemokines in lymphatic organs like the spleen and thymus, and their role in the generation and activation of immune cells.

It is intriguing to observe that the same chemokine pathways often attract immune cells that both support tumor growth and inhibit tumor progression (Fig. 6). For example, chemokines like CXCR3 and its associated molecules CXCL9 and CXCL10, along with CCR5 and its ligand CCL5, can promote the recruitment of Treg cells and CD8^+^ T cells. As a result, the composition of immune cells may be shaped by the changing levels of chemokines in the TME and alterations in the expression of chemokine receptors on immune cells. There is variation among Treg cells in tumors, with different Treg subsets dominating different stages of tumor development.^393^ In the context of infection, the ability of Treg cells to suppress T cell responses depends on their expression of specific transcription factors and chemokine receptors that correspond to various T cell subsets.^394^ Treg cells expressing CXCR3 gather in the TME before Treg cells expressing CCR4 and CCR8.^395^ The emergence of CXCR3^+^ Th1 and CD8^+^ T cells has raised questions about the recruitment of CXCR3^+^ Treg cells. Additionally, more clarity is needed regarding the functional roles of different Treg cells subgroups in tumor progression. NK cells possess diverse functional characteristics, as seen in the unique expression patterns of CXCR3 and CX3CR1 in CD27^low^ and CD27^hi^ subsets. The expression of chemokines in the TME and the presence of corresponding chemokine receptors on NK cells may impact the recruitment efficiency of various NK cell subgroups into tumors and thus potentially influence the overall clinical outcome. The composition of immune cell subpopulations in the TME is significantly influenced by the presence of diverse immune cell subsets, adding complexity to TME interactions. For instance, the migration of conventional cDC1s to tumors relies on chemokines released by NK cells, such as XCL1 and CCL5.^45^ Notably, tumor-infiltrating cDC1s play a crucial role in producing CXCL9 and CXCL10, key chemokines that attract CD8^+^ effector T cells to the TME.^396,397^ Unlike other chemokine axes with overlapping functions, the XCL1-XCR1 axis uniquely recruits cDC1, indicating a potential therapeutic target to enhance cDC1 and T cell mobilization, and facilitate their interaction, thereby boosting T cell expansion and effector function acquisition.^398^ Moreover, an increased presence of MDSCs is associated with decreased CXCL11 expression levels and reduced infiltration of CD8^+^ T cells.^399^ Chemokine-mediated cellular feedback mechanisms may amplify subtle distinctions in early anti-tumor and pro-tumor responses as tumors progress.

The increasing recognition of the pivotal role of chemokines in modulating the properties and efficacy of immune cells within the TME is gaining momentum. An initial investigation reveals that the binary classification of cells as either pro-tumor or anti-tumor is inadequate to fully comprehend the complexity of cellular dynamics in the TME. For example, the phenotype of CD8^+^ T cells targeting tumors evolves throughout tumor progression. Distinct signatures of CD8^+^ T cells, such as TCF-1^+^ and CX3CR1^+^, are correlated with effective anti-tumor immune responses.^98,99,238^ Similarly, specific features of Treg cells, like CCR8^+^ Treg cells, may be linked to tumor advancement^400,401^ or response to PD-1 immunotherapy (IFNγ^+^ Treg cells).^402^ The heterogeneous and malleable characteristics of TAMs and TANs result in distinct pro-tumor (M1 TAMs and N2 TANs) and anti-tumor (M2 TAMs and N1 TANs) phenotypes. This diversity potentially encompasses a spectrum of intermediate cellular states.^403–405^

The maintenance of diverse cellular states within the TME and the potential for therapeutic manipulation of these states remain uncertain. The TME exhibits variations in oxygen levels, nutrient availability, and acidity, known factors that impact the characteristics and functions of immune cells. Therefore, the control of immune cell distribution in the TME through chemokines could have a profound influence on immune cell properties. Substantial alterations in chemokine receptor expression are commonly linked to the maturation and specialization of immune cells. For instance, as TCF-1^+^CD8^+^ T cells transition into effector CD8^+^ T cells and subsequently into exhausted T cells, there is a reduction in specific chemokine receptors such as CXCR5 and CXCR3, alongside an elevation in others like CXCR6 and CX3CR1.^225,406^ Moreover, in human tumors, TCF-1^+^CD8^+^ T cells are situated in a microenvironment rich in antigen-presenting cells.^407^ The upregulation of CXCR3 on these cells is crucial for their migration into the TME facilitated by the CXCL9 and CXCL10 ligands produced by cDC1s, promoting their revitalization and expansion in the TME.^408,409^ Exposure to IL-12 allows these cells to acquire effector functions.^410^ Additionally, the CXCR3 pathway and its ligands are postulated to play a role in directing the positioning of active immune cells in proximity to cancerous cells.^411^

The characteristics exhibited by TAMs and TANs are likely influenced by specific cues within the tumor microenvironment (TME) (Fig. 6). In the TME context, the interaction between CXCR4 and CXCL12, for example, facilitates the differentiation of recently arrived TAMs into perivascular TAMs.^412^ Resident TAMs play a crucial role in enhancing vascular permeability and assisting in the intravasation of tumor cells in the TME. Moreover, the inhibitory functions of TAMs in the TME can be modulated by the CCL5-CCR5 pathway. Disruption of CCR5 could induce a shift in TAM characteristics towards a pro-tumor profile, offering significant therapeutic benefits for individuals with colorectal cancer.^413^ Key chemokine signaling pathways, such as CCR2-CCL2 and CCR5-CCL5, play vital roles in mediating communication between tumor cells and TAMs, thus promoting tumor stemness, metastasis, and resistance to therapies.^414,415^ The immunosuppressive role of Treg cells in the TME is intricately linked to antigen recognition, which could trigger the recruitment of CCR8 and/or CCR4-dependent Treg cells towards DCs within the TME.^416^ The maintenance of a suppressive phenotype in Treg cells is supported by CCR8 expression. This is attributed to the enhancing effect of the CCL1-CCR8 signal on Foxp3 expression, a relationship associated with Treg cells phenotype stability.^402,417^ Additionally, CCR8^+^ Treg cells display elevated levels of suppressive markers, including CD25, CTLA4, CD39, TIGIT, PD1, ICOS, OX40, and Helios, compared to CCR8^-^ Treg cells.^418^ Conversely, CCR4 plays a crucial role in facilitating communication between Treg cells and DCs within lymph nodes, suggesting a potentially similar role within the TME.^419^ In the TME setting, activation of CCR6 signaling can promote the local expansion of Treg cells.^420^ Exploring the impact of chemokines on shaping cellular niches in the TME offers a promising avenue for future research. Although addressing chemokines and their receptors as therapeutic targets presents challenges, understanding their complexities may aid in identifying key cellular components involved in fostering or impeding tumor growth and immune responses. The burgeoning field of TME research is increasingly captivating, focusing on the intricate interplay between immune cells and chemokines, offering valuable therapeutic insights for modulating immune responses in cancer.

### The role of chemokines in cancer therapy

The essence of immunotherapy lies in triggering the patient’s immune system to combat tumor cells by rectifying effector cell dysfunctions and reducing the inhibitory immune cell population. Research indicates that the immune landscape of tumors can forecast the responsiveness to immunotherapy - favorable tumor environments (“hot” tumors) typically exhibit better responses to treatment, whereas cold tumors with minimal immune infiltration often present suboptimal responses. To bolster the efficacy of immunotherapy, there is a growing emphasis on integrating multiple cancer treatment modalities to overcome tumor drug resistance and enhance the reactivity of tumors less sensitive to traditional immunotherapy. A critical aspect of this approach involves regulating chemokine expression during tumor treatment, a factor crucial for both treatment effectiveness and patient tolerance.^421,422^

Immune checkpoint blockade (ICB) is a therapeutic strategy that enhances anti-tumor responses by freeing T cells from the constraints imposed by checkpoint molecules on their surface, thereby restoring T cell functionality. The success of checkpoint blockade therapy is closely linked to the intrinsic immune responses of patients.^79^ Patients with “hot” tumors, particularly those adept at attracting a substantial T cell influx, tend to derive greater benefits from these interventions.^62,423^ The effectiveness of cancer treatment hinges not only on the infiltration of T cells into the tumor microenvironment. Recent studies have highlighted the correlation between the efficacy of checkpoint inhibition therapy and the presence of specific T cell subpopulations expressing the TCF-1 transcription factor within tumor sites.^95,424^ Moreover, the chemokine receptor CXCR3 and its ligand CXCL9 play a pivotal role in enhancing the effectiveness of anti-PD-1 therapy in tumor studies.^425^ Inhibition of PD-1 leads to an upsurge in CXCL9 expression on conventional dendritic cells (cDC1), aiding in the targeted activation of CXCR3 expressing CD8^+^ T cells. This tactic helps prevent T cell exhaustion and initiates anti-tumor responses that effectively eliminate tumor cells. CXCL9 expression not only underpins clinical responses in the context of anti-PD-1 therapy but also significantly influences the success of TIM-3 blockade therapy in preclinical experiments. By stimulating CXCL9 production driven by cDC1, anti-TIM-3 therapy activates robust CD8^+^ T cell responses within the tumor, heightening sensitivity to paclitaxel chemotherapy.^426^ Therefore, the limited efficacy in highly inflamed tumor patients could be attributed to the reduced presence of TCF-1^+^ cells and/or impediments hampering their interaction with cDC1 in the tumor microenvironment.

Therapies utilizing CAR-T cells and ex vivo expanded autologous T cell infusions have demonstrated potential therapeutic benefits. However, a significant challenge confronting these treatments for solid tumors is the abnormal vascular structure and the suppressive tumor microenvironment, greatly impeding the infiltration of transplanted T cells into the tumor mass. Consequently, for patients with “cold” tumors and those undergoing TME phenotypic alterations, cell transfer therapy alone may not suffice for effective treatment. Research endeavors are underway to bolster the tumor penetration and functionality of T cells by investigating various chemokine systems in murine tumor models, including targeting CCR4,^427^ CXCR2,^428^ and CX3CR1.^429^ Despite these endeavors, there is an urgent need to translate preclinical findings into clinical practice to ascertain whether modulating chemokine systems can indeed enhance the efficacy of immune cell transfer therapy in cancer treatment.

Initially intended for tumor cell destruction, radiotherapy and chemotherapy are now acknowledged for their additional capacity to incite immune responses. By boosting the presentation of tumor-specific antigens on tumor cells, these treatments stimulate immune responses from anti-tumor T cells. Ongoing studies are exploring the synergistic effects of combining ICB with radiotherapy or chemotherapy. Hence, persistent efforts are being made to devise innovative radiotherapy and chemotherapy strategies to effectively augment T cell responses. Nevertheless, the collaborative interplay between these two treatment modalities may be hindered by CCR7^+^ cDC1 cells.^430,431^ Therefore, the efficacy of treatment protocols may be restricted if obstacles in recruiting cDC1 cells impede their accumulation in the tumor microenvironment, unless the treatment itself can enhance this process. Animal model research has revealed that anthracycline drugs can elevate the levels of CCL2 in the tumor microenvironment, thereby attracting antigen-presenting cells and facilitating robust immune responses against the tumor.^432^

Radiotherapy and chemotherapy have been demonstrated to elevate the release of chemokines in the tumor microenvironment. For example, radiotherapy application in a murine breast cancer model increases the expression of CXCL16, a key element facilitating the recruitment of CXCR6^+^CD8^+^ effector T cells to the tumor microenvironment.^433^ In murine melanoma models, radiotherapy induces the release of both type I and type II interferons, leading to heightened levels of CXCL9 or CXCL10, thereby promoting the infiltration of effector T cells expressing CXCR3.^434^ Chemotherapy induces the secretion of chemokines like CCL5, CXCL9, and CXCL10 in the tumor microenvironment, attracting CD4^+^ and CD8^+^ T cells to the tumor site. The expression of these chemokines correlates with the influx of CD4^+^ and CD8^+^ T cells, influencing tumor management and patient prognosis.^435^ Furthermore, in chemotherapy treatment regimens involving anthracyclines and conventional therapies, tumor cells exhibit increased levels of CXCL10 expression, a crucial factor in enhancing anti-tumor T cell responses.^385^

Tumor cells inherently resist treatment due to immune restrictions at therapy initiation. Additionally, tumors evolve continuously throughout treatment, incorporating various factors related to the local tumor microenvironment and the host. Treatment-induced alterations in chemokine expression may potentially contribute to resistance or recurrence in tumors. Elevated expression of chemokines such as CCL2 and CCL5 post-radiotherapy attracts immunosuppressive cell subgroups, including monocytes expressing CCR2 and CCR5, MDSCs, and Treg cells expressing CCR2, thereby assisting in cancer progression.^436–439^ Furthermore, therapy-triggered recruitment of CCR5-dependent macrophages contributes to tumor recurrence.^440^ Resistance to anti-BRAF inhibitor therapy in melanoma patients is linked to the CCL2-CCR2 pathway, where elevated CCL2 levels are associated with poor treatment response.^441^ Studies in a mouse melanoma model suggest a potential connection between anti-BRAF inhibitor resistance and CCR2^+^MDSC infiltration into the tumor microenvironment.^442^ Recent research identifies a novel chemotherapy resistance mechanism involving CCL2, where chemotherapy drugs prompt tumor cells to release extracellular vesicles, inducing CCL2 expression in vascular endothelial cells, attracting CCR2^+^Ly6C^+^ monocytes, and promoting tumor growth.^443^

Moreover, the CCR6-CCL20 axis may contribute to treatment resistance by recruiting CCR6^+^ Treg cells.^444^ Elevated levels of CCL20^+^ and CCR6^+^ Treg cells have been closely correlated with chemotherapy resistance in patients with colorectal cancer, and triple-negative breast cancer.^445,446^ In a mouse model of bladder cancer, resistance to PD-L1 immunotherapy is mainly linked to Gr1^+^ neutrophil infiltration into the tumor microenvironment, dependent on the CCL20 for immune cell attraction.^447^ This underscores the frequent occurrence of chemotherapy resistance in T cell therapy, the detailed mechanisms of which require further exploration. Considering the dual role of chemokines in modulating immune responses in cancer, their complex functions in treatment outcomes and resistance, and their impact on pro-cancer immune responses and immune suppression, chemokines play a critical role in delivering immune responses to the tumor microenvironment. Moreover, specific chemokine expression may influence treatment responses, potentially serving as biomarkers for predicting and monitoring treatment outcomes. Inducing or redirecting chemokine receptor expression in anti-tumor T cells represents a novel emerging therapeutic approach.

## Stroma: a microenvironment facilitating the “cold-to-hot” tumor transition

Unlike individual immune cells found within tumors, tumor stroma represents a structured that create a localized and crucial microenvironment for anti-tumor immune cells and humoral immune responses. The tumor stroma, comprising intricate molecules such as collagen and fibronectin, plays a vital role as a supportive tissue structure. The extracellular matrix environment surrounding tumor cells can significantly influence the response of immune cells to the tumor, at times creating a barrier that impedes immune cell function.^15,448,449^ The composition of the extracellular matrix is pivotal in the progression of cancer and shows promise as a biological indicator for the disease. A low tumor-stromal ratio (TSR) is strongly associated with a poorer prognosis in cancer patients, underscoring its importance as a critical prognostic factor for evaluating both prognosis and treatment outcomes in these individuals.^450,451^ Additionally, matrix components can exhibit growth-suppressive effects. Disruption of the matrix could lead to more aggressive tumor behavior and decreased long-term survival rates.^452^ Viewing the tumor matrix as an evolving entity, forthcoming research will primarily focus on elucidating how matrix elements accelerate the onset and progression of cancer, with the goal of establishing a theoretical foundation and preclinical evidence for stroma-based therapies.

### Composition of tumor stroma

**Fig. 7:**
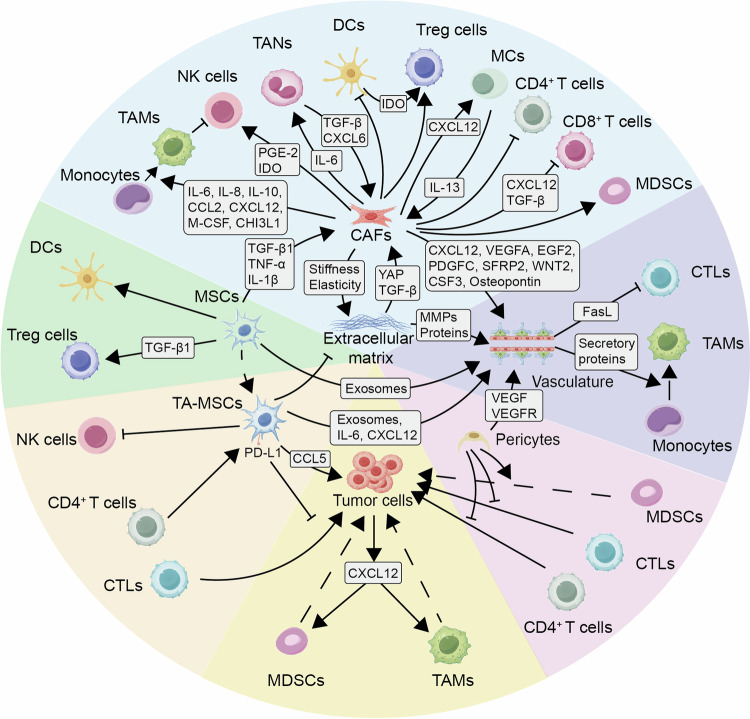
Illustrates the tumor stroma cells and non-cellular components influencing the tumor microenvironment. The tumor comprises cancer cells and an encompassing stroma, which is a key constituent of the tumor microenvironment (TME), displaying distinct characteristics specific to the tumor type. This encompasses the extracellular matrix, a unique cancer-related vasculature, and various cellular elements such as activated cancer-associated fibroblasts, mesenchymal stromal cells, and pericytes. The cellular and non-cellular components within the tumor stroma actively engage in interdependent interactions, playing crucial roles in a finely regulated dynamic process. This collaborative mechanism promotes the evolution, progression, dissemination, and resistance to treatment of cancer. Notably, these findings underscore the integration of stromal-based cancer therapies in discourse. A profound comprehension of the dynamic interplay between stroma and cancer cells is imperative for devising innovative therapeutic approaches. In this context, black arrows represent promotion, while black bars symbolize inhibition. This figure was created using Figdraw

Tumors are complex structures composed of a variety of cells, including tumor cells and various stromal components. The interactions between tumor cells and stromal elements play a crucial role in shaping the behavior of solid tumors. Tumor cells possess the ability to alter the surrounding stroma, creating a supportive microenvironment for their own growth. Interestingly, tumor cells can undergo transdifferentiation, transforming into cells resembling stromal cells through various signaling pathways.^453^ This transformation enhances tumor angiogenesis and drives cancer progression.^454^ The tumor stroma significantly impacts tumor formation, cancer progression, and resistance to therapy, thereby influencing various characteristics of cancer. Key stromal elements comprise the extracellular matrix, vascular system, activated cancer-associated fibroblasts, mesenchymal cells, and other cell components, which influence anti-tumor immune responses and ultimately determine the trajectory of tumor progression (Fig. 7).

### Stromal components in tumor microenvironment

#### Extracellular matrix and immune system

The elements within the tumor stroma cooperate to create an immunosuppressive environment, allowing tumor cells to evade immune detection and withstand attacks.^455^ Research has highlighted the significant role of the extracellular matrix (ECM) in regulating the development, movement, infiltration, and organization of immune cells in the tumor microenvironment (TME).^456,457^ The ECM not only provides crucial guidance for immune cell migration but also influences their behavior. Areas rich in loosely arranged fibronectin and collagen facilitate the movement and chemokine-dependent migration of T cells. Conversely, dense ECM regions impede T cell mobility, leading to reduced influx of CD8^+^ T cells.^458^ This indicates that increased ECM density may hinder T cell movement and positioning, impacting anti-tumor responses. Recent studies suggest that disrupting collagen stabilization can decrease stromal tissue amount and stiffness, enhancing the effectiveness of anti-PD-1 therapy and promoting more efficient T cell infiltration.^459^ Moreover, a rigid ECM can impair antigen-presenting cells’ (APCs) ability to present antigens effectively, potentially reducing IL-2 production critical for Th1 cell development and T cell expansion.^460^ Additionally, the matrix protein Tenascin-C can impede the cytoskeleton rearrangement necessary for T cell activation by interacting with α5β1 integrin on T cell surfaces.^461^ Furthermore, research has revealed that in breast cancer cases, areas with heightened collagen cross-linking levels often exhibit extensive macrophage infiltration. Eliminating these accumulated macrophages has been shown to reduce metastasis and stromal rigidity.^462^ The tumor stroma not only facilitates macrophage infiltration into tumor tissues but also drives their differentiation towards an M2 phenotype, enabling them to exert immunosuppressive functions.^463,464^

#### Tumor-associated vasculature and immune system

The tumor-related vascular system plays a crucial role in supplying the nutrients essential for tumor growth. Nevertheless, structural abnormalities within these vascular systems can hinder the effective delivery of drugs to the tumor site.^465^ It is important to highlight that the tumor vascular system creates an immunosuppressive environment by blocking the infiltration of T cells.^466^ Additionally, the irregular blood flow within the tumor causes hypoxia, which further encourages the development and function of M2 tumor-associated macrophages and Treg cells through various immunosuppressive mechanisms, ultimately aiding immune evasion by the tumor.^467^ Preclinical studies have shown that combining targeted vascular therapy with ICB may trigger potent anti-tumor responses.^468^ CAF mainly promotes neovascularization by releasing a variety of pro-angiogenic factors such as CXCL12, VEGFA, FGF2, PDGFC, WNT2, SFRP2, CSF3, and osteopontin.^469,470^ Moreover, by modulating the stiffness, elasticity, and interstitial fluid pressure of the extracellular matrix, CAFs can indirectly control tumor angiogenesis.^471,472^ Several studies have explored the role of tumor-associated mesenchymal stem cells (TA-MSCs) in angiogenesis, specifically focusing on chemotactic factors and growth factors that facilitate angiogenic processes, including VEGF, IL-6, and the CXCL12/CXCR4 signaling pathway.^473–475^ Research indicates that TA-MSCs can enhance angiogenesis through their ability to differentiate into endothelial cells or attract endothelial progenitor cells.^476^ Furthermore, further validation is needed to determine if similar outcomes can be observed in the tumor microenvironment, where mesenchymal stem cells (MSCs) can release exosomes to transport miRNAs to endothelial cells and promote in vitro angiogenesis.^477^

#### Tumor endothelial cells and immune system

Tumor endothelial cells (TECs) play a key role in shielding tumor cells from immune attacks launched by the host.^478^ The proteins secreted by TECs trigger the polarization of macrophages towards the M2 phenotype by activating the PI3K/AKT/mTOR signaling pathway.^479^ Notably, TECs possess the ability to upregulate the expression of Fas ligand under the influence of various stimuli, such as a combination of IL-10, prostaglandin E2 (PGE2), and VEGF-A. This modification enables TECs to selectively target effector CD8^+^ T cells over Treg cells, facilitating tumor cell evasion.^114^ In hepatocellular carcinoma (HCC), TECs can influence the levels of CD8^+^ T cells by modulating the expression of glycoprotein non-metastatic melanoma protein B (GPNMB), resulting in their accumulation or depletion.^480^ Additionally, TECs frequently exhibit increased expression of PD-L1, enabling them to interact with PD-1 on activated lymphocytes, therefore obstructing the immune response.^481,482^ Activated cancer-associated fibroblasts (CAFs) play pivotal roles in immune regulation by remodeling the extracellular matrix to hinder immune responses, regulating the anti-tumor functions of immune cells within tumors, and boosting the expression of immune checkpoint molecules.^483–485^ Regarding innate immune responses, tumor-associated macrophages (TAMs) are commonly found near areas rich in CAFs, engaging in various interactions with them. CAFs actively participate in attracting monocytes to the tumor site, where they differentiate into M2 macrophage subpopulations linked with the tumor.^486–488^ Specifically, CAFs play a critical role in recruiting monocytes and promoting their transition to M2 macrophages by releasing IL-8, thereby impeding the activity of NK cells effectively.^489^ CAFs release a spectrum of factors, such as CCL2, CXCL12, IL-6, IL-10, glycoprotein CHI3L1, and macrophage colony-stimulating factor, known for their roles in cancer progression and influence on the tumor microenvironment. The importance of these factors in facilitating monocyte recruitment to tumor sites and supporting their transformation into M2 macrophages is significant.^490–496^ Notably, TAMs play a crucial role in regulating the activation of CAFs through the secretion of CXCL12 and IL-6, initiating a beneficial feedback loop that promotes cancer progression.^496^ Neutrophils, similar to macrophages, are broadly categorized into two polarized subsets based on their phenotypic variances: the anti-tumor N1 neutrophils and the pro-tumor N2 neutrophils.^497^

Interleukin-6 (IL-6), derived from cancer-associated fibroblasts (CAFs), plays a crucial role in initiating the activation of the STAT3 pathway in tumor-infiltrating neutrophils, known as TANs. This activation supports the survival and function of TANs while simultaneously inhibiting the immune response of T cells via the PD-1/PD-L1 pathway.^498^ Furthermore, the upregulation of CXCL6 and TGF-β in cancer cells is triggered by cardiotrophin-like cytokine factor 1 (CLCF1) from CAFs, thereby promoting N2 neutrophil polarization.^499^ MDSCs are a cellular population originating from the bone marrow, consisting of immature marrow cells and progenitors. Their role in tumor progression involves immune suppression by modulating both innate and adaptive immune responses.^500,501^ CAFs induce Treg cells to suppress their anti-tumor response. Specifically, through the release of various substances such as chemoattractants (CCL2, CXCL1, CXCL2, and CXCL12) and cytokines (IL-6 and TGF-β), CAFs facilitate the generation and migration of MDSCs.^502^

Dendritic cells (DCs) are a crucial subset of immune cells responsible for presenting antigens and playing a vital role in the body’s defense against cancer. However, their function can be influenced by cancer-associated fibroblasts (CAFs), leading to immune evasion by tumors. The interaction with CAFs can result in impaired maturation and hindered antigen presentation in DCs.^503^ Studies have demonstrated that CAFs attract DCs and boost their production of indoleamine 2,3-dioxygenase (IDO), thereby inhibiting T cell proliferation through an IL-6-STAT3-mediated mechanism, ultimately contributing to an increased generation of Treg cells.^504^ Additionally, CAFs enhance the proliferation and migration of mast cells (MCs) through the activation of the CXCL12/CXCR4 pathway, thereby promoting the tumor-supporting functions of MCs.^505^ Stellate cells, acting as precursors to CAFs, play a significant role in creating a fibrotic tumor microenvironment by stimulating MCs to secrete IL-13 and histidine decarboxylase, which compromises anti-tumor immune responses.^506^ Moreover, histidine decarboxylase released by MCs facilitates early malignant morphological changes in prostate epithelial cells initiated by CAFs.^507^ CAFs can induce dysfunction in NK cells by releasing prostaglandin E2 (PGE2) and IDO, altering the function and phenotype of NK cells.^508,509^ The impact of CAFs on adaptive immunity primarily involves modulating T lymphocyte activity. Antigen cross-presentation facilitated by CAFs could negatively affect the function and survival of T cells.^510^ Mechanistically, programmed cell death ligand 2 (PD-L2) expressed by CAFs triggers immune tolerance or apoptosis in T cells by interacting with PD-1. Furthermore, CAFs secrete FAS ligand (FASL) to induce apoptosis in CD8^+^ T cells expressing the FAS receptor.^511^ The inhibitory effects of TGF-β on the infiltration of CD8^+^ T cells compromise the efficacy of anti-tumor immunity.^69,70^ CAFs release CXCL12, which impedes the infiltration of CD8^+^ T cells and contributes to the resistance to T cell checkpoint blockade therapy.^512,513^ By regulating immune checkpoint molecules such as PD-L1, PD-L2, B7-H3, and B7-H4, CAFs influence T cell activity, particularly interacting with PD-1 receptors on T cells.^514,515^ CAFs also impact T helper cell subgroups, specifically inhibiting anti-tumor responses by influencing the Th2 cell subgroup and the conversion of Treg cells.^483^

#### Cancer-associated fibroblasts and immune system

Activated cancer-associated fibroblasts (CAFs) have diverse effects on immune responses. They create a physical barrier that impedes immune cell penetration into tumors through modifications to the ECM. Additionally, CAFs modulate the activity of anti-tumor immune cells and enhance the expression of immune checkpoint proteins, thus affecting immune surveillance within the TME.^484^ Tumor-associated macrophages (TAMs), a crucial component of the innate immune response, are abundant in areas rich in CAFs, leading to complex interactions between these cellular entities and underscoring the importance of TAMs in the TME.^488^ CAFs actively recruit monocytes to the TME and promote their differentiation into M2 macrophages, thereby facilitating tumor progression. Specifically, CAFs induce monocyte migration and polarization towards M2 macrophages by releasing IL-8, resulting in the suppression of NK cell activity.^489^ Furthermore, the secretion of various cytokines by CAFs attracts monocytes to the TME, promoting their differentiation into M2 macrophages.^491^ Tumor progression is further supported by tumor-associated macrophages, which produce CXCL12 and IL-6 to enhance CAF function. Neutrophils can differentiate into two distinct subtypes: anti-cancer N1-type neutrophils and pro-cancer N2 neutrophils.^496^ CAF-produced IL-6 activates tumor-associated neutrophils (TANs) and inhibits T cells through the PD-1/PD-L1 signaling pathway.^498^ Additionally, CAFs have been found to elevate CXCL6 and TGF-β levels in tumor cells, promoting the activation of N2 neutrophils.^499^ When influenced by CAFs, MDSCs hinder the anti-tumor activity of effector T cells by facilitating their recruitment and infiltration through the release of factors such as CCL2, CXCL1, CXCL2, CXCL12, IL-6, and TGF-β.^502^ CAFs also impact DCs, leading to immune evasion in tumors by suppressing antigen presentation and impeding DC maturation. Studies indicate that CAFs recruit DCs and induce the expression of IDO, which suppresses T cell proliferation and promotes Treg cells generation via the IL-6-STAT3 signaling pathway.^504^ Furthermore, CAFs activate the CXCL12/CXCR4 pathway, promoting tumor cell proliferation and migration.^505^ Stimulation of precursor mast cells by IL-13 and the secretion of tryptase contribute to the establishment of a fibrotic TME that facilitates immune evasion.^506^ Conversely, tryptase released by mast cells triggers early malignant transformation of prostate epithelial cells by activating CAFs.^507^ CAFs also impair NK cell function by modulating their phenotype and function through the release of PGE2 and IDO.^509^

Cancer-associated fibroblasts (CAFs) play a crucial role in modulating the adaptive immune system, primarily by regulating the activity of CD8^+^ T cells. Their key function involves antigen cross-presentation, which can detrimentally affect T cell function and viability.^510^ For example, CAFs that express PD-L2 have the capability to induce functional exhaustion or apoptosis in T cells by interacting with PD-1. Furthermore, CAFs secrete FASL, which initiates apoptosis in CD8^+^ T cells that express FAS.^511^ The presence of TGF-β is recognized to hinder the anti-tumor immune responses by impeding the infiltration of CD8^+^ T cells into the tumor microenvironment.^70^ Moreover, the essential role of CXCL12 produced by CAFs is to disrupt the communication between CD8^+^ T cells and tumor cells, thereby reducing the efficacy of ICB therapy.^513^ CAFs regulate T cell functionality by modulating the levels of immune checkpoint proteins like PD-L1, PD-L2, B7-H3, and B7-H4. Additionally, CAFs influence specific subsets of T helper cells, such as the Th2 cell population, and facilitate the conversion of Treg cells, consequently suppressing anti-tumor immune responses.^484^

#### Tumor-associated mesenchymal stromal and immune system

Tumor-associated mesenchymal stem cells (TA-MSCs) primarily induce immune suppression by inhibiting surface receptor activation on effector cells and disrupting the function of antigen-presenting cells, ultimately resulting in immune system suppression.^516^ Through the utilization of IL-10, TA-MSCs decrease the presence of HLA-I molecules on the surface of tumor cells, hindering the ability of CTLs to efficiently detect and eliminate the tumor cells.^517^ Moreover, TA-MSCs boost the proliferative capacity of FoxP3^+^ Treg cells by upregulating levels of TGF-β1 while concurrently decreasing pro-inflammatory Th1 cell cytokines and increasing anti-inflammatory Th2 cell cytokines. This dual effect allows tumor cells to evade immune surveillance and impairs the immune system’s ability to mount an effective response against tumor cells.^518^ MSCs disrupt effective immune responses by impeding DCs maturation, resulting in diminished CD83 expression on the surface of DCs.^519^ Additionally, TA-MSCs typically exert inhibitory effects on mononuclear cell proliferation and suppress NK cell activity.^520^ The crosstalk among TA-MSCs, tumor cells, and macrophages involves various chemokines, with the invasion and metastasis of MDA-MB-231 cells being significantly facilitated through hypoxia-inducible factor (HIF) signaling.^521^ Notably, TA-MSCs secrete CXCL10, which binds to CXCR3 receptors on cancer cells, leading to the release of CXCL16 by cancer cells, which in turn binds to CXCR6 receptors on TA-MSCs, promoting the migration of TA-MSCs towards tumor sites. Furthermore, TA-MSCs release CCL5, which can bind to CCR5 receptors on breast cancer cells, enhancing CXCL12 expression within stimulated cancer cells and facilitating the migration and infiltration of TAMs and MDSCs.^521^ The interaction between TA-MSCs and immune cells, particularly CD4^+^ T cells, is a significant aspect that warrants attention.^522^ TA-MSCs demonstrate adaptability and responsiveness to signals from CD4^+^ T cells, which, in turn, promote tumor progression. Upon activation by CD4^+^ T cells, TA-MSCs undergo notable changes in their immune profile, resulting in increased PD-L1 expression mediated by the STAT3 pathway. Consequently, this triggers the internal PD-1/mTOR signaling cascade within cancer cells, furthering the progression of gastric cancer.^522^

#### Pericytes and immune system

Pericytes demonstrate their immunoregulatory capacity by secreting a diverse array of molecules, such as nitric oxide, CXCL12, IL-6, IL-33, PGE2, and TGF-β.^523^ The function of cytotoxic lymphocytes is impacted by the buildup of pericytes, which impede the alloreactive and mitogen-stimulated T cell reactions in experimental settings.^524^ Additionally, a novel finding indicates that pericytes derived from tumors possess inhibitory effects on the growth and stimulation of CD4^+^ T cells. This leads to compromised functionality of CD4^+^ T cells in antigen generation, potentially resulting in unresponsiveness. Importantly, this effect is mediated by IL-6, hindering the efficacy of anti-tumor immune responses and providing a shield for tumor cells against host immune assaults.^525^ Pericytes have a crucial function in attracting MDSCs to the stroma, forming an immunosuppressive TME that facilitates tumor progression.^526^

### Targeting therapy based the tumor stroma

When considering targeted therapy based on the tumor stroma, the focus shifts towards non-malignant cellular components, rather than solely on tumor cells as in conventional approaches. The advancements in precision medicine have accelerated the clinical applications of molecular targeted therapy. The occurrence and metastasis of cancer result in the development of a complex stromal environment within the host tissues, directly driving cancer progression. Therefore, the tumor stroma is seen as a crucial target for advancing therapeutic approaches, providing the opportunity to improve current treatment options and customize treatments to individual needs. Consequently, the tumor stroma is deemed a critical focal point for designing successful therapeutic strategies to enhance current treatment modalities and achieve personalized treatment goals. Strategies that target the tumor stroma involve directly influencing the cells or non-cellular elements within the stroma, as well as implementing novel approaches to remodel or normalize the tumor stroma to hinder or reverse tumor progression. By summarizing the latest research advancements on stromal components, this article highlights their potential therapeutic value and advocates for the translation of laboratory research into clinical applications.

#### Targeting extracellular matrix of tumor stroma

In comparison to normal ECM, tumor stroma exhibits increased richness, density, and stiffness, undergoing various alterations such as deposition, degradation, and post-translational modifications.^527^ One effective strategy involves targeting lysyl oxidase (LOX), an enzyme commonly upregulated in various cancers that facilitates collagen cross-linking. Inhibiting LOX can decrease stromal density and boost the effectiveness of anticancer therapies. Several clinical trials have been conducted to assess the efficacy and safety of utilizing LOXL2 inhibitors (e.g., simtuzumab) in combination with other treatments for patients with pancreatic and colorectal cancers. However, the results have shown discrepancies.^528^ Another approach focuses on reducing the accumulation of hyaluronic acid (HA) in cancer, which mechanically increases stromal viscosity.^529^ Clinical trials testing drugs like PEGPH20, which target and inhibit HA, have shown diverse outcomes in various cancer treatments.^530^ Connective tissue growth factor (CTGF) promotes stromal deposition in cancer.^531^ Anti-CTGF therapy exhibits potential in reducing stromal accumulation in a pancreatic cancer mouse model. Ongoing clinical trials are investigating the impact of CTGF-targeting drugs like pamrevlumab on patients with pancreatic cancer.^532^ Integrins play a crucial role as mechanical signaling molecules in the stroma and have emerged as promising targets for slowing tumor progression. Therapeutic potential of drugs targeting integrins is being evaluated in clinical trials. Transforming growth factor-beta (TGF-β) plays a crucial role in inhibiting collagen synthesis and the subsequent stromal deposition in cancer.^533^ Numerous drugs targeting TGF-β are actively being studied to enhance anticancer effects.^534^ Targeting FAK, a downstream effector of integrins, through FAK inhibitors has shown anti-tumor activity.^535^ Cancer vaccines are increasingly utilized as a promising treatment strategy against solid tumors, incorporating several extracellular matrix components as antigens in their design.^536^ Notably, an additional region, extra domain A (ED-A), selectively spliced in fibronectin, has been identified as a potential therapeutic target in reshaping the tumor stroma.^537^

#### Targeting tumor-sssociated vasculature and endothelial cells

Vascular endothelial growth factor (VEGF) and its receptors (VEGFR) constitute a frequently targeted pathway in anti-angiogenic therapy. For instance, bevacizumab disrupts the interaction between VEGF-A and VEGFR-1 and -2.^538^ Clinical studies have confirmed the long-term safety and benefits of bevacizumab in solid tumor patients. However, the results of its use in combination with chemotherapy have been inconsistent, highlighting the need to understand specific treatment regimens and conditions to achieve optimal clinical responses. Combining bevacizumab with targeted therapies, such as erlotinib, has shown synergistic effects and improved progression-free survival outcomes for various cancer types.^539^ Ongoing clinical trials are investigating tyrosine kinase inhibitors (TKIs) targeting alternative angiogenic signaling pathways like fibroblast growth factor (FGF)/FGFR and platelet-derived growth factor (PDGF)/PDGFR.^540^ Approved TKIs such as sorafenib, sunitinib, and pazopanib have proven clinical value in various cancers, with special attention being drawn to sorafenib in hepatocellular carcinoma.^541^ Furthermore, dual inhibition strategies targeting VEGF and angiopoietin-2 (ANG2) have shown to extend normalization periods and potential immunotherapeutic benefits.^542^ Immunotherapy is increasingly vital in cancer treatment, with studies supporting clinical benefits from combining PD-1 inhibitors with bevacizumab.^543^ Despite promising results, anti-angiogenic therapies in clinical settings typically offer only partial benefits and may lead to relapse. This section emphasizes the ongoing research and assessment of various anti-angiogenic treatments and combination approaches aimed at enhancing outcomes for cancer patients.

In cancer treatment strategies, the significance of vascular normalization lies in circumventing the immunosuppressive environment and fostering disease progression. A promising approach to enhance tumor oxygenation and drug delivery efficiency is the judicious use of anti-angiogenic therapy to maintain the balance between pro-angiogenic and anti-angiogenic signals.^544^ This underscores the critical aspects of drug dosage and timing, known as the “normalization window,” used to effectively leverage anti-angiogenic drugs for the desired vascular normalization effects. Overutilization of anti-VEGF drugs can result in the emergence of a hypoxic and immune-suppressive tumor microenvironment, emphasizing the importance of selecting appropriate drug dosages in cancer treatment.^545^ Research indicates that lower doses of bevacizumab, as opposed to higher doses, may enhance drug delivery efficiency and vessel coverage in colorectal cancer patients, highlighting drug dosage as a pivotal consideration in cancer treatment.^546^ The combination therapy of immunotherapy and chemotherapy demonstrates a synergistic effect, especially within the “normalization window”.^547^ The optimum dosage range of potent anti-VEGF drugs targeting this window is restricted and may vary across different cancer types, presenting a significant challenge in maximizing the benefits of anti-angiogenic therapy in cancer patients.^548^

#### Targeting cancer associated fibroblasts

Targeted therapy against cancer-associated fibroblasts (CAFs) has emerged as an attractive strategy in cancer intervention, given the association of activated CAFs with poor patient prognosis, treatment resistance, and disease recurrence.^549^ Various approaches are being explored, such as the selective removal of surface markers on CAFs to address their role in promoting tumor progression.^550^ This section emphasizes the cautious and personalized integration of anti-angiogenic therapy with immunotherapy or strategies targeting CAFs to enhance anti-tumor immune responses while minimizing toxic side effects, offering rich potential for improving cancer treatment outcomes.^551^ A unique DNA vaccine directed at the FAP antigen has been formulated with the purpose of triggering the activation of CD8^+^ T cells. This vaccine aims to eradicate CAFs, inhibit the proliferation of both primary and metastatic tumors, improve the absorption of chemotherapy agents, and prolong the survival of mice harboring tumors.^552^ The co-administration of the FAP-DNA vaccine with other DNA vaccines specific to tumors has demonstrated an enhancement in the influx of CD8^+^ T cells and a reduction in the presence of macrophages within the tumor microenvironment (TME).^553^ Moreover, utilizing CAR-T cells targeting FAP has shown effectiveness in inhibiting the function of FAP-producing CAFs, leading to a reduction in tumor growth and proliferation following subcutaneous implantation.^554^ A bispecific FAP-CD40 antibody has been specifically engineered to activate CD40 in the presence of FAP. This targeted approach aims to primarily trigger immune activation within tumors while demonstrating favorable tolerability.^555^ Additionally, phototherapy targeting FAP has shown promising results in sensitizing FAP-enriched tumors to chemotherapy and inducing tumor shrinkage. Several other drugs or inhibitors targeting FAP are undergoing validation in preclinical studies, with some advancing to clinical trials.^556^ In addition to FAP, a noteworthy increase in the expression of α-smooth muscle actin (α-SMA) functions as a distinctive indicator for CAFs. Heightened α-SMA levels have been recognized as a novel indicator for initial resistance to trastuzumab in HER2-positive breast cancer.^557^ Recent research indicates that enhancing drug delivery efficiency by targeting α-SMA with paclitaxel bound to nanoparticles can reduce cancer metastasis through improved vascular perfusion.^558^ In a murine model of PDAC, the reduction of α-SMA^+^ fibroblasts resulted in various outcomes: inhibiting blood vessel formation, triggering a dense stromal tumor characteristic, facilitating the migration of CD3^+^Foxp3^+^ Treg cells into the TME, and ultimately enhancing tumor aggressiveness while reducing survival rates in the animal model. Research suggests that the restoration of tumor-promoting fibroblasts, known as CAFs, can be achieved by employing ligands that interact with the vitamin D receptor (VDR).^559^ Notably, calcipotriol has improved chemotherapy outcomes in various mouse tumor models by hindering interactions within the tumor stroma and tumor proliferation.^560^ The high expression of TGF-β in CAF activation and cancer progression is crucial. LY2157299 is an oral small molecule inhibitor that can prevent CAF activation and immunosuppression.^561^ Another drug, minnelide, is also being studied in clinical trials to block TGF-β signaling.^562^ In conclusion, although CAFs are considered an attractive target in cancer therapy, clinical outcomes of CAF-targeted therapies remain suboptimal and warrant further in-depth research. Challenges persist, including the heterogeneity and plasticity of CAFs, as well as understanding their tumor-promoting and inhibitory functions. Accelerating research efforts to overcome these issues is essential.

#### Targeting tumor-associated mesenchymal stromal cells

Tumor-associated mesenchymal stem cells (TA-MSCs) represent a promising avenue for the development of effective anti-cancer therapies. These cells elevate the expression of PD-L1 through the CXCL12/CXCR4 signaling pathway to orchestrate tumor immune suppression.^563^ One approach involves the use of ola-PEG to counteract the effects of CXCL12,^564^ while another technique focuses on targeting CXCR4 with specific antagonists like AMD3100.^565^ Recent research indicates that TA-MSCs mainly exert their immunosuppressive effects by releasing the immunomodulatory factor IDO.^566^ Inhibiting IDO activity hinders the movement of CD8^+^ T cells and B cells, resulting in immune evasion and increased resistance to anti-CTLA-4 therapy.^567^ Ongoing clinical trials are investigating the effectiveness of IDO blockers such as navoximod in the field of oncology treatment. Additionally, emerging therapeutic approaches leverage the intrinsic tumor-targeting capacity of MSCs as carriers for anti-cancer drugs.^568^ Interferons with anti-tumor properties have been successfully utilized in various cancer types. Transgenic MSCs, transfected with interferon-alpha or interferon-beta, are engineered to exhibit different levels of anti-tumor efficacy.^569^ This strategy aims to combat tumors by enhancing apoptosis and enhancing the functions of NK cells and CD8^+^ T cells, thereby reinforcing the host’s immune response against cancer.^570^

#### Targeting pericytes in cancer therapy

Advancements in cancer therapy targeting stromal cells have progressed smoothly, showing benefits in preclinical studies. Strategies directed at stromal cells, such as using Nilotinib to enhance blood-brain barrier permeability and augment chemotherapy effectiveness to prolong animal survival, hold promise.^571^ Research utilizing the specific tyrosine kinase inhibitor imatinib targeting stromal cells has demonstrated a reduction in PDGFRβ^+^ stromal cells in mouse and human tumor models, slowing lymphoma growth.^572^ Developing novel therapeutics targeting stromal cells and selecting appropriate combination therapies may enhance cancer control and alleviate symptoms.

The initiation, progression, and metastasis of cancer often involve dynamic changes in the host tissue, creating a complex stromal environment that supports infiltration and dissemination of tumor cells. The tumor stroma plays a vital role in almost all malignant diseases, establishing it as a rational target for therapeutic interventions. Various stromal-targeting therapies have been developed and studied to reduce or eliminate the stroma supporting tumor growth. However, stromal-targeting strategies primarily result in tumor growth deceleration or modestly prolonging patient survival, with few leading to complete cure. Challenges persist in effectively targeting pro-tumorigenic stromal populations without harming healthy tissues, including identifying specific stromal cell subtypes lacking surface markers like CAFs and TA-MSCs, as well as understanding the mechanical and functional roles of stromal components in cancer. Understanding the intricate interactions between stromal cells and cancer cells, especially in cancers characterized by dense stroma like PDAC, is crucial in developing new treatment strategies. Despite numerous challenges, stroma-targeted therapies as emerging cancer treatment strategies hold potential to reshape the treatment landscape and provide greater clinical benefits for cancer patients.

## The immunological reservoir of “hot” tumors: third lymphoid structures

The third lymphoid structure (TLS), also known as ectopic or tertiary lymphoid organs, is a site where immune cells gather in non-lymphoid tissues.^573^ Studies have associated TLS with ICB, which can provoke more favorable immune responses and improve survival rates in cancer patients.^573^ Recent research has emphasized a strong link between TLS and cancer, showing that patients with TLS tend to have better prognoses and lower recurrence risks.^574^ Investigations into bladder cancer and cholangiocarcinoma have demonstrated a connection between TLS and positive prognoses, likely due to the heightened anti-tumor immune responses occurring within TLS.^575–577^ As a result, current studies are delving into the immunological mechanisms and clinical importance of TLS in malignant tumors.

### Cellular components of TLS

Tertiary lymphoid structures (TLS) are formed in response to persistent inflammatory stimuli, such as infections, transplant rejections, autoimmune diseases, and malignant tumor tissues.^578^ These structures structurally resemble secondary lymphoid organs (SLO) and comprise B cell follicles enclosing follicular dendritic cells (FDCs). Within the T cell zone, high endothelial venules (HEVs) aggregate together, accompanied by fibroblastic reticular cells (FRCs) present within TLS. In contrast to secondary lymphoid organs (SLOs), TLS lack capsules or afferent/efferent lymphatic vessels.^579^ The predominant T cell types in TLS include CD4^+^ follicular helper T cells (TFH), CD8^+^ T cells, CD4^+^ Th1 cells, and CD4^+^ Treg cells.^573^ Notably, stromal cell-like T cells are widely distributed throughout TLS, suggesting a significant role in their differentiation, support, and protection.^580^ Dendritic cells (DCs) are situated within the T cell zone, and their abundance correlates with the degree of Th and CD8^+^ T cell infiltration, thereby promoting B cell expansion and antibody production.^581^ The stromal cells in TLS consist of fibroblasts, FDCs, and FRCs. FDCs facilitate communication between antigen-presenting cells and B cells in the primary lymphoid follicles, while FRCs, located in the T cell zone, provide structural support for TLS.^582^

### Immune properties of TLS in tumors

Cancer is commonly perceived as a continuous and dynamic inflammatory process involving tissue damage, cytokine production, chemotaxis of immune cells, cell proliferation in response to growth factors, and stimulation of tissue repair.^583^ Tertiary lymphoid structures (TLS) within tumors are recognized as a vital immunogenic trait especially evident in highly immunogenic tumors.^584^ The immune response within TLS is governed by tumor genetic characteristics, encompassing neoantigens, tumor mutational burden (TMB), expression of immune checkpoints, and immune cell infiltration.^585,586^ Cellular therapies commonly address neoantigens, which encompass both tumor-associated and tumor-specific antigens.^587^ Additionally, immune checkpoint expression levels within TLS can trigger responses that facilitate tumor progression, such as activation of indoleamine 2,3-dioxygenase 1 (IDO1),^588^ upregulation of PD-L1,^589^ recruitment of MDSCs,^438^ and induction of T-cell exhaustion.^590^

The maturation and localization of TLS are influenced by various factors, shaping their functionality within tumors.^591^ TLS functionality is significantly impacted by cellular diversity at different developmental stages. Primary follicular TLS structures, rich in B cells, exhibit a potential target for antibody therapies. In contrast, secondary follicular TLS structures comprise mature T and B cells, rendering them more responsive to T-cell therapies and immune checkpoint blockade.^592^ Mature TLS, in opposition to immature TLS, demonstrate heightened immune activity and prognostic significance.^593^ The importance of understanding the influence of tumor immune characteristics on TLS maturation is highlighted by the observed disparities between mature and immature TLS across various malignancies. Predominantly located in the peritumoral area, mature TLS display T-cell activation signals, plasma cell expansion, and CXCL13 expression.^594^ In melanoma, IKZF1 has been recognized as a key player in the formation of immature TLS, significantly impeding TLS maturation without affecting melanoma cell proliferation.^595^ Notably, CD8^+^ T cells exhibit distinct functionalities based on their origin from either the inflammatory environment or the tumor itself. Tumor-infiltrating lymphocytes originating from inflammation tend to create a conducive milieu for tumor cell proliferation, while those arising from the tumor site typically support antitumor immunity.^596^ Hence, further exploration of the spatial distribution of immune cells within TLS is warranted. The establishment and efficacy of TLS within tumors and adjacent tissues are substantially influenced by the tumor’s genetic attributes and the nature of the inflammatory response. The composition, localization, and maturation of TLS have been correlated with their immune response.

### Blocking immune checkpoints of TLS

Unprecedented responses to ICB therapy have been observed across various types of cancers.^579^ However, not all patients demonstrate favorable responses, emphasizing the pressing need to identify precise biomarkers for response prediction. The tumor mutation burden is recognized as a potential predictive indicator linked to treatment response, although current biomarkers lack the ability to consistently forecast responses to immunotherapy.^582^ Notably, the presence of pre-existing anti-cancer T cells and the level of PD-L1 expression within the tumor microenvironment are pivotal factors in predicting the efficacy of anti-PD-1 therapy for patients with melanoma and non-small cell lung cancer (NSCLC).^581,597^ In the context of ICB therapy, PD-L1 plays a critical role in revitalizing CD8^+^ T cells by inhibiting their activity, thereby transforming them into potent cytotoxic cells that target tumor cells. This mechanism involves the expression of PD-L1 on both tumor cells and myeloid cells.

Tertiary lymphoid structures (TLS) serve as crucial sites for generating memory effector cells responsible for managing tumor recurrence, presenting a unique opportunity to inform forthcoming clinical investigations in the rapidly evolving field of immuno-oncology. A study conducted in patients with non-small cell lung cancer demonstrated that those who underwent neoadjuvant therapy with anti-PD-1 agents showed an increased formation of TLS within tumors after surgical intervention. Additionally, the identification of PD-1^+^CD8^+^ T cells within TLS prior to treatment initiation had the potential to predict the efficacy of anti-PD-1 therapy.^598^ Furthermore, the induction of TLS in regressing lesions is associated with the response of patients with cervical intraepithelial neoplasia to human papillomavirus (HPV) vaccines, the primary causal agent of cervical cancer.^599^ TLS not only serve as markers of therapeutic immune responses in cancer but also contribute to enhancing anti-tumor immune reactions. The induction of TLS in tumors is believed to aid in recruiting lymphocytes and controlling tumor progression, potentially broadening the application of adjuvant immune therapy in both immunologically “cold” and “hot” tumors.

While TLS are observable in diverse malignancies, further research is warranted to clearly characterize, distinguish, and quantify TLS, as well as to conduct large-scale, multicenter studies. Moreover, the integration of single-cell sequencing and spatial transcriptomics is essential for exploring the relationship between cancer driver genes and TLS formation. Improving the understanding of TLS functionality and its roles across different tumor types amidst the complexity of cancer can hasten the development of enhanced therapeutic strategies. This, in turn, can boost tumor responsiveness to immunotherapy, leading to improved treatment outcomes and enhanced survival rates.

## Microbiota: regulating the host microbiome of “hot” tumors

The microbial composition within tumors functions as a pivotal component of the tumor microenvironment, representing a population of “permanent residents” rather than transient occupants. In cancer, interactions between the immune system and the microbiota primarily occur at mucosal surfaces, influencing the entire body through microbial metabolites and outer membrane vesicles. Alternatively, these interactions can transpire locally within lymphoid organs or the tumor microenvironment.^600^ Microbial signals stemming from both local and distant sources have the potential to modulate innate and adaptive immune responses, leading to systemic or tumor microenvironment-specific immune regulation and immunosurveillance.^601^ Metabolites generated by microbes can systemically circulate and reach distant tumor sites, initiating innate immune responses that either suppress or boost tumor progression.^20^ Notably, various strategies targeting the microbiota have been extensively utilized in the field of oncology; however, determining the optimal approach for targeting both tumors and the microbiota remains uncertain.^602,603^ This review focuses on exploring the intricate relationship between the microbiota, cancer immune responses, and cancer immunotherapy. It synthesizes insights from preclinical investigations and clinical trials, while also highlighting potential technological advancements that could pave the way for novel therapeutic strategies in cancer immunotherapy.

### Landscape and diversity of microbiota within tumors

While each tumor carries a distinct microbial composition, the majority in cancer typically consist of the phyla Firmicutes and Proteobacteria.^604^ Additionally, non-gastrointestinal tumors, like those in breast, lung, and ovarian cancers from the Actinobacteria phylum, often exhibit a prevalence of Micrococcaceae and Corynebacterium.^605^ Helicobacter pylori, a widely recognized cancer-associated bacterium, acts as a significant risk factor for gastric cancer and can also colonize colorectal tumors, thereby facilitating liver metastasis.^606,607^ The intricate ecosystem within tumors comprises a diverse microbial community, and obtaining a comprehensive understanding of its composition is vital for unveiling the bacterial contribution to cancer pathogenesis and tumor progression. In addition to bacteria, fungi play a significant role in cancer susceptibility. In pancreatic ductal adenocarcinoma (PDAC), the fungal community shows significant enrichment in Malassezia species.^608^ Moreover, fungi have been detected in colon, lung, prostate, stomach, and skin cancers.^609^ Chlamydial infections are widespread across various cancer types, with a notable increase observed in small-cell lung cancer tumor tissues compared to healthy controls.^610^ The pathological impact of Chlamydia in the tumor microenvironment remains a contentious topic despite its potential for malignant transformation and tumorigenesis. Viral infections are also intricately linked to cancer development. High-risk HPV types, including HPV16 and HPV18, exert a substantial pathogenic influence on cervical cancer.^611^

### Fundamental characteristics of the tumor microbiota environment

The interaction between the microbiota and the tumor microenvironment is complex and diverse, encompassing direct cell-microbe interactions and metabolite-mediated crosstalk. Pathogen-associated molecular patterns (PAMPs) are conserved elements found in bacteria, viruses, and fungi that are recognized by the pattern recognition receptors (PRRs) of the host innate immune system.^612^ Microbial compounds containing PAMPs can be classified into categories such as carbohydrates, nucleic acids, lipids, and proteins. Lipopolysaccharides (LPS), extensively studied PAMP-carrying molecules present in various Gram-negative bacteria, are notable for their potent inflammatory properties, often referred to as endotoxins.^612^ PRRs, which include Toll-like receptors (TLRs), C-type lectin receptors (CLRs), RIG-I-like receptors (RLRs), and NOD-like receptors (NLRs), are conserved receptors located on cell membranes or within cells.^613^ PRRs recognize PAMPs, initiating intracellular signaling cascades in immune cells, leading to the production of inflammatory mediators that drive systemic immune responses and adaptive immunity. The crucial interaction between PAMPs and PRRs plays a key role in stimulating either anti-tumor or pro-carcinogenic innate immune responses within the tumor microenvironment.^614^ Integration of cancer vaccines featuring PAMPs can reshape the immunosuppressive tumor microenvironment, enhancing anti-tumor immune responses and the establishment of immune memory.^615^ Overall, microbial-derived PAMPs play a significant role in modulating the tumor microenvironment and immune responses.

### Interactions mediated by microbial metabolic products

Studies have revealed that small molecule metabolites play crucial regulatory roles, capable of influencing cellular metabolism and shaping the immune system, leading to either positive or negative effects in the tumor microenvironment. Short-chain fatty acids (SCFAs), a subset of fatty acids generated through bacterial fermentation, are involved in gene expression modulation. This function is achieved by inhibiting histone deacetylases (HDACs) and activating G protein-coupled receptors (GPCRs). Such regulation impacts essential processes including metabolism, inflammatory responses, and tumor development.^616^ Bile acids are steroidal compounds primarily found in bile and are classified into primary bile acids like cholic acid and chenodeoxycholic acid, and secondary bile acids such as deoxycholic acid and lithocholic acid. Primary bile acids are synthesized by the liver, while secondary bile acids are formed from the modification of primary bile acids by bacteria in the colon.^617^ Bile acids have a critical role not only in digestion but also in cancer regulation, with secondary bile acids recognized as significant promotors of tumor growth. Polyamines, low-molecular-weight metabolites containing multiple amino groups, are produced by gut bacteria and include compounds like putrescine, spermidine, and cadaverine.^618^ Due to their antioxidant properties, polyamines can shield cells from oxidative stress and modulate immune responses by influencing macrophage function and polarization.^618^ Methylglyoxal (MGO) is an active carbonyl compound generated endogenously during glycolysis, and reactive carbonyls like MGO cause DNA damage, known as DNA glycation, leading to mutations, DNA breaks, and cell toxicity. Elevated MGO levels can compromise cell viability, while lower levels benefit tumor cell proliferation.^619^ Concerning secondary metabolites, colibactin, a mutagenic compound produced by pathogenic Escherichia coli, can promote colorectal cancer development by inducing DNA double-strand breaks.^620,621^ Peptidyl aldehydes, produced by bacteria such as Escherichia coli, Streptomyces, and Bacillus subtilis, enhance carcinogenicity by inhibiting protease activity.^622^ Thiopeptides, known for their potent antibacterial properties, are metabolized by intestinal clostridia, urogenital lactobacilli, and cutibacterium acnes on the skin. These compounds may also display anticancer effects through proteasome targeting.^623,624^ Overall, microbial metabolites, with their direct and indirect impacts on cancer development, underline the significance of exploring tumor microbiota metabolism. This approach is crucial for advancing our understanding of the tumor microenvironment and developing innovative cancer treatment strategies.

### Multi-faceted immune modulation mediated by the tumor microbiome

The presence of a tumor microbiome represents an emerging frontier in the field of tumor immunity and may exert a more potent influence compared to the microbiome in the body or intestines due to its strong local effects.^625^ For example, the tumor microbiome can influence cytokine production, leading to enhanced immune responses. Pattern recognition receptors (PRRs) are crucial in initiating the innate immune response by recognizing conserved microbial components known as pathogen-associated molecular patterns (PAMPs), including lipopolysaccharides and lipoproteins. Activation of the innate immune system serves as the initial recognition step, followed by the activation of the adaptive immune system, vital for the body’s defense against tumors. CD8^+^ T cells, essential components of the adaptive immune system, could potentially be affected by the tumor microbiome, influencing their presence.^626^ Research indicates that the presence of bacteria in tumors can boost the movement and infiltration of cytotoxic CD8^+^ T cells, potentially leading to improved outcomes for individuals with melanoma. This process is regulated by chemokines such as CCL5, CXCL9, and CXCL10.^627^ Microbiota-induced IFN can regulate tumor-targeting anti-tumor monocytes, enhance anti-cancer immunity, and boost the efficacy of immune checkpoint blockade, while the absence of microbiota can skew the tumor microenvironment toward pro-tumor macrophages.^628^

On the other hand, the tumor microbiome may induce T cell dysfunction and immune suppression through cytokine production, upregulation of immune checkpoint inhibitors, and recruitment of immune-suppressive cells.^625^ In prostate cancer, for instance, Lactobacillus johnsonii has been shown to increase the recruitment of regulatory T cells and enhance the functions of immune-inhibitory elements like PD-L1, CCL17, and CCL18. Consequently, an immune-suppressive tumor environment may be created.^629^ Furthermore, lipopolysaccharides have the ability to induce IL-6 production by activating the NF-kB signaling pathway, subsequently initiating the JAK-STAT3 signaling cascade. This activation leads to the mobilization of MDSCs and an upsurge in PD-1 expression, ultimately facilitating immune suppression.^630,631^ Additionally, microbial metabolites can also contribute to immune suppression. Tryptophan metabolites generated by Lactobacillus can activate the aryl hydrocarbon receptor (AhR) in tumor-associated macrophages. AhR activation is pivotal in the rapid progression and increased fatality rate of pancreatic carcinoma. Conversely, reducing dietary tryptophan intake can lower AhR activity in macrophages, impeding tumor growth.^632^ In summary, the tumor microbiome and its products can either enhance or suppress immunity, leading to varying effects on immune modulation. These variances stem from the specific microbial composition and distribution across different tumor types. The microbiome plays a critical role in influencing responses to immune therapies, given its diverse impact on tumor immunity. Current research suggests that the gut microbiome may affect the efficacy of anti-PD-1/L1 and anti-CTLA-4 therapies.^633^ Besides the gut microbiome, the characteristics of the tumor microbiome can significantly influence responses to immune therapies. CP1, a patient-derived prostate microbiota with local immune-stimulatory properties, can reprogram “cold” tumor microenvironments and enhance the therapeutic outcomes of anti-PD-1 immune therapy.^634^

### Intervening with the tumor microbiome to reshape the tumor microenvironment

Short-chain fatty acids (SCFAs) produced through microbial fermentation in the gut are a group of organic acids with diverse therapeutic benefits in fighting various cancers. However, their clinical use is limited due to rapid renal clearance and adverse reactions at high doses.^635^ An innovative SCFAs prodrug, based on amphiphilic copolymers, was created by conjugating SCFAs (such as butyric acid or propionic acid) to hydrophobic polymer segments via ester bonds. This formulation spontaneously forms nanocarriers suitable for oral administration, effectively inhibiting melanoma growth and metastasis.^636^ Lipopolysaccharide (LPS), a product of gram-negative bacteria, was targeted by developing a fusion protein incorporated into lipid-protein-DNA (LPD) nanoparticle systems. Selectively blocking LPS signaling led to a significant improvement in anti-PD-L1 therapy.^637^ Recent studies have shown that D-lactic acid, a gut microbiota metabolite, can convert immunosuppressive M2 macrophages to an M1 phenotype by regulating PI3K/Akt and NF-kB pathways. This study used this approach to design biologically-mimetic PLGA nanoparticles containing D-lactic acid to transform the immunosuppressive microenvironment in hepatocellular carcinoma tumors, offering a combined immunotherapy approach for this condition.^638^ In addition to microbial metabolites, the use of engineered bacterial flagellin proteins to modulate the tumor microenvironment is a promising and innovative strategy for cancer treatment.^639^ By engineering an attenuated strain of Salmonella Typhi to overexpress and secrete exogenous pathogenic Vibrio cholerae flagellin protein B (FlaB), an effective immunotherapeutic adjuvant was created that activates the TLR5 signaling pathway to initiate innate immune responses.^640^ Furthermore, specialized immunotherapeutic adjuvant clostridia, with peptidoglycan-remodeling abilities, secretes homologous NlpC/p60 peptidoglycan hydrolase SagA to produce immunomodulatory muropeptides, thus enhancing PD-1 checkpoint inhibitor cancer immunotherapy.^641^

### Genetically engineered bacterial communities designed for cancer immunotherapy

Bacterium-based artificial expression and release of therapeutic agents are now widely produced using gene engineering techniques, thanks to the progress in synthetic biology. This is because bacteria have a natural predilection for colonizing hypoxic and necrotic tumor microenvironments.^642^ The technique involves introducing tailored plasmids into bacterial cells to regulate the synthesis of specific proteins. Through the application of this approach, bacteria have been genetically engineered to produce nanobody drugs (such as CD47, PD-L1, and CTLA-4) for the treatment of tumors by locally expressing them. This facilitates targeted delivery of enhanced therapeutic doses while minimizing systemic adverse effects.^643^ Furthermore, a genetically modified probiotic was precisely engineered to reduce the immunological adverse reactions associated with concurrent treatment using anti-PD-L1 and anti-CTLA-4 agents. This probiotic, created by introducing plasmids into Escherichia coli Nissle (EcN), aims to deliver checkpoint blockade nanobodies directly to tumor sites.^643^ The probiotic employs a cleavage and release mechanism that relies on the LUXL and 4X174E genes to simultaneously express and release nanobodies resistant to PD-L1 and CTLA-4. Harnessing the crucial role of L-arginine in anti-tumor T cell responses and its synergistic effect with PD-L1 blockade, an engineered EcN strain was developed by deletion of the arginine repressor (ArgR), proficient in colonizing tumors and converting ammonia to L-arginine.^644^ The metabolism of the tumor microenvironment can be effectively regulated by these engineered bacteria, leading to improved outcomes of immunotherapy. In addition to bacteria, viruses and other microorganisms can also be harnessed for the production and delivery of therapeutic agents within the tumor microenvironment, serving as a viable approach for cancer treatment. For instance, a research project detailed the creation of a modified oncolytic vaccinia virus that can express a PD-L1 blocker and granulocyte-macrophage colony-stimulating factor (GM-CSF) in a combined manner. The synergistic action of this combination leads to increased viral replication, inhibition of PD-L1, and stimulation of GM-CSF, all working together to boost the immune response of tumor neoantigen-specific T cells. Consequently, this strategy enables effective local viral administration and eradication of distant tumors.^645^

In summary, microbial communities and their metabolic byproducts play a pivotal role in shaping the tumor microenvironment. The host immune system also influences the microbiota by modulating signals related to microorganisms or metabolic functions, thereby surveilling tumors. Furthermore, cancer can alter the composition of gut microbial communities, consequently impacting the response to immunotherapy, particularly ICB. Precision medicine of the microbiome has emerged as a promising therapeutic domain in recent years. Nonetheless, there are still numerous mysteries and questions to be explored in the future. Conducting high-quality, large-scale studies can provide robust evidence for microbial communities as adjuvants in cancer immunotherapy, potential prognostic markers, or therapeutic candidates.

## Immune therapy strategies for “cold-hot” tumors

In the last decade, there has been a significant shift in cancer therapy focus from solely targeting tumor cells to adopting more comprehensive strategies. These new approaches primarily involve activating immune cells to enhance their ability to identify and eliminate tumor cells effectively. Although these techniques have shown promise, many patients still experience disease progression. The tumor immune phenotype plays a crucial role in determining the initial response to treatment.^8,9^ To address this, a proposed classification system takes into account the types, density, and distribution of immune cell infiltrates to characterize the immune tumor status and predict the response to ICB.^6,7,23,24^ These classification systems offer insights into overcoming failures in initial treatment. This paper outlines the major tumor immune phenotypes and potential tumor characteristics that could impact the effectiveness of immune therapy, with the goal of reshaping the tumor environment, increasing T cell infiltration, engraftment, and function, thereby enhancing patients’ responses to ICB.

### Enhancement of pre-existing immunity

The effectiveness of immune regulation strategies is closely intertwined with anti-tumor immune responses, involving tumor-related or circulating immune components.^23,32,79^ Clinical efficacy of anti-PD-1 monoclonal antibody therapy in human melanoma is linked to immune activation in circulating CD8^+^ T cells that have been depleted.^31^ This discovery implies that the presence of tumor-specific T cells in the bloodstream may serve as a prognostic indicator for treatment effectiveness. Additionally, the application of ICB can induce alterations in the TCR repertoire, fostering the proliferation of specific T cell clones.^646^ Following treatment, examination of TCR profiles in melanoma patients responsive to anti-PD-1 therapy reveals a significant presence of the TCR Vß subgroup associated with the MART1 antigen, a factor previously unidentified before treatment initiation.^647^ This emergence may result from inadequate sensitivity to low-expressing clones or the emergence of new immune activations, subsequently leading to the development of T cells targeting mutated neoepitopes. Preclinical studies have demonstrated that without adequately primed and dedicated antigen-specific T cells, PD-1 blockade is ineffective in stimulating anti-tumor T cell responses.^648^ Cumulatively, these research findings strongly support the existence of an inherent and/or peripheral anti-tumor immune response, enabling subsequent checkpoint blockade therapies to exhibit significant clinical efficacy.

Effector T cells play a crucial role in eliciting anti-tumor defenses. The presence of T cells in primary colorectal cancer is significantly associated with reduced metastasis, decreased invasion, and prolonged overall survival. The success of PD-1 inhibition therapy in advanced melanoma hinges on the presence of CD8^+^ T cells localized at the tumor periphery. The proliferation of CD8^+^ T cells is closely associated with the reduction in tumor volume observed in patients displaying a favorable response post-treatment.^649,650^ Furthermore, the efficacy of PD-1 and PD-L1 inhibitors in the management of various cancers like melanoma, lung cancer, and MSI-positive colorectal cancer is closely related to the presence of a substantial mutational load in the TME.^651–653^ By disrupting tolerance, ICB can unleash pre-existing immune responses against tumors. ICB are ineffective in rejecting tumors when pre-existing responses, such as “cold” tumors, are absent.^654,655^ It is crucial to acknowledge that the quality of pre-existing immune responses also significantly influences the response to ICB.^656,657^ An example of this phenomenon is the potential emergence of adaptive resistance due to increased expression of alternative immune checkpoint molecules like CTLA-4 and TIM3 following PD-1 treatment.^657,658^ In a real-world scenario, sole inhibition of PD-1 is insufficient for the functional restoration of CD8^+^ T cells co-expressing PD-1 and TIM3, as observed in renal cell carcinoma patients.^659^ As a monotherapy, the response rate to ICB ranges from approximately 10% to 35%. Many late-stage solid tumors demonstrate limited efficacy in response to ICB due to inadequate immune infiltration from the primary tumor.^654,660^ Extensive assessment of various immunotherapeutic agents and their combinations with standard treatments in research and clinical trials aims to augment the clinical benefits of ICB.^661,662^ These combination approaches offer varying response potentials based on the tumor immune status (including “hot,” “altered,” or “cold” tumors).

### Immune therapy for “hot” tumors

In “hot” tumors, immune cells are highly active, and the microenvironment is densely infiltrated by a plethora of T cells. Immune checkpoint inhibitors (ICB) play a crucial role in reactivating the immune response of T cells against the tumor, leading to the destruction of cancer cells and imparting an immunotherapeutic effect.^10,663^ Consequently, tumors characterized by an inflammatory phenotype, known as “hot” tumors, exhibit heightened sensitivity to ICB.

#### Immune therapy targeted T cell immune checkpoints

The significant infiltration of T cells, denoted as “hot” tumors, signifies the effectiveness of either monotherapy or combination therapy of ICB. Exhausted or impaired TILs exhibit a range of inhibitory receptors, including CTLA-4 and PD-1. CTLA-4 is downregulated during the early activation and maturation of T cells in lymph nodes, while PD-1 predominantly regulates their functional responses at the tumor site.^664–666^ Various immune checkpoints manifest distinct spatiotemporal effects, as illustrated by CTLA-4 and PD-1/PD-L1.^667^ CTLA-4 primarily governs T cell activation in lymph nodes and impedes DCs activity through Treg cells. Conversely, PD-1 plays a crucial role in curtailing the activation of effector T cells and NK cells, while also promoting Treg formation in peripheral lymphoid tissues, including tumor locales.^668^ In the initial phase of the immune response, CTLA-4 restrains T cell proliferation, whereas PD-1 suppresses previously activated T cells in later stages. Treatment with anti-CTLA-4 antibodies triggers the activation of CD4^+^ T cells targeting specific antigens, leading to the elimination of regulatory T cells in the TME. Blocking the PD-1/PD-L1 signaling pathway has the potential to boost the cytotoxic activity of cytotoxic T lymphocytes within the tumor microenvironment.^61^ Hence, the simultaneous blockade of CTLA-4 and PD-1/PD-L1 with ICB is regarded as a promising therapeutic strategy in the medical field due to their unique regulatory roles in the immune system, garnering considerable attention.

The synergistic anti-tumor effects of combined dual ICB have been demonstrated in numerous clinical studies, culminating in FDA approval of these synergistic therapies for cancer management. The synergistic impact of concurrently utilizing both ICB in cancer treatment is underscored by the data presented in Table 2. FDA approval has been granted for the combined administration of Ipilimumab (targeting CTLA-4) and Nivolumab (targeting PD-1) for the treatment of melanoma (NCT01844505, NCT01927419),^669–671^ renal cell carcinoma (RCC) (NCT02231749),^672^ non-small-cell lung cancer (NSCLC) (NCT02477826),^673^ head and neck squamous cell carcinoma (HNSCC) (NCT02319044)^674^ and colorectal cancer (CRC) (NCT02060188).^675^ Melanoma was the first tumor type to receive approval for treatment with the dual combination of ICB. Recent findings from the Phase III CheckMate-067 study reveal a marked increase in median overall survival (OS) to 72.1 months with the combination of nivolumab and ipilimumab, compared to 36.9 months with nivolumab monotherapy and 19.9 months with ipilimumab monotherapy. Moreover, the combined therapy showed significantly prolonged median progression-free survival (PFS) durations of 11.5 months, 6.9 months, and 2.9 months, along with an elevated objective response rate (ORR) of 58.3%, 44.9%, and 19.0%, surpassing the individual efficacy of nivolumab or ipilimumab monotherapies (NCT01844505).^669^ In a separate Phase III study, the efficacy and safety of a dual ICB regimen were assessed against sunitinib for initial treatment of advanced clear cell renal cell carcinoma (RCC), demonstrating improved overall survival rates (75% versus 60% at 18 months) and a higher objective response rate (42% versus 27%) compared to sunitinib (registered at ClinicalTrials.gov with identifier NCT02231749).^671^ A unique Phase II clinical trial revealed that co-administration of ipilimumab and nivolumab had remarkable and long-lasting therapeutic effects in patients with advanced colorectal cancer harboring MSI-H or dMMR status (NCT02060188).^675^ Ongoing clinical trials are evaluating the efficacy of the combination of anti-CTLA-4 and anti-PD-1 antibodies in various cancer types, including prostate cancer (NCT02985957),^676^ oral cavity squamous cell carcinoma (OCSC) (NCT02919683),^677^ esophageal squamous cell carcinoma (ESCC) (NCT03143153),^678,679^ urothelial carcinoma (NCT02516241),^680^ and malignant pleural mesothelioma (MPM) (NCT02899299).^681,682^ Implementing dual ICB therapy poses challenges that need addressing, such as heightened immune-related adverse reactions (irAEs), inability to delay initial disease progression, and increased costs.

**Table 2:** Clinical trials of the ICIs combination therapy

Phase	ICIs Combination Therapy (immune checkpoint)	Doses	[n.treatment]	OS	PFS	Disease	Trial	Status	Ref.
III	**Nivolumab (PD-1) + Ipilimumab (CTLA-4)**	nivolumab (at a dose of 1 mg/kg) plus ipilimumab (at a dose of 3 mg/kg) every 3 weeks for 4 doses followed by nivolumab (3 mg/kg every 2 weeks).	[n = 314]	/	11.5 m	Melanoma	NCT01844505	Active, not recruiting	^667,668^
	Nivolumab (PD-1)	nivolumab (at a dose of 3 mg/kg) every 2 weeks.	[n = 315]	/	6.9 m				
	Ipilimumab (CTLA-4)	ipilimumab(at a dose 3 mg/kg) every 3 weeks for 4 doses.	[n = 315]	/	2.9 m				
II	**Nivolumab (PD-1) + Ipilimumab (CTLA-4)**	nivolumab (3 mg/kg, weeks 1 and 3) plus ipilimumab (1 mg/kg, given week 1 only) .	[n = 15]	/	/	OCSC	NCT02919683	Active, not recruiting	^675^
	Nivolumab (PD-1)	nivolumab (3 mg/kg, weeks 1 and 3).	[n = 14]	/	/				
II	**Nivolumab (PD-1) + Ipilimumab (CTLA-4)**	ipilimumab (3 mg/kg) plus nivolumab (1 mg/kg) once every 3 weeks for four doses, followed by nivolumab (3 mg/kg).	[n = 95]	63.8% (2-year OS rate)	51.3% (2-year PFS rate)	Melanoma	NCT01927419	Completed	^669^
	Ipilimumab (CTLA-4)	ipilimumab (3 mg/kg) pluus placebo once every 3 weeks for four doses, followed by placebo.	[n = 47]	53.6% (2-year OS rate)	12% (2-year PFS rate)				
II	**Nivolumab (PD-1) + Ipilimumab (CTLA-4)**	nivolumab 3 mg/kg plus ipilimumab 1 mg/kg once every 3 weeks (four doses) followed by nivolumab 3 mg/kg once every 2 weeks.	[n = 119]	85% (1-year OS rate)	71% (1-year PFS rate)	MMR/MSI-H CRC	NCT02060188	Active, not recruiting	^673^
III	**Nivolumab (PD-1) + Ipilimumab (CTLA-4)**	nivolumab (3 mg/kg) plus ipilimumab (1 mg/kg) every 3 weeks for four doses, followed by nivolumab (3 mg/kg) every 2 weeks.	[n = 550]	75% (18-month OS rate)	11.6 m	RCC	NCT02231749	Active, not recruiting	^670^
	Sunitinib (VEGFR2, PDGFRβ)	sunitinib (50 mg) orally once daily for 4 weeks (6-week cycle).	[n = 546]	60% (18-month OS rate)	8.4 m				
III	**Nivolumab (PD-1) + Ipilimumab (CTLA-4)**	nivolumab 3 mg/kg once every 2 weeks plus ipilimumab 1 mg/kg once every 6 weeks, nivolumab 240 mg once every 2 weeks alone.	[n = 583]	24% (5-years OS rate)	/	NSCLC	NCT02477826	Active, not recruiting	^671^
	Chemotherapy	platinum-doublet chemotherapy once every 3 weeks.	[n = 583]	14% (5-years OS rate)	/				
III	**Nivolumab (PD-1) + Ipilimumab (CTLA-4)**	nivolumab (3 mg/kg every 2 weeks) plus ipilimumab (1 mg/kg every 6 weeks)	[n = 131]	/	/	ESCC	NCT03143153	Active, not recruiting	^676,677^
	Nivolumab (PD-1) + Chemotherapy	nivolumab (240 mg every 2 weeks) plus chemotherapy (4-week cycle of fluorouracil 800 mg/m^2^ on days 1–5 and cisplatin 80 mg/m2 on day 1).	[n = 126]	/	/				
	Chemotherapy	chemotherapy (4-week cycle of fluorouracil 800 mg/m2 on days 1–5 and cisplatin 80 mg/m^2^ on day 1).	[n = 137]	/	/				
II	**Nivolumab (PD-1) + Ipilimumab (CTLA-4) (pre-Chemotherapy)**	nivolumab (1 mg/kg) plus ipilimumab (3 mg/kg)	[n = 45]	19.0 m	5.5 m	Prostate Cancer	NCT02985957	Active, not recruiting	^674^
	Nivolumab (PD-1) + Ipilimumab (CTLA-4) (post-Chemotherapy)	nivolumab (1 mg/kg) plus ipilimumab (3 mg/kg)	[n = 45]	15.2 m	3.8 m				
III	**Nivolumab (PD-1) + Ipilimumab (CTLA-4)**	nivolumab (3 mg/kg) every 2 weeks and ipilimumab (1 mg/kg) every 6 weeks.	[n = 303]	18.1 m	6.8 m	MPM	NCT02899299	Completed	^679,680^
	Chemotherapy	cisplatin (75 mg/m^2^) or carboplatin (500 mg/m^2^) plus pemetrexed in 3-week cycles for 6 cycles.	[n = 302]	14.1 m	7.2 m				
II	**Durvalumab (PD-L1) + Tremelimumab (CTLA-4)**	durvalumab (20 mg/kg every 4 weeks) plus tremelimumab (1 mg/kg every 4 weeks) for 4 cycles, followed by durvalumab (10 mg/kg every 2 weeks).	[n = 21]	7.6 m	/	HNSCC	NCT02319044	Completed	^672^
	Durvalumab (PD-L1)	durvalumab (10 mg/kg every 2 weeks) monotherapy.	[n = 8]	6.0 m	/				
	Tremelimumab (CTLA-4)	tremelimumab (10 mg/kg every 4 weeks for 7 doses then every 12 weeks for 2 doses) monotherapy.	[n = 11]	5.5 m	/				
III	**Durvalumab (PD-L1) + Tremelimumab (CTLA-4)**	durvalumab (1500 mg) plus tremelimumab (75 mg) administered intravenously every 4 weeks for up to four doses, followed by durvalumab maintenance (1500 mg) every 4 weeks.	[n = 342]	15.1 m	/	Urothelial carcinoma	NCT02516241	Active, not recruiting	^678^
	Durvalumab (PD-L1)	durvalumab monotherapy (1500 mg) administered intravenously every 4 weeks.	[n = 346]	14.4 m	/				
	Chemotherapy	gemcitabine plus cisplatin or gemcitabine plus carboplatin, depending on cisplatin eligibility administered intravenously for up to six cycles.	[n = 344]	12.1 m	/				
II/III	**Relatlimab (LAG-3) + Nivolumab (PD-1)**	relatlimab (160 mg) plus nivolumab (480 mg) in a fixed-dose combination.	[n = 355]	/	10.1 m	Advanced Melanoma	NCT03470922	Active, not recruiting	^686^
	Nivolumab (PD-1)	nivolumab (480 mg).	[n = 359]	/	4.6 m				
II	**Tiragolumab (TIGIT) + Atezolizumab (PD-L1)**	tiragolumab (600 mg) plus atezolizumab (1200 mg) every 3 weeks.	[n = 67]	/	5.4 m	NSCLC	NCT03563716	Active, not recruiting	^690^
	Atezolizumab (PD-L1)	atezolizumab (1200 mg) every 3 weeks.	[n = 68]	/	3.6 m				
II	**Nivolumab (PD-1) + Relatlimab (LAG-3) + Chemotherapy**	nivolumab (360 mg) and relatlimab (120 mg) intravenously (IV) over 60 or 30 minutes, respectively, on days 1 and 22 of each 6-week treatment cycle. Oxaliplatin (130 mg/m^2^) was administered IV on days 1 and 22 of each cycle, and capecitabine(1,000 mg/m^2^) was administered orally twice daily on days 1-14 and days 22-35 of each cycle.	[n = 138]	13.5 m	7.0 m	Gastric cancer	NCT03662659	Completed	^691^
	Nivolumab(PD-1) + Chemotherapy	nivolumab (360 mg) administered intravenously (IV) over 60 or 30 minutes, respectively, on days 1 and 22 of each 6-week treatment cycle. Oxaliplatin 130 (mg/m^2^) was administered IV on days 1 and 22 of each cycle, and capecitabine 1,000 (mg/m^2^) was administered orally twice daily on days 1-14 and days 22-35 of each cycle.	[n = 136]	16.0 m	8.3 m				

Besides the focus on PD-1/PD-L1 and CTLA-4, current clinical studies are actively investigating a novel set of ICB targeting other inhibitory receptors like TIM-3, LAG-3, TIGIT, VISTA, and BTLA. Early findings suggest that these advanced ICB exhibit promise in impeding tumor progression.^683^ LAG-3, identified as a highly favorable target for immune checkpoint blockade, operates as a suppressor of the activation and efficacy of CD4^+^ T cells, enhancing Treg cells function through interaction with MHC II molecules.^684,685^ Studies have revealed that CD8^+^ T cells co-expressing LAG-3 and PD-1 possess distinct characteristics and capabilities, hinting at the potential for combined immune checkpoint inhibitor therapy.^686^ Co-administration of anti-LAG-3 and anti-PD-1 significantly inhibits tumor growth in mice resistant to monotherapy, synergistically reinforcing the immune system.^687^ The recent Phase III RELATIVITY-047 trial (NCT03470922) showcased a notable improvement in median progression-free survival (PFS) when combining the LAG-3 inhibitor relatlimab with nivolumab as opposed to nivolumab monotherapy, yielding durations of 10.1 months and 4.6 months, respectively.^688^ TIGIT is specifically expressed on activated T cells and NK cells, promoting immune suppression by outcompeting CD226 for binding to CD155.^689^ With blocking TIGIT stimulating NK cells and blocking PD-1/PD-L1 stimulating T cells, the concurrent use of both types of ICB is viewed as a promising strategy. Research suggests that co-expression of TIGIT and PD-L1 on CD8^+^ T cells recognizing tumor antigens enhances T cell proliferation and function across various cancer types with combined blockade, showcasing significant synergy, especially in melanoma patients.^690,691^ The CITYSCAPE trial involving 135 patients demonstrated the robust efficacy and tolerability of tiragolumab, a TIGIT inhibitor, in combination with atezolizumab (an anti-PD-L1 agent) for PD-L1-positive non-small cell lung cancer (NSCLC). This combination treatment notably improved the overall response rate (ORR) to 66%, compared to 24% with atezolizumab monotherapy (NCT03563716).^692^ In a recent Phase II trial, RELATIVITY-060, examining the effectiveness and safety of combining nivolumab (a PD-1 inhibitor) and relatlimab (a LAG-3 inhibitor) with chemotherapy in newly diagnosed advanced gastric cancer (GC) or gastroesophageal junction cancer (GEJC) patients, no significant enhancement was observed in the objective response rate (ORR) compared to nivolumab and chemotherapy alone, urging further investigation into the potential benefits of introducing anti-LAG-3 with anti-PD-L1 chemotherapy for particular GC/GEJC patient subgroups (NCT03662659).^693^ Several related studies are currently ongoing, including NCT04080804, NCT03680508, and NCT04139902.

#### Combining immunotherapy with bacterial therapy in cancer treatment

The distinctive mechanisms of combining immunotherapy with bacterial therapy present a promising novel approach to cancer treatment.^694^ Certain bacterial strains, like Salmonella, possess the capacity to target selectively the hypoxic and nutrient-rich areas within tumors. Through the secretion of cytotoxins and the competition for nutrients, these bacteria can directly eradicate tumor cells.^695^ Upon the release of damage-associated molecular patterns (DAMPs) by apoptotic cancer cells, immunogenic cell death (ICD) is stimulated, reinforcing the body’s immune response against tumors. Additionally, particular bacteria and their byproducts can transform the tumor microenvironment from an immune-suppressive to an immune-activating state.^696,697^ Notably, the introduction of Staphylococcus aureus can enhance the secretion of pro-inflammatory cytokines, such as IL-12, IFN-γ, and TNF-α. This process assists in the migration of cytotoxic T lymphocytes (CTLs) and activated natural killer (NK) cells toward tumor growths, leading to subsequent regression and necrosis of melanoma in murine models.^698,699^ Furthermore, through genetic engineering, bacteria can be modified to carry specific genes responsible for producing immunostimulatory cytokines, cytotoxic agents, anti-cancer drugs, and tumor-specific antigens, demonstrating the promising potential of bacterial DNA vaccines in personalized treatment.^642^

While significant progress has been achieved in preclinical studies on bacterial cancer therapy, its clinical development has been slow. The current research is primarily in its initial stages, lacking comprehensive and well-planned clinical trials. Various challenges persist, including inherent bacterial toxicity, short half-life, and DNA instability.^700^ Thoroughly examining the safety and efficacy of combining bacterial therapy with conventional cancer treatments is paramount to overcome the limitations of monotherapy and achieve the effective elimination of tumors.^701^ Increasing evidence suggests that bacterial therapy can modulate immune responses, paving the way for integrating immunotherapy with bacterial therapy. In clinical practice, combination treatments based on immunotherapy have demonstrated the ability to mitigate bacterial toxicity while preserving tumor specificity and enhancing anti-tumor immune responses through immunomodulatory factors. Although the discussion on the impact of Salmonella on PD-L1 expression is ongoing, research has shown that the combined administration of Salmonella and anti-PD-1 treatment effectively impedes tumor progression in colorectal cancer and melanoma animal models through a synergistic effect.^702,703^ In a mouse melanoma model, the co-delivery of Salmonella VNP20009 encapsulated in polydopamine with an anti-PD-1 peptide has provoked significant immune responses against tumors, resulting in their successful eradication. Ongoing clinical trials are evaluating the effectiveness of these dual therapies. For example, a Phase I clinical trial (NCT03435952) is studying the maximum tolerable dose of Palbociclib when combined with Bacillus Calmette-Guérin-NT spores for patients with advanced solid tumors. In a separate Phase I/II clinical study (NCT02291055), researchers are assessing the efficacy and safety of ADXS11-001, a weakened form of Listeria monocytogenes, combined with Durvalumab for patients with cervix and head and neck cancer showing positivity for HPV. Currently, the study with the identifier NCT03750071 is examining the efficacy and safety of VXM01, a tumor vaccine based on weakened live Salmonella bacteria, when combined with Avelumab for individuals diagnosed with glioblastoma multiforme. Furthermore, ongoing clinical trials are investigating the synergistic effects of bacterial therapy in combination with ICB in patients. These trials include NCT03775850, NCT03595683, and NCT03637803.

Recent research suggests that the gut microbiome and its metabolic byproducts play a role in influencing the development of colitis associated with ICB.^704^ Therefore, incorporating bacterial therapy may help reduce the toxicity of ICB and enhance patient outcomes.^705^ A case report has demonstrated the efficacy of fecal microbiota transplantation (FMT) in treating refractory colitis induced by ICB.^706^ In this case, two individuals suffered severe colitis following the administration of therapies involving anti-CTLA-4 and anti-PD-1 agents. After FMT therapy from healthy donors, there was a significant alteration in the abundance and composition of gut microbiota, resulting in decreased intestinal inflammation and complete or partial relief of clinical symptoms, illustrating a remarkable outcome. The evidence from these cases suggests that bacterial-assisted therapy could help alleviate immune-related adverse events (irAEs) and improve the quality of life of patients undergoing ICB treatment. It is anticipated that genetically engineered bacteria, either used alone or in combination therapy, will soon become more accessible in clinical settings.

### The use of immunotherapy in treated “altered” tumors

The response to immune checkpoint blockade (ICB) in advanced solid tumors is linked to the presence of densely infiltrating T lymphocytes within the tumor microenvironment. This infiltration is characterized by elevated expression levels of CD4 and CD8 markers on histological slides. These tumors are commonly referred to as “hot” due to their inflammation-like features of immune cell infiltration.^21^ On the other hand, non-inflamed tumors are typically unresponsive to ICB. Additionally, “immune excluded” tumors prevent the entry of anti-tumor T lymphocytes into the tumor microenvironment, confining them to the outer regions of the tumor.^10,663^ To restore T cell generation in “immune excluded” tumors, various combination therapy strategies can be implemented, utilizing the key mechanisms outlined above:

#### Combining immunotherapy with anti-angiogenesis therapy

The uncontrolled and rapid proliferation of tumor cells disrupts the balance of blood supply, leading to tumor hypoxia, which elevates hypoxia-inducible factor-1 (HIF-1) and vascular endothelial growth factor (VEGF). Persistent tumor angiogenesis occurs within the tumor due to VEGF stimulation, forming frequently abnormal and dysfunctional neovascularization that exacerbates tumor initiation, progression, and metastasis.^707^ Anti-angiogenesis therapy, a specialized form of targeted treatment, aims at normalizing the newly formed blood vessels and is categorized into anti-VEGF drugs (e.g., bevacizumab), anti-VEGFR medications (such as ramucirumab and aflibercept), and TKIs including apatinib, sorafenib, sunitinib, axitinib, and lenvatinib.^471^ Both anti-angiogenic therapy and immunotherapy are pivotal in the tumor microenvironment, with emerging evidence indicating the existence of synergistic immune interactions between these therapeutic approaches.^67^

Tumor angiogenesis contributes to immune suppression by hindering CD8^+^ T cell penetration through disorganized neovascularization surrounding the tumor, while VEGF impedes T cell priming, suppresses CTLs activity, and hampers dendritic cell development. Additionally, tumors induce increased PD-L1 expression on associated blood vessel endothelial cells, leading to Treg cells formation and transforming macrophages into immunosuppressive M2 macrophages. Anti-angiogenic therapy normalizes aberrant tumor blood vessels, facilitating T cell infiltration, enhancing the immune system’s anti-cancer defenses, and improving the efficacy of other anti-cancer treatments.^546^ Studies have demonstrated that in individuals with metastatic colorectal cancer, bevacizumab boosts B cell and T cell levels in peripheral blood, mitigating Treg-induced immune suppression, and enhancing dendritic cell function.^708^ In contrast, ICB have limited impact on tumor angiogenesis by activating effector T cells and augmenting IFN-γ secretion.^709,710^ Concurrent PD-1 and CTLA-4 inhibition stimulates CD4^+^ T cells, promoting the restoration of tumor vascular integrity.^710^ Further research suggests a positive association between Treg differentiation and abundance and the promotion of tumor angiogenesis.^711,712^ Taken together, preclinical research findings provide a theoretical framework for the integration of immunotherapy and anti-angiogenesis therapy into clinical trials.

Numerous clinical trials have showcased impressive anti-tumor activity with combinations of immunotherapy and anti-angiogenesis therapy (Table 3). The IMmotion150 study highlighted that atezolizumab alone or paired with bevacizumab sustained daily function better than sunitinib. Notably, atezolizumab alone yielded milder symptoms than sunitinib, albeit limited sample size constraints definitive conclusions (NCT01984242).^713^ In the KEYNOTE-426 trial, pembrolizumab combined with axitinib outperformed sunitinib monotherapy in terms of objective response rate (ORR) (59.3% vs. 35.7%) and progression-free survival (PFS) (15.1 months vs. 11.1 months) (NCT02853331).^714^ In 2020, the FDA sanctioned the atezolizumab and bevacizumab duo as the first-line treatment for unresectable or metastatic hepatocellular carcinoma, post results from the Phase III multicenter IMbrave150 trial.^715^ A randomized allocation of 501 patients into a 2:1 ratio for atezolizumab-bevacizumab or sorafenib compared groups showed superior overall survival rates (67.2% vs. 54.6%) and extended progression-free survival durations (6.8 months vs. 4.3 months) favoring the atezolizumab-bevacizumab arm over sorafenib monotherapy (NCT03434379).^716,717^ Subsequent data reflected sustained, substantial improvements in patient-reported quality of life, functionality, and disease symptoms with the combination therapy surpassing sorafenib monotherapy enhancements.^718^ The pembrolizumab-lenvatinib combination displayed notable advancements in progression-free and overall survival in advanced endometrial cancer patients, outperforming chemotherapy. Confirmatory trials are planned given promising anticancer activity and safety in advanced gastric cancer (NCT03609359)^719^ and endometrial cancer (NCT02501096).^720^ This combination approach exhibits potential in treating these cancers. Additionally, investigations have explored potential biomarkers predicting the efficacy of combined therapy. A Phase II trial demonstrated that a simultaneous adminisertation of camrelizumab and apatinib with liposomal paclitaxel and nedaplatin as initial therapy exhibited promising antitumor efficacy and tolerable safety profiles in advanced esophageal squamous cell carcinoma (ESCC) patients, warranting further randomized clinical investigations to assess the combination’s effectiveness (NCT03603756).^721^ In the KEYNOTE-061 trial, pembrolizumab as second-line monotherapy showed no substantial overall survival enhancement compared to chemotherapy; however, blending a second-line PD-1 inhibitor with albumin-bound paclitaxel and apatinib displayed a specific degree of effectiveness and safety in metastatic gastric cancer (mGC) patients (NCT04182724).^722^ Data from the JAVELIN Renal 101 study indicated that in advanced renal cell carcinoma (RCC) individuals, avelumab (a PD-L1 inhibitor) combined with axitinib significantly improved progression-free survival compared to sunitinib (NCT02684006).^723,724^ Biomarker analysis linked enhanced PFS with novel gene expression signatures relating to immune regulation and angiogenesis, newly identified mutation GESs, and specific HLA types, yet no clear association was found with variables like PD-L1 expression or TMB.^725^ Further research is essential to identify specific biomarkers reliably predicting the efficacy of combined therapies, as these biomarkers remain undiscovered to date.

**Table 3:** Clinical trials of immunotherapy combined with anti-angiogenic therapy

Phase	ICIs Combination Therapy (immune checkpoint)	Doses	[n.treatment]	OS	PFS	Disease	Trial	Status	Ref.
II	**Atezolizumab (PD-L1) + Bevacizumab (VEGF-A)**	atezolizumab (1200 mg) intravenously (i.v.) every 3 weeks	[n = 101]	/	11.7 m	RCC	NCT01984242	Completed	^711^
	Atezolizumab (PD-L1)/Sunitinib (VEGFR2, PDGFRβ)	atezolizumab regimen plus bevacizumab (15 mg/kg) i.v. every 3 weeks (n = 101), or sunitinib (50 mg) orally daily 4 weeks on, 2 weeks off.	[n = 101]	/	6.1 m/8.4 m				
III	**Pembrolizumab (PD-1) + Axitinib (VEGFR)**	pembrolizumab (200 mg) intravenously once every 3 weeks plus axitinib (5 mg) orally twice daily	[n = 432]	/	15.1 m	RCC	NCT02853331	Active, not recruiting	^712^
	Sunitinib (VEGFR2, PDGFRβ)	sunitinib (50 mg) orally once daily for the first 4 weeks of each 6-week cycle	[n = 429]	35.7 m	11.1 m				
III	**Atezolizumab (PD-L1) + Bevacizumab (VEGF-A)**	atezolizumab (1200 mg) plus bevacizumab (15 mg/kg) of intravenously every 3 weeks	[n = 336]	19.2 m	6.8 m	HCC	NCT03434379	Completed	^714,715^
	Sorafenib (Raf-1, B-Raf, VEGFR-3)	sorafenib (400 mg) orally twice daily	[n = 165]	13.4 m	4.3 m				
II	**Pembrolizumab (PD-1) + Lenvatinib (VEGFR2/VEGFR3)**	lenvatinib (20 mg) oral daily plus pembrolizumab (200 mg) intravenous every 3 weeks	[n = 29]	/	7.1 m	Gastric cancer	NCT03609359	Completed	^717^
IB/II	**Pembrolizumab (PD-1) + Lenvatinib (VEGFR2/VEGFR3)**	lenvatinib (20 mg) once daily orally plus pembrolizumab (200 mg) intravenously once every 3 weeks, in 3-week cycles	[n = 108]	7.4 m	21.1 m	Endometrial cancer	NCT02501096	Completed	^718^
II	**Camrelizumab (PD-1) + Apatinib (VEGFR2) + Chemotherapy**	camrelizumab (200 mg), liposomal paclitaxel (150 mg/m^2^), and nedaplatin (50 mg/m^2^) on day 1, and apatinib (250 mg) on days 1-14	[n = 30]	19.43 m	6.85 m	ESCC	NCT03603756	Unknown	^719^
I/II	**Camrelizumab (PD-1) + Apatinib (VEGFR2) + Nab-paclitaxel**	PD-1 inhibitor (selected according to patients’ requirements) in combination with albumin paclitaxel (125 mg/m^2^, intravenously, days 1 and 8, or 250 mg/m^2^, intravenously, day 1) and apatinib (250 or 500 mg, orally, days 1-21) every 3 weeks	[n = 43]	10.1 m	6.2 m	Metastatic gastric cancer	NCT04182724	Unknown	^720^
III	**Avelumab (PD-L1) + Axitinib (VEGFR)**	avelumab (10 mg/kg) intravenously every 2 weeks plus axitinib (5 mg) orally twice daily	[n = 442]	0.59 (95% CI 0.38-0.93)	0.79 (95% CI 0.53-1.16)	RCC	NCT02684006	Active, not recruiting	^721,722^
	Sunitinib (VEGFR2, PDGFRβ)	sunitinib (50 mg) orally once daily for 4 weeks (6-week cycle)	[n = 444]	0.86 (95% CI, 0.55-1.34)	1.15 (95% CI 0.77-1.70)				

#### cGAS-STING agonists

In the continuum of cancer immunity, innate immunity, alongside adaptive immunity, holds crucial significance. Serving as a pivotal regulatory factor and mediator between the innate and adaptive immune systems, the cGAS-STING pathway plays a vital role in this intricate network. During tumor progression, the interaction between externally released DNA from tumor cells and the cytosolic DNA sensor cGAS triggers the production of cyclic GMP-AMP (cGAMP). Subsequently, this process activates STING, leading to the secretion of type I interferons (IFNs) and pro-inflammatory cytokines.^726^ These elements, upon release, stimulate dendritic cells, thereby activating T cells and NK cells.^170^ The STING pathway is frequently suppressed in various cancer types, such as colorectal cancer and melanoma, contributing to a potent anti-tumor immune response.^727,728^ Activation of the cGAS-STING pathway has shown significant efficacy in inhibiting tumor metastasis. Progressing to clinical trial phases based on the promising potential of STING agonists in cancer treatment are several medications, including ADU-S100,^726^ E7766,^729^ MK-1454,^730^ BMS-986301, and SB-1128.^731^ Preclinical research findings suggest that STING agonists exhibit immunosuppressive properties in certain tumor types. For instance, in HPV-associated tongue squamous cell carcinoma, activated STING has been observed to enhance Treg cell infiltration.^732^ Activation of STING agonists induces T cell suppression and immune tolerance in lewis lung carcinoma (LLC) via an IDO-mediated pathway.^588^ In an ovarian cancer mouse model, a correlation has been noted between STING activation and increased PD-L1 expression, demonstrating a link between STING activation and PD-L1 upregulation.^733^ Consequently, the use of ICB may counteract the tumor-promoting effects of STING agonists, potentially transforming immunologically “cold” tumors into “hot” tumors and suggesting a more potent treatment strategy with combination therapy. Preclinical studies have shown that the combined use of STING agonists and ICB enhances anti-tumor efficacy (Table 4).^734,735^ For instance, in advanced mouse models bearing MC38 and B16F10 tumors, the administration of a combination of MK-1454 and the anti-PD-1 antibody mDX400 resulted in a noteworthy reduction in tumor size.^730^ However, in patients with unresectable or advanced melanoma, the simultaneous use of Epacadostat at a dosage of 100 mg with pembrolizumab did not demonstrate improved progression-free survival or overall survival when compared to pembrolizumab paired with a placebo. The efficacy of IDO1 inhibition as a strategy to enhance the effectiveness of anti-PD-1 therapy in cancer remains uncertain (NCT02752074).^303^

**Table 4:** Clinical trials of the ICIs therapy combined with other immunotherapy strategies

Phase	ICIs Combination Therapy (immune checkpoint)	Doses	[n.treatment]	OS	PFS	Disease	Trial	Status	Ref.
III	**Pembrolizumab (PD-1) + Epacadostat (IDO)**	epacadostat (100 mg) orally twice daily plus pembrolizumab (200 mg) intravenously every 3 weeks.	[n = 354]	74.4% (12-month OS rate)	23 m	Melanoma	NCT02752074	Completed	^302^
	Pembrolizumab (PD-1)	placebo plus pembrolizumab (200 mg) intravenously every 3 weeks.	[n = 352]	74.1% (12-month OS rate)	4.7 m				
I	**Durvalumab (PD-L1) + Pexidartinib (CSF1)**	**/**	[n = 47]	/	/	Metastatic/Advanced Pancreatic or Colorectal Cancers	NCT02777710	Completed	^737^
Ib	**Pembrolizumab (PD-1)** + **ARRY-382 (CSF1)**	ARRY-382 [starting dose 200 mg once daily (QD) orally] plus pembrolizumab [2 mg/kg intravenously (IV) every 3 weeks (Q3W)]	[n = 19]	/	/	Solid tumors	NCT02880371	Terminated	^738^
I	**Pembrolizumab (PD-1) + Maraviroc (CCR5)**	pembrolizumab 200 mg intravenously every 21 days in combination with maraviroc 300 mg orally twice daily for a maximum total of eight cycles	[n = 20]	9.83 m	2.1 m	mCRC	NCT03274804	Completed	^739^
II	**Pembrolizumab (PD-1)** + **BL-8040 (CXCR4)**	BL-8040 monotherapy (1.25 mg/kg) on days 1-5 of week, followed by pembrolizumab every 3 weeks (200 mg, intravenous)	[n = 37]	3.3 m	/	mPDAC	NCT02826486	Completed	^740^
IIb	**Pembrolizumab (PD-1)** + **mRNA-4157 (V940)**	mRNA-4157 was administered intramuscularly (maximum nine doses) and pembrolizumab intravenously (maximum 18 doses) in 3-week cycles	[n = 107]	/	/	Melanoma	NCT03897881	Recruiting	^801^
	Pembrolizumab (PD-1)	pembrolizumab intravenously (maximum 18 doses) in 3-week cycles	[n = 50]	/	/				
II	**Ipilimumab (CTLA-4) + Talimogene laherparepvec (T-VEC)**	T-VEC was administered intratumorally in week 1 (10(6) plaque-forming units/mL), then in week 4 and every 2 weeks thereafter (10(8) plaque-forming units/mL). Ipilimumab (3 mg/kg) was administered intravenously every 3 weeks for four infusions, beginning in week 6.	[n = 98]	54.7% (5-year OS rate)	13.5 m	Melanoma	NCT01740297	Completed	^807,808^
	Ipilimumab (CTLA-4)	Ipilimumab (3 mg/kg) was administered intravenously every 3 weeks for four infusions, beginning in week 6.	[n = 100]	48.4% (5-year OS rate)	6.4 m				
III	**Nivolumab (PD-1)** + **BEMPEG (IL-2)**	BEMPEG was intravenously at a dose of 0.006 mg/kg, sequentially followed by intravenous nivolumab administration at a dose of 360 mg, once every 3 weeks.	[n = 391]	29.67 m	4.17 m	Melanoma	NCT03635983	Completed	^813^
	Nivolumab (PD-1)	nivolumab monotherapy administered intravenously at a dose of 360 mg once every 3 weeks	[n = 392]	28.88 m	4.99 m				
I/II	**Nivolumab (PD-1)** + **BEMPEG (IL-2)**	EMPEG 0.006 mg/kg plus nivolumab 360 mg intravenously every 3 wk	[n = 41]	23.7 m	4.1 m	Advanced/mUC	NCT02983045	Completed	^812^
Ib	**Pembrolizumab (PD-1) + Utomilumab (4-1BB/CD137)**	Utomilumab (0.45-5.0 mg/kg) and pembrolizumab (2 mg/kg) were administered intravenously every 3 weeks.	[n = 23]	/	/	Advanced Solid Tumors	NCT02179918	Completed	^822^
II	**Nivolumab (PD-1) + Sotigalimab (CD40) + Chemotherapy**	Nivolumab (1000 mg/m^2^),Sotigalimab (0.1 mg/kg), nab-paclitaxel (125 mg/m^2^)	[n = 35]	41.3% (1-year OS rate)	6.7 m	mPDAC	NCT03214250	Completed	^823^

#### Targeted therapy for tumor-associated macrophages

Macrophages exhibit essential and versatile roles, primarily classified into two main subtypes: M1 and M2. M1 macrophages excel in eliminating tumor cells and antigen presentation, consequently promoting anti-tumor adaptive immune responses. Conversely, the predominant macrophages within tumor microenvironments (TAMs) belong to the M2 category, fostering tumorigenesis and displaying anti-inflammatory traits.^736^ Tumor-associated macrophages (TAMs) create a supportive tumor microenvironment (TME) conducive to tumor progression and metastasis by releasing growth factors, NF-κB, and pro-angiogenic agents. These macrophages also establish an immunosuppressive milieu in the TME, limiting antigen presentation abilities through the secretion of immunosuppressive factors like IL-1, IL-10, and TNF-α, enhancing immune-regulatory elements such as PD-L1, MHC-I, and CD80, and recruiting additional immunosuppressive cells, collectively contributing to immune evasion.^737^ Clinical examination of tumor specimens has uncovered a correlation between PD-L1 expression and TAM infiltration, indicating the potential effectiveness of combined targeted therapy for TAMs with ICB therapy to enhance therapeutic outcomes.^738^

Therapeutic strategies targeting TAMs can be categorized into three primary approaches: inhibiting TAM viability by targeting colony-stimulating factor 1 (CSF1) and its receptor CSF1R, preventing TAM recruitment through interventions targeting CCL2, CCR2, and CXCR4, and reprogramming TAMs using CD47 blockers, CD40 stimulants, and Toll-like receptor (TLR) activators.^738^ Among these approaches, targeting the CSF1/CSF1R axis has been extensively researched due to its critical role in TAM survival, differentiation, and activation **(**Table 4**)**. Preclinical studies in animal models of colorectal cancer, hepatocellular carcinoma, and esophageal adenocarcinoma have demonstrated promising antitumor effects with the combination of anti-PD-L1 therapy and the CSF1R inhibitor pexidartinib, modulating TAM infiltration and enhancing CD8^+^ T cell activation (NCT02777710).^739^ However, the combination of AMG 820 (anti-CSF1R) and pembrolizumab exhibited adequate safety in adult patients with advanced solid tumors, with efficacy improvements needed(NCT02880371).^740^ Results from the PICCASSO and COMBAT trials suggest a potential strategy to target TAM recruitment by combining pembrolizumab with either maraviroc (anti-CCR5) (NCT03274804)^741^ or BL-8040 (anti-CXCR4) (NCT02826486)^742^ in colorectal cancer and pancreatic ductal adenocarcinoma (PDAC), showing moderate clinical efficacy in these cancer types. Ongoing clinical trials are exploring the combination of TAM-targeted therapy with immunotherapy, as seen in studies such as NCT02452424 and NCT02777710.

#### Regulators of T cell migration

In tumors where CD8^+^ T cells are excluded, these cells typically gather at the periphery of the tumor, indicating the potential for the host to initiate a T cell-driven immune response. However, these cells face barriers that hinder their penetration into the central region of the tumor. The exclusion of T cells could be attributed to the lack of signals attracting these cells, such as chemokines like CXCL9, CXCL10, CXCL11, CXCL13, CX3C chemokine ligand 1 (CX3CL1), as well as CCL2 and CCL5.^59,743,744^ The diminished levels of these chemokines and their regulation may result from dysregulated oncogenic, genetic, and epigenetic pathways. In ovarian and colorectal cancer cases, alterations in histone modifications and DNA methylation could lead to the suppression of CXCL9 and CXCL10 expression from TH1 cells.^231,745^ Focusing on epigenetic modulation holds promise for enhancing the functionality of effector T cells infiltrating tumors, halting tumor progression, and bolstering the effectiveness of PD-L1 inhibition in preclinical scenarios.^745,746^

Metastatic melanoma exhibits persistent activation of the beta-catenin pathway, leading to a compromised recruitment of CD103^+^ DCs in the tumor microenvironment. This recruitment defect causes a deficiency of CXCL9 and CXCL10, both originating from CD103^+^ DCs, in “hot” tumors.^747^ The cited studies are significant as they distinguish between T cell-inflamed (hot) and non-T cell-inflamed (cold) tumors. Thus, it remains speculative to suggest the potential exclusion of tumors falling between these two categories. Invasive bladder cancer involving the MIUBC activates tumor oncogenic pathways associated with T cell exclusion, which is typically indicative of a poor prognosis.^748^ In T cell-inflamed muscle-invasive urothelial bladder cancer (MIUBC), commonly upregulated genes include those encoding PD-L1, IDO, and FOXP3 (the main regulator of Treg cells). Conversely, non-T cell-inflamed MIUBC frequently exhibits activations in the beta-catenin, peroxisome proliferator-activated receptor-gamma (PPARγ), and fibroblast growth factor receptor 3 (FGFR3) pathways.^748^ For successful responses to anti-PD-L1 therapy in mice, the presence of “sufficient” T cell infiltration at the tumor site is considered more crucial than variations in PD-L1 expression levels, laying the foundation for potential strategic developments.^421^

### Treated “Cold” tumors with immunotherapy

“Cold” tumors, characterized by low immune scores, present a significant challenge for eradication and are often associated with poor prognoses. One treatment strategy focuses on addressing the limited pre-existing immune response by enhancing targeted therapies that promote T-cell responses. This may involve the use of vaccines, autologous T-cell transplantation (ACT), or techniques that transform tumors into vaccines. Additionally, this approach may entail blocking co-inhibitory signals by reducing ICB and providing co-stimulatory signals such as anti-OX40 or anti-GITR in combination.^10,21,749^ However, a concern with this strategy is the simultaneous increase in adverse effects, a common issue in many combination therapies, requiring thorough evaluation. The concurrent activation of multiple pro-tumor mechanisms ultimately contributes to the development of “cold” tumors, necessitating a combination of strategies to achieve clinical benefits.

#### Combination radiotherapy with immunotherapy

Radiotherapy utilizes high levels of ionizing radiation to directly target cellular DNA, aiming to eliminate cancer cells, reduce tumor size, and alleviate tumor burden. This treatment approach has significant implications for the immune system. In contrast, radiotherapy triggers immunogenic cell death (ICD) in cancer cells by facilitating the release of damage-associated molecular patterns (DAMPs) from the outer layer of tumor cells. The key components of these damage-related molecular patterns, specifically DAMPs, comprise heat shock proteins, the high-mobility group box 1 protein (HMGB1), and adenosine triphosphate (ATP). The liberation of DAMPs amplifies the maturation of dendritic cells (DCs), enhances their ability to present antigens, promotes the secretion of cytokines like IL-2, IL-4, and IFN-γ, ultimately fostering an intensified anti-tumor immune response.^750,751^ Moreover, radiotherapy has the capability to provoke the release of inflammatory mediators such as interferons, interleukin-1β, and CXCL9. By facilitating the migration of DCs and activated T cells into the tumor microenvironment (TME), these agents reshape its immune landscape. For instance, radiation therapy induces elevated levels of tumor necrosis factor, resulting in a substantial reduction in the population of myeloid-derived suppressor cells (MDSCs).^752^ Numerous investigations have revealed the immunosuppressive effects of radiotherapy, leading to bone marrow suppression, decreased peripheral blood cell counts, and heightened expression of negative immune checkpoint ligands like PD-L1. This elevation may enhance the susceptibility of tumor cells to immune checkpoint blockade.^752,753^ Furthermore, radiotherapy induces the secretion of immune-suppressive cytokines and chemokines, including TGF-β, IL-6, and CXCL12, facilitating the recruitment of regulatory immune cells to tumors, such as MDSCs, regulatory T cells (Treg cells), and alternatively activated macrophages (M2).^754^ Overall, these findings provide a theoretical rationale for combining radiotherapy with immunotherapy.

In specific clinical contexts and animal models, an intriguing observation has been made regarding the impact of local radiotherapy, not only on shrinking the primary tumor site but also on diminishing the occurrence of distant metastases. Termed the abscopal effect of radiotherapy, this phenomenon has caught significant attention.^755^ Potential mechanisms underlying this phenomenon involve the long-distance migration of activated effector T cells, enhancement of dendritic cell functionality, and the release of diverse cytokines.^756^ Studies have demonstrated a strong correlation between tumor radiosensitivity, the manifestation of the abscopal effect, and the functionality of the host immune system. Recent investigations suggest that immunotherapy could effectively counteract immune suppression and enhance the abscopal effect in various malignancies like colorectal and prostate cancer.^757,758^ Phase I/II studies from three institutions have reported that the absolute lymphocyte count can predict the distant response of patients undergoing combined immunotherapy and radiotherapy in terms of biomarkers.^759^

In addition to the distant response, combining radiotherapy with various immunotherapeutic approaches, such as ICB, cytokines, and co-stimulatory antibodies, has demonstrated the enhancement of the anti-tumor immune response. This combination strategy boosts radiotherapy-induced immune activation and counteracts its immune suppressive effects in multiple cancer types (Table 5). Notably, the phase III PACIFIC trial exhibited significant benefits in patients with stage III non-small cell lung cancer through the incorporation of durvalumab following chemoradiotherapy. The study showed improved overall survival rates at 24 months (66.3% vs. 55.6%, *P* = 0.005) and prolonged progression-free survival (17.2 vs. 5.6 months), with well-tolerated safety profiles (NCT02125461).^760,761^ Furthermore, a Phase III clinical trial (CA184-043) involving 799 patients with metastatic castration-resistant prostate cancer previously treated with docetaxel aimed to evaluate the efficacy of ipilimumab post-radiotherapy. After a median follow-up of 2.4 years, the radiotherapy and ipilimumab combination demonstrated significant enhancements in overall survival rates at 2, 3, 4, and 5 years compared to placebo (25.2% vs. 16.6%, 15.3% vs. 7.9%, 10.1% vs. 3.3%, 7.9% vs. 2.7%). These results indicate a synergistic antitumor effect of combining radiotherapy with immune checkpoint blockade in the context of prostate cancer (NCT00861614).^762^ Similarly, stereotactic body radiotherapy (SBRT) followed by pembrolizumab treatment exhibited good tolerability and a doubled objective response rate, although it did not reach the predefined clinically meaningful endpoint. Larger trials are required to determine if radiotherapy can transform non-inflammatory non-small cell lung cancer into a more inflammatory tumor microenvironment (NCT02492568).^763^ Results from similar studies suggest that the combination of SBRT with pembrolizumab and trametinib could be a potential treatment option for postoperative patients with locally recurrent pancreatic cancer, pending further validation through additional phase III trials (NCT02704156).^764^

**Table 5:** Clinical trials of immunotherapy combined with radiotherapy

Phase	ICIs Combination Therapy (immune checkpoint)	Doses	[n.treatment]	OS	PFS	Disease	Trial	Status	Ref.
III	**Durvalumab (PD-L1) + Chemoradiation therapy**	Randomization occurred 1 to 42 days after the patients had received chemoradiotherapy. durvalumab intravenously, at a dose of 10 mg per kilogram of body weight	[n = 473]	47.5 m	16.9 m	NSCLC	NCT02125461	Completed	^758,759^
	Chemoradiation Therapy	Randomization occurred 1 to 42 days after the patients had received chemoradiotherapy.placebo every 2 weeks for up to 12 months	[n = 236]	29.1 m	5.6 m				
III	**Ipilimumab (CTLA-4)** + **RT**	bone-directed radiotherapy (8 Gy in one fraction) followed by ipilimumab 10 mg/kg every 3 weeks for up to four doses	[n = 399]	7.9% (5-year OS rate)	/	Prostate cancer	NCT00861614	Completed	^760^
	RT	bone-directed radiotherapy (8 Gy in one fraction) followed by either placebo every 3 weeks for up to four doses	[n = 400]	2.7% (5-year OS rate)	/				
II	**Pembrolizumab (PD-1)** + **SBR**	Pembrolizumab (200 mg/kg every 3 weeks) after radiotherapy (3 doses of 8 Gy) (experimental arm)	[n = 36]	15.9 m	6.6 m	NSCLC	NCT02492568	Completed	^761^
	Pembrolizumab	Pembrolizumab (200 mg/kg every 3 weeks)	[n = 40]	7.6 m	1.9 m				
II	**Pembrolizumab (PD-1)** + **SBRT + Trametinib(MEK)**	SBRT with doses ranging from 60-65 Gy in five fractions, intravenous pembrolizumab 200 mg once every 3 weeks, and oral trametinib (2 mg) once daily	[n = 29]	13.6 m	7.9 m	Pancreatic cancer	NCT02704156	Active, not recruiting	^762^
	SBRT + Gemcitabine	SBRT (same regimen) and intravenous gemcitabine (1000 mg/m^2^) on day 1 and 8 of a 21-day cycle for eight cycles	[n = 34]	12.4 m	4.3 m				
II	**Sipuleucel-T** + **RT**	sipuleucel-T alone (Arm A) or sipuleucel-T initiated 1 week after completing sensitizing RT to single metastatic site (Arm B). RT was delivered at 300 cGy/day to 3000 cGy total.	[n = 25]	/	3.65 m	Prostate cancer	NCT01807065	Completed	^763^
	Sipuleucel-T	sipuleucel-T alone (Arm A) or sipuleucel-T initiated 1 week after completing sensitizing RT to single metastatic site (Arm B). RT was delivered at 300 cGy/day to 3000 cGy total.	[n = 24]	/	2.46 m				

Patients with early-stage non-small cell lung cancer (NSCLC) that has minimally spread may derive benefit from local ablative therapy (LAT), such as surgical procedures or stereotactic radiotherapy. Sipuleucel-T, a personalized cellular immunotherapy designed for patients with asymptomatic or minimally symptomatic metastatic castration-resistant prostate cancer (mCRPC), was not previously studied in individuals who had undergone radiation therapy (RT) within 28 days prior to sipuleucel-T treatment initiation, given its potential to suppress bone marrow function and immune response. A phase II study indicated that pre-treatment sensitizing radiation therapy administered one week before generating sipuleucel-T had no impact on the delivery or efficiency of sipuleucel-T treatment. Furthermore, it was observed that radiation therapy did not enhance the humoral and cellular responses associated with mononuclear cell T cell therapy (NCT01807065).^765^ These findings suggest that the benefits of combined therapy are specific to certain patient subgroups, emphasizing the importance of identifying and selecting individuals most likely to respond positively. Recent research findings on biomarkers characterizing the immune activation state following the combination therapy of radiotherapy and immunotherapy were summarized in a recent review. An integrated model was proposed, involving the analysis of peripheral blood samples, histological specimens, and medical imaging reports.^766^ Further clinical trials are warranted to investigate the optimal timing, dosage, site, and sequence of radiotherapy for maximizing effectiveness.

#### Combination chemotherapy with immunotherapy

Chemotherapy utilizes potent chemical agents to target rapidly dividing tumor cells and exerts bidirectional immune modulation effects on the tumor microenvironment (TME). While a common belief in the past suggested that chemotherapy could induce myelosuppression, hinder anti-tumor immunity, and promote immune tolerance and suppression, extensive research has evidenced that chemotherapy can actually enhance anti-tumor immune responses over time.^767^ One way chemotherapy impacts tumor immunity is through direct modifications to tumor cells, including enhancing tumor cell antigenicity (e.g., with cyclophosphamide, gemcitabine, platinum agents, and paclitaxel),^767^ inducing immunogenic cell death (ICD) in tumor cells, and eliciting specific immune responses (as seen with anthracycline and oxaliplatin).^768^ Chemotherapy may increase the levels of MHC-I molecules and B7-1 on cell surfaces, observed notably with drugs like etoposide, topotecan, and paclitaxel, and enhance the vulnerability of tumor cells to destruction by cytotoxic CD8^+^ T cells and NK cells, seen in drugs like paclitaxel, cisplatin, and doxorubicin.^769^ Conversely, chemotherapy stimulates immune responses within the TME by interacting with various immune cells, such as inhibiting immune-suppressive cells like regulatory T cells, myeloid-derived suppressor cells, and M2 macrophages, while activating dendritic cells, NK cells, and effector T cells using agents like paclitaxel, doxorubicin, and cisplatin.^770^ It is crucial to note that the complexity of the TME may sometimes compromise anti-tumor immune responses, potentially leading to a resurgence of immunosuppressive effects. In such scenarios, simultaneous administration of ICB may enhance tumor responsiveness to chemotherapy. In summary, these findings endorse the synergistic integration of chemotherapy with immunotherapy.

Numerous clinical trials have demonstrated that combining chemotherapy with immunotherapy yields prognostic benefits compared to chemotherapy alone, leading to therapeutic applications in cancer treatment (Table 6), including melanoma (NCT03666143^771^, NCT00324155^772^), gastroesophageal junction cancer (G/GEJ cancer) (NCT02494583^773,774^, NCT02872116^775,776^, NCT04250948^777^), urothelial cancer (NCT02853305^778^), non-small cell lung cancer (NSCLC) (NCT02578680^779,780^, NCT02775435^781,782^, NCT02039674^783^, NCT03409614^784,785^, NCT04033354^786^), triple-negative breast cancer (TNBC) (NCT02819518^787,788^, NCT03036488^789^, NCT02425891^790,791^), gastric cancer (NCT03675737^792^), esophageal squamous cell carcinoma (ESCC) (NCT03691090^793^), oral squamous cell carcinoma (OSCC) (NCT03783442^794^). An illustration of this is a clinical study demonstrating that the overall survival of previously untreated metastatic melanoma patients was enhanced when ipilimumab (administered at a dosage of 10 mg/kg) was combined with dacarbazine, as opposed to dacarbazine used in conjunction with a placebo (NCT00324155).^772^ In the worldwide Phase II clinical trial ASTRUM-004, it was noted that incorporating serplulimab into chemotherapy resulted in a significant improvement in median overall survival, displaying a hazard ratio of 0.73 (95% CI 0.58-0.93; *P* = 0.010). Notably, the occurrence of adverse events of grade 3 or above, attributed to serplulimab or the placebo, was recorded in 126 cases (35.2%) and 58 cases (32.4%) respectively. The study results suggest that adding serpluliumab to chemotherapy significantly enhances survival in advanced squamous NSCLC patients and is well-tolerated (NCT04033354).^786^ In patients with early-stage triple-negative breast cancer, the administration of pembrolizumab combined with neoadjuvant chemotherapy resulted in a markedly higher rate of achieving a pathological complete response, as opposed to patients who received a placebo in addition to neoadjuvant chemotherapy. The FDA has granted approval for the utilization of pembrolizumab in conjunction with chemotherapy to effectively treat inoperable recurrent/metastatic TNBC exhibiting PD-L1 expression. This ruling was supported by findings from the KEYNOTE-355 Phase III trial, demonstrating a notable enhancement in progression-free survival among individuals with PD-L1-positive TNBC who received treatment involving pembrolizumab and chemotherapy. The combination therapy, in particular, led to a significant enhancement in progression-free survival when compared to chemotherapy as a standalone treatment (9.7 months vs. 5.6 months), and demonstrated a tolerable safety profile (NCT02819518).^787,788^

**Table 6:** Clinical trials of immunotherapy combined with chemotherapy

Phase	ICIs Combination Therapy (immune checkpoint)	Doses	[n.treatment]	OS	PFS	Disease	Trial	Status	Ref.
III	**Pembrolizumab (PD-1) + Chemotherapy**	pembrolizumab (200 mg) plus chemotherapy (cisplatin 80 mg/m^2^/d on day 1 plus fluorouracil 800 mg/m^2^/d on days 1 to 5 or capecitabine 1000 mg/m^2^ twice daily) every 3 weeks	[n = 257]	PD-L1 score ≥1 population:16.5 m;PD-L1 score ≥10 population:17.5 m	PD-L1 score ≥1 population:6.9 m	G/GEJ cancer	NCT02494583	Completed	^771,772^
	Chemotherapy	chemotherapy (cisplatin 80 mg/m^2^/d on day 1 plus fluorouracil 800 mg/m^2^/d on days 1 to 5 or capecitabine 1000 mg/m^2^ twice daily) every 3 weeks	[n = 250]	PD-L1 score ≥1 population:13.8 m;PD-L1 score ≥10 population:14.8 m	PD-L1 score ≥1 population:6.4 m				
III	**Pembrolizumab (PD-1) + Chemotherapy**	pembrolizumab (200 mg) every 3 weeks plus chemotherapy (nab-paclitaxel; paclitaxel; or gemcitabine plus carboplatin)	[n = 566]	PD-L1 score ≥1 population:17.6 m;PD-L1 score ≥10 population:23.0 m	/	TNBC	NCT02819518	Completed	^785,786^
	Chemotherapy	placebo plus chemotherapy	[n = 281]	PD-L1 score ≥1 population:16.0 m;PD-L1 score ≥10 population:16.1 m	/				
III	**Pembrolizumab (PD-1) + Chemotherapy**	pembrolizumab (200 mg) every 3 weeks for a maximum of 35 cycles plus intravenous chemotherapy (gemcitabine [1000 mg/m^2^] on days 1 and 8 and investigator’s choice of cisplatin [70 mg/m^2^] or carboplatin [area under the curve 5] on day 1 of every 3-week cycle) for a maximum of six cycles	[n = 351]	17.0 m	8.3 m	Urothelial cancer	NCT02853305	Completed	^776^
	Chemotherapy	chemotherapy (gemcitabine [1000 mg/m^2^] on days 1 and 8 and investigator’s choice of cisplatin [70 mg/m^2^] or carboplatin [area under the curve 5] on day 1 of every 3-week cycle) for a maximum of six cycles	[n = 352]	14.3 m	7.1 m				
III	**Pembrolizumab (PD-1) + Chemotherapy**	pemetrexed and a platinum-based drug plus 200 mg of pembrolizumab every 3 weeks for 4 cycles, followed by pembrolizumab or placebo for up to a total of 35 cycles plus pemetrexed maintenance therapy	[n = 410]	5-year OS rates:19.4%	/	NsqNSCLC	NCT02578680	Completed	^777,778^
	Chemotherapy	pemetrexed and a platinum-based drug plus placebo every 3 weeks for 4 cycles, followed by pembrolizumab or placebo for up to a total of 35 cycles plus pemetrexed maintenance therapy	[n = 206]	5-year OS rates:11.3%	/				
III	**Pembrolizumab (PD-1) + Chemotherapy**	200 mg of pembrolizumab for up to 35 cycles; all the patients also received carboplatin and either paclitaxel for the first 4 cycles.	[n = 278]	5-year OS rates:18.4%	/	SqNSCLC	NCT02775435	Completed	^779,780^
	Chemotherapy	saline placebo for up to 35 cycles; all the patients also received carboplatin and either paclitaxel for the first 4 cycles.	[n = 281]	5-year OS rates:9.7%	/				
III	**Pembrolizumab (PD-1) + Chemotherapy**	pembrolizumab 200 mg intravenously every 3 weeks for up to 35 cycles. All participants received investigator’s choice of fluorouracil (intravenous, 800 mg/m^2^ per day) administered continuously on days 1-5 of each 3-week cycle plus cisplatin (intravenous, 80 mg/m^2^) administered on day 1 of each 3-week cycle or capecitabine (oral, 1000 mg/m^2^) administered twice daily on days 1-14 of each 3-week cycle plus oxaliplatin (intravenous, 130 mg/m^2^) administered on day 1 of each 3-week cycle.	[n = 790]	PD-L1 score ≥1 population:13.0 m;PD-L1 score ≥10 population:15.7 m	/	HER2-negative advanced gastric cancer	NCT03675737	Active, not recruiting	^790^
	Chemotherapy	placebo 200 mg, administered intravenously every 3 weeks for up to 35 cycles. All participants received investigator’s choice of fluorouracil (intravenous, 800 mg/m^2^ per day) administered continuously on days 1-5 of each 3-week cycle plus cisplatin (intravenous, 80 mg/m^2^) administered on day 1 of each 3-week cycle or capecitabine (oral, 1000 mg/m^2^) administered twice daily on days 1-14 of each 3-week cycle plus oxaliplatin (intravenous, 130 mg/m^2^) administered on day 1 of each 3-week cycle.	[n = 789]	PD-L1 score ≥1 population:11.4 m;PD-L1 score ≥10 population:11.8 m	/				
II	**Pembrolizumab (PD-1) + Chemotherapy**	4 cycles of pembrolizumab 200 mg plus carboplatin area under curve 5 mg/mL per min and pemetrexed 500 mg/m^2^ every 3 weeks followed by pembrolizumab for 24 months and indefinite pemetrexed maintenance therapy or to 4 cycles of carboplatin and pemetrexed alone followed by indefinite pemetrexed maintenance therapy	[n = 60]	/	24.5 m	NSCLC	NCT02039674	Completed	^781^
	Chemotherapy	carboplatin area under curve 5 mg/mL per min and pemetrexed 500 mg/m^2^ every 3 weeks followed by pembrolizumab for 24 months and indefinite pemetrexed maintenance therapy or to 4 cycles of carboplatin and pemetrexed alone followed by indefinite pemetrexed maintenance therapy	[n = 63]	/	9.9 m				
III	**Pembrolizumab (PD-1) + Neoadjuvant chemotherapy**	neoadjuvant therapy with four cycles of pembrolizumab (at a dose of 200 mg) every 3 weeks plus paclitaxel and carboplatin	[n = 784]	/	/	TNBC	NCT03036488	Active, not recruiting	^787^
	Neoadjuvant Chemotherapy	placebo every 3 weeks plus paclitaxel and carboplatin	[n = 390]	/	/				
III	**Camrelizumab (PD-1) + Chemotherapy**	camrelizumab (200 mg), combined with up to 6 cycles of paclitaxel (175 mg/m^2^) and cisplatin (75 mg/m^2^).	[n = 298]	15.3 m	6.9 m	ESCC	NCT03691090	Completed	^791^
	Chemotherapy	placebo combined with up to 6 cycles of paclitaxel (175 mg/m^2^) and cisplatin (75 mg/m^2^).	[n = 298]	12.0 m	5.6 m				
II	**Cemiplimab (PD-1) + Chemotherapy**	cemiplimab 350 mg every 3 weeks in combination with four cycles of chemotherapy	[n = 312]	21.1 m	8.2 m	Advanced NSCLC without EGFR, ALK, or ROS1 aberrations	NCT03409614	Active, not recruiting	^782,783^
	Chemotherapy	placebo every 3 weeks in combination with four cycles of chemotherapy	[n = 154]	12.9 m	5.5 m				
III	**Nivolumab (PD-1) + Chemotherapy**	nivolumab (360 mg every 3 weeks or 240 mg every 2 weeks) plus chemotherapy (capecitabine and oxaliplatin every 3 weeks or leucovorin, fluorouracil, and oxaliplatin every 2 weeks)	[n = 789]	36-month OS rates:21%	36-month PFS rates:13%	G/GEJ cancer	NCT02872116	Active, not recruiting	^773,774^
	Chemotherapy	chemotherapy (capecitabine and oxaliplatin every 3 weeks or leucovorin, fluorouracil, and oxaliplatin every 2 weeks)	[n = 792]	36-month OS rates:10%	36-month PFS rates:8%				
III	**Serplulimab (PD-1) + Chemotherapy**	serplulimab (4.5 mg/kg) both in combination with nab-paclitaxel and carboplatin, intravenously in 3-week cycles	[358]	8.3 m	22.7 m	NSCLC	NCT04033354	Active, not recruiting	^784^
	Serplulimab (PD-1)	placebo both in combination with nab-paclitaxel and carboplatin, intravenously in 3-week cycles	[179]	5.7 m	18.2 m				
II	**Toripalimab (PD-1) + Chemotherapy**	three preoperative and five postoperative 3-week cycles of SOX/XELOX, followed by toripalimab monotherapy for up to 6 months	[n = 54]	/	/	G/GEJ cancer	NCT04250948	Active, not recruiting	^775^
	Chemotherapy	three preoperative and five postoperative 3-week cycles of SOX/XELOX	[n = 54]	/	/				
III	**Tislelizumab + Chemotherapy**	tislelizumab (200 mg) intravenously every 3 weeks on day 1, together with an investigator chosen chemotherapy doublet	[n = 326]	17.2 m	/	OSCC	NCT03783442	Active, not recruiting	^792^
	Chemotherapy	placebo intravenously every 3 weeks on day 1, together with an investigator chosen chemotherapy doublet	[n = 323]	13.6 m	/				
Ib	**Tislelizumab (PD-1) + Sitravatinib**	sitravatinib (120 mg) orally one time per day plus tislelizumab 200 mg intravenously every 3 weeks	[n = 25]	/	6.7 m	Melanoma	NCT03666143	Completed	^769^
III	**Atezolizumab (PD-L1) + Nab-paclitaxel**	atezolizumab at a dose of 840 mg, administered intravenously, on days 1 and 15 and received nab-paclitaxel at a dose of 100 mg per square meter of body-surface area, administered intravenously, on days 1, 8, and 15 of every 28-day cycle	[n = 451]	25 m	7.2 m	TNBC	NCT02425891	Completed	^788,789^
	Nab-paclitaxel	placebo, administered intravenously, on days 1 and 15 and received nab-paclitaxel at a dose of 100 mg per square meter of body-surface area, administered intravenously, on days 1, 8, and 15 of every 28-day cycle	[n = 451]	18 m	5.5 m				
III	**Ipilimumab (CTLA-4) + Dacarbazine**	ipilimumab (10 mg per kilogram) plus dacarbazine (850 mg per square meter of body-surface area), given at weeks 1, 4, 7, and 10, followed by dacarbazine alone every 3 weeks through week 22	[n = 250]	11.2 m	/	Melanoma	NCT00324155	Completed	^770^
	Dacarbazine	dacarbazine (850 mg per square meter) plus placebo, given at weeks 1, 4, 7, and 10, followed by dacarbazine alone every 3 weeks through week 22	[n = 252]	9.1 m	/				

When it comes to safety concerns, there is little overlap observed between the common AEs associated with chemotherapy and those linked to immunotherapy. AEs induced by chemotherapy typically encompass myelosuppression, nausea, vomiting, mucositis, alopecia, and neuropathy, whereas immunotherapy is generally well-tolerated. Numerous clinical trials have confirmed that the concurrent use of immunotherapy and chemotherapy produces satisfactory safety profiles and highly effective outcomes. Only a fraction of patients derive benefits from immune-based chemotherapy, and currently, there is a lack of clarity on how to determine which subset of the population will exhibit a sensitive response to the combined treatment. Numerous factors influence how chemotherapy impacts the immune system, including the specific chemotherapy drugs used, their dosages, treatment schedules (whether synchronous or sequential), tumor burden, genomic mutations, and the levels of checkpoint inhibitors expression. A multitude of clinical investigations are presently focusing on this issue, such as the INSIGNA trial (NCT03793179) and the KEYNOTE-975 trial (NCT04210115).

#### Cancer vaccines

The limited immunogenicity of tumor cells plays a vital role in immune evasion, prompting researchers to underscore the significance of therapeutic cancer vaccines that can activate patients’ immune systems to elicit specific immune responses against antigens. Currently, various cancer vaccines are under evaluation in both preclinical and clinical studies, such as Tedopi for lung cancer, ilixadencel for renal cell carcinoma (RCC), GVAX for pancreatic cancer, and PolyPEPI1018 for colorectal cancer (CRC).^795,796^ Among these, sipuleucel-T stands out as the sole approved option for cancer treatment. This innovative therapy involves autologous dendritic cell preparation targeting prostatic acid phosphatase (PAP) and is utilized in managing patients with castration-resistant prostate cancer, showing significant improvements in overall survival outcomes.^797^ Tumor-associated antigens (TAAs), which are non-mutated self-antigens, have exhibited limited clinical efficacy and potential adverse effects like central tolerance and excessive autoimmune toxicity. In contrast, neoantigens, stemming from somatic cell mutations and unique to tumor cells, are absent in normal cells, making them tumor-specific antigens (TSAs). Targeting TSAs in cancer therapy has gained considerable attention and demonstrated substantial advancements.^795^

Furthermore, a substantial upregulation of PD-1 has been observed in antigen-targeted T cells during the utilization of cancer vaccines for treating various cancer types.^798,799^ The primary impact of cancer vaccines primarily influences the initial three stages of the immune response against cancer, involving the release and presentation of tumor-specific antigens to stimulate T cells. Continuous activation of effector T cells within the immunologically dynamic tumor microenvironment (TME) is crucial for subsequent processes and achieving ultimate outcomes. Therefore, combining cancer vaccines with ICB shows promise in improving clinical results. Multiple phase I trials have indicated a synergistic effect of combining cancer vaccine treatment with ICB (Table 4**)**.^800–802^ In a phase I trial named KEYNOTE-603, promising clinical responses were observed when mRNA-4157, a neoantigen, was paired with pembrolizumab, leading to specific CD8^+^ T cell responses targeting neoantigens (NCT03897881).^803^ However, several clinical trials have reported instances where patients did not exhibit the expected response to treatment, failing to demonstrate enhancements in both overall survival (OS) and progression-free survival (PFS).^804–806^ As a result, conflicting conclusions have arisen, necessitating further research studies to investigate the efficacy of cancer vaccine therapy in conjunction with ICB and biomarkers, while determining optimal dosages and treatment regimens.

#### Oncolytic virus therapy

Oncolytic viruses are derived either from natural viruses or genetically engineered viruses, primarily exerting anti-tumor effects through two mechanisms. Initially, these viruses infect tumor cells, replicate within them, and target them for destruction instead of normal cells. This procedure initiates immunogenic cell demise, causing the subsequent liberation of soluble antigens specific to tumors. The thymidine kinase-negative mutant of herpes simplex virus-1 (dlsptk), which exhibits reduced neurotoxicity, has been investigated as a potential therapeutic approach for glioblastoma. Engineered viruses like dlsptk hold promise as novel anti-cancer agents and warrant further evaluation.^807^ Additionally, oncolytic viruses, equipped with non-cytotoxic carriers, have the capacity to encode therapeutic genes like pro-inflammatory cytokines, thus eliciting anti-cancer responses. Talimogene laherparepvec, commonly referred to as T-VEC, is an engineered iteration of herpes simplex virus type 1 (HSV-1) that has been genetically altered to generate granulocyte-macrophage colony-stimulating factor (GM-CSF). This groundbreaking immunotherapy exploits oncolytic viruses in cancer therapy.^808^ Multiple studies suggest that oncolytic viruses boost the release of tumor-specific antigens and facilitate the infiltration of T cells. The collaboration with ICB plays a crucial role in stimulating various stages of the cancer immune response cycle. Furthermore, viral infection can upregulate the expression of immune checkpoint molecules like CTLA-4 and PD-L1, potentially enhancing tumor responsiveness to ICB. Consequently, the concurrent use of oncolytic viruses and ICB has generated significant interest **(**Table 4**)**. A recent randomized phase II trial involving 198 participants revealed a notable enhancement in the objective response rate (ORR) by 39% through the combined administration of T-VEC and ipilimumab in the treatment of advanced unresectable melanoma. This combination outperformed the use of ipilimumab alone, without any additional safety concerns. The findings underscore the potential benefits of combining ICB with viral therapy in the treatment of cancer (NCT01740297).^809,810^

#### Cytokines

The traditional interleukin-2 (IL-2) was previously the most commonly used cytokine, but it has now been superseded by BEMPEG, a novel stimulator of the IL-2 pathway that preferentially targets CD122. BEMPEG activates and boosts the expansion of NK cells and cytotoxic T cells by interacting with IL-2βγ receptors located on the cell membrane. This interaction plays a vital role in promoting immune responses against tumors.^811^ In individuals suffering from advanced solid tumors, the utilization of BEMPEG therapy has shown positive clinical outcomes and controllable safety profile. This is particularly noteworthy in light of the documented increase in PD-L1 expression. These results provide a basis for exploring combined therapeutic approaches incorporating ICB.^812^ The combination of IL-2 and PD-L1 blockade has shown to boost the stimulation of CD8^+^ T cells in a mouse model of persistent inflammation, providing a basis for possible clinical trials.^813^ Positive clinical outcomes in solid tumors have been observed with the combined administration of BEMPEG and nivolumab **(**Table 4**)**. In 2019, the FDA granted Breakthrough Therapy status to the combined therapy of BEMPEG and nivolumab for patients diagnosed with unresectable or metastatic melanoma, following promising preliminary findings from the Phase II PIVOT-02 trial(NCT02983045).^814^ Recent data from the latest PIVOT-02 trial indicate encouraging anti-cancer outcomes and well-tolerated safety profiles in patients with advanced melanoma and renal cell carcinoma (RCC) as their first-line treatment. The combination therapy achieved impressive overall response rates (ORR) of 52.6% and 34.7%, correspondingly (NCT03635983).^815^

#### Co-stimulatory receptor agonists

Various co-stimulatory receptors, including 4-1BB (CD137), OX40 (CD134), and GITR (CD357), are known to be involved in the immune response against tumors. Studies have indicated that activating these receptors with agonists has led to tumor shrinkage in both preclinical and clinical settings.^816–818^ Due to the potential of co-stimulatory agonists to enhance PD-L1 expression while lacking sufficient activation of effector T cells when used alone, it is essential to further assess their efficacy in combination with ICB.^819,820^ Preclinical studies have demonstrated significant synergistic effects in various cancers when combining co-stimulatory agonists with ICB (Table 4).^821–823^ An initial phase Ib trial demonstrated that the concurrent administration of utomilumab, a 4-1BB agonist, and pembrolizumab (anti-PD-1) resulted in a 26.1% combined rate of complete or partial responses among individuals diagnosed with advanced solid tumors. Importantly, this response was achieved without encountering dose-limiting toxicities, underscoring the potential for further exploration of this treatment approach (NCT02179918).^824^ In a Phase Ib trial conducted on patients with metastatic pancreatic cancer, the CD40 agonistic monoclonal antibody sotigalimab demonstrated favorable tolerability and efficacy when administered alongside a combination of gemcitabine, nab-paclitaxel, and nivolumab. This highlights the promising potential of sotigalimab as a treatment option for this challenging disease (NCT03214250).^825^ In 2021, the FDA awarded orphan drug designation to sotigalimab for esophageal cancer, gastroesophageal junction cancer, and pancreatic cancer, citing promising clinical evidence as the basis.

#### CAR-T cells

CAR-T cells refer to T cells that have undergone genetic modification via retroviral or lentiviral transduction, enabling them to exhibit a customized chimeric antigen receptor (CAR) protein. The CAR-T cells are armed with a single-chain variable fragment (scFv) domain to recognize antigens, a T cell activation domain derived from CD3, and a co-stimulatory domain (such as CD28, 4-1BB, or a blend of the two).^826^ Chimeric antigen receptors (CARs), which have been thoroughly researched in hematologic cancers, demonstrate proficiency in recognizing particular cell surface antigens and initiating T cell activation without relying on the MHC system.^827^ While CAR-T cells have the capacity to penetrate tumors and eliminate tumor cells, their activity may be hindered by the immunosuppressive tumor microenvironment caused by elevated immune checkpoints. This suppression can be counteracted through the use of ICB. Therefore, the synergy between ICB and CAR-T cell therapy may result in enhanced anti-tumor immunity, as evidenced by preclinical studies that have prompted additional clinical research on combination therapy.^828,829^ In a 2017 case study, a patient suffering from refractory diffuse large B-cell lymphoma was detailed. Following the ineffectiveness of anti-CD19 CAR-T cell therapy, the patient received pembrolizumab treatment. Upon administration of PD-1 blockade, significant regression of numerous lesions was observed in the patient. Additionally, there was an increase in CART19 cell population and TCRβ T cell clonotypes.^830^

#### Combination immunotherapy with targeted therapy

Targeted therapy involves the precise identification and attack of specific cancer cells using drugs. This approach can quickly reduce tumor size without harming healthy cells by targeting oncogenic driver genes in different types of cancers. Although targeted therapies often produce quick and robust responses, the duration of these responses is typically short-lived, and drug resistance commonly develops, casting doubt on the long-term effectiveness. In contrast, immunotherapy is only effective for a specific group of cancer patients. Although it has a gradual onset of effects, it can trigger long-lasting immune responses with manageable toxicity. This provides enduring immune defense against tumors. Significantly, targeted therapy impacts the cancer immune cycle, notably the initial two stages, upon which the subsequent phases depend on immunotherapy.^831^ Targeted therapy has shown the capacity to trigger immunogenic cell death in cancer cells, enhance the presentation of tumor antigens, and incite the immune system to combat cancer. This effect is evident with inhibitors directed towards cyclin-dependent kinases 4 and 6 (CDK4/6) as well as phosphoinositide 3-kinase (PI3K).^831,832^ Metadherin (MTDH) is frequently overexpressed in aggressive breast cancer cases where the MTDH-SND1 complex decreases the presentation of tumor antigens, hindering T cell infiltration and activation. Disruption of the MTDH-SND1 complex with the small molecule compound C26-A6 exhibited enhanced immune surveillance and sensitivity to PD-1 treatment in a preclinical model of metastatic breast cancer, presenting a promising approach to boost the response to ICB therapy in metastatic breast cancer.^833^ The collaboration between immunotherapy and targeted therapy can mutually reinforce each other, resulting in rapid and sustained inhibition of tumors. Hence, considerable advancements have been achieved in the clinical management of melanoma by adopting this dual strategy (Table 7). Approximately 50% of melanomas harbor BRAF mutations that trigger the MAPK pathway, driving tumor progression. Vemurafenib and dabrafenib, two BRAF inhibitors, exhibit rapid and substantial efficacy in shrinking tumors in melanoma sufferers. These medications have received FDA approval for the treatment of BRAF-mutated melanoma.^834,835^ Research has demonstrated that medications aimed at BRAF/MEK in melanoma have the ability to boost the presentation of tumor antigens, elevate the accumulation and efficacy of effector T cells, and generate pro-inflammatory cytokines. This leads to a fascinating enhancement of anti-tumor immunity.^836^ In certain contexts, BRAF inhibitors have been found to demonstrate immunosuppressive effects. Patients with melanoma who have received prior BRAF inhibitor therapy, along with melanoma cell lines resistant to BRAF inhibitors, have exhibited increased expression of PD-1 and PD-L1. These results hint at the potential benefits of combining targeted therapy with immunotherapy for enhanced treatment outcomes, igniting widespread enthusiasm for immunologically driven combination approaches. The Phase III IMspire150 study recently reported promising results on the safety and effectiveness of combining immunotherapy and targeted therapy for the treatment of metastatic melanoma with BRAF V600E/K mutations.^837^ The research findings revealed that the amalgamation of atezolizumab with targeted therapy involving vemurafenib and cobimetinib (an MEK inhibitor) exhibited superior progression-free survival (PFS) outcomes in contrast to the use of targeted therapy in isolation, yielding a duration of 15.1 months compared to 10.6 months. Furthermore, the combined treatment showcased tolerable safety profiles (NCT02908672).^838^ A recent initial analysis of the Phase II COLET trial indicated that the concurrent use of cobimetinib, atezolizumab, and paclitaxel did not result in an improvement in the objective response rate (ORR) for patients with advanced triple-negative breast cancer (TNBC) (NCT02322814).^839^ In a recent Phase III HR-NBL1/SIOPEN study, it was found that the inclusion of IL-2 in adjuvant therapy did not lead to a significant enhancement in the 3-year event-free survival rate, with rates standing at 56% compared to 60%. Moreover, the study indicated that the co-administration of IL-2 with dinutuximab beta (an anti-GD2 agent) in the treatment of neuroblastoma was associated with heightened combined adverse reactions. These findings have sparked debates regarding the efficacy of combination therapy in various malignancies (NCT01704716).^840^ The toxicity and side effects of combining drugs pose a significant challenge in immune-based targeted therapy, particularly when administered concurrently. Consequently, there have been numerous studies aimed at assessing the safety and efficacy of concurrent or sequential treatment regimens, aiming to leverage the synergistic benefits across different types of cancers.

**Table 7:** Clinical trials of immunotherapy combined with target therapy

Phase	ICIs Combination Therapy (immune checkpoint)	Doses	[n.treatment]	OS	PFS	Disease	Trial	Status	Ref.
I/II	**Pembrolizumab (PD-1) + Enfortumab vedotin (Nectin-4)**	vedotin (1.25 mg/kg) once daily on days 1 and 8 and pembrolizumab (200 mg) (day 1) intravenously once daily in 3-week cycles.	[n = 45]	/	12.3 m	Urothelial cancer	NCT03288545	Active, not recruiting	^848^
I/II	**Pembrolizumab (PD-1) + Niraparib (PARP1/2)**	niraparib (200) mg of oral once daily and pembrolizumab (200 mg) of intravenous on day 1 of each 21-day cycle.	[n = 62]	/	/	Ovarian carcinoma	NCT02657889	Completed	^843^
III	**Atezolizumab (PD-L1) + Vemurafenib (B-RAF) + Cobimetinib(MEK1)**	atezolizumab (840 mg) intravenous day 1 and 15, twicedaily vemurafenib (720 mg), and once-daily cobimetinib (60 mg) 21 days on–7 days off.	[n = 256]	/	39 m	Melanoma	NCT02908672	Active, not recruiting	^835,836^
	Vemurafenib (B-RAF) + Cobimetinib (MEK1)	placebo intravenous day 1 and 15, twicedaily vemurafenib (720 mg), and once-daily cobimetinib (60 mg) 21 days on–7 days off.	[n = 258]	/	25.8 m				
II	**Atezolizumab (PD-L1) + Cobimetinib (MEK1) + Chemotherapy**	Cobimetinib (60 mg QD) on days 1–21 of each 28-day cycle; paclitaxel (80 mg/m2 QW); nab-paclitaxel (100 mg/m2 QW); atezolizumab (840 mg Q2W).	[n = 32]	11.0 m	3.8 m	TNBC	NCT02322814	Terminated	^837^
	Cobimetinib (MEK1) + Chemotherapy	Cobimetinib (60 mg QD) on days 1–21 of each 28-day cycle; paclitaxel (80 mg/m2 QW); nab-paclitaxel (100 mg/m2 QW).	[n = 47]	16. m	5.4 m				
I/II	**Durvalumab (PD-L1) + Olaparib (PARP1/2)**	olaparib (300 mg) in tablet form orally twice daily for 4 weeks and thereafter a combination of olaparib (300 mg) twice daily and durvalumab (1.5 g) via intravenous infusion every 4 weeks until disease progression	[n = 34]	21.5 m	8.2 m	Breast cancer	NCT02734004	Active, not recruiting	^841^
II	**Durvalumab (PD-L1) + Olaparib (PARP1/2)**	Cediranib was taken intermittently 5 days on/2 days off at (15 or 20 mg) (dose levels 1 and 2, respectively) with durvalumab (1500 mg) IV every 4 weeks, and olaparib tablets (300 mg) twice daily.	[n = 35]	/	3.4 m	Ovarian cancer	NCT02484404	Recruiting	^842^
III	**IL-2 + Dinutuximab beta (GD2)**	dinutuximab beta (20 mg/m2 per day as an 8 h infusion for 5 consecutive days)	[n = 206]	62% (5-year OS rate)	57% (5-year EFS rate)	NB	NCT01704716	Recruiting	^838^
	Dinutuximab beta (GD2)	dinutuximab beta plus subcutaneous IL-2 (6×10^6^ IU/m2 per day on days 1-5 and days 8-12 of each cycle)	[n = 200]	63% (5-year OS rate)	53% (5-year EFS rate)				

*NSCLC* non-small cell lung cancer, *SCLC* small cell lung cancer, *NB* neuroblastoma, *TNBC* triple-negative breast cancer, *AML* acute myeloid leukemia, *OCSC* oral cavity squamous cell carcinoma, *CRC* colorectal cancer, *ESCC* esophageal squamous cell carcinoma, *MPM* malignant pleural mesothelioma, *HNSCC* head and neck squamous cell carcinoma, *RCC* renal cell carcinoma, *HCC* hepatocellular carcinoma, *PDAC* pancreatic ductal adenocarcinoma, *mUC* metastatic urothelial carcinoma, *G/GEJ* cancer gastroesophageal junction cancer

Emerging targets for novel drugs have demonstrated significant efficacy beyond traditional targeted therapies. Poly (ADP-ribose) polymerases (PARPs) are a group of closely related enzymes that have the ability to catalyze the transfer of ADP-ribose to target proteins. This process assists in the recognition and repair of DNA damage.^841,842^ Crucially, ovarian cancer, breast cancer, pancreatic cancer, and prostate cancer exhibit lineage mutations in BRCA1/2, making these tumors uniquely susceptible to PARP inhibitors (PARPi). Clinical approval has been granted for the use of PARP inhibitors like olaparib (NCT02734004^843^, NCT02484404^844^) and niraparib (NCT02657889)^845^ as standalone treatments for cancer. Research has demonstrated the effectiveness of these inhibitors in specifically targeting tumors carrying BRCA1/2 mutations, underscoring their promise as a viable treatment for patients with such cancer types. Furthermore, PARP inhibitors have been found to stimulate the STING pathway, enhancing tumor antigenicity and promoting the infiltration of intratumoral T cells across different cancer types from an immunological standpoint.^181,846^ It has been crucially uncovered that PARP inhibitors can boost the levels of PD-L1 expression in breast cancer cell lines and mouse tumor models. This phenomenon may heighten the vulnerability of tumor cells to ICB.^847^ Indeed, through the restoration of anti-tumor immunity, anti-PD-L1 therapy enhances the effectiveness of PARP inhibitors. This serves as evidence supporting the use of immune-based PARP inhibitor therapy.^183,847^ A clinical trial named MEDIOLA, encompassing Phase I and Phase II stages, has demonstrated encouraging anti-cancer effects in individuals with metastatic breast cancer harboring BRCA gene mutations. This was achieved through the concurrent administration of olaparib and durvalumab.^848^ The research demonstrated a remarkable 80% success rate in disease management after 12 weeks of utilizing a combination therapy, with no additional safety issues identified. Furthermore, there is ongoing research and development into antibody-drug conjugates (ADCs). These ADCs are comprised of humanized monoclonal antibodies (mAbs) that are chemically conjugated to cytotoxic payloads. They are designed to selectively recognize and target tumor antigens.^849^ Enfortumab vedotin, an anti-Nectin-4 monoclonal antibody conjugated with monomethyl auristatin E (MMAE), serves as a prime example of targeted therapy against solid tumors with elevated Nectin-4 expression. Preclinical and clinical studies have both highlighted the substantial therapeutic efficacy of this approach in treating urothelial carcinoma (NCT03288545).^850^ Further assessment of immune-based ADC therapy is ongoing.

## Based on nanoparticles: transforming “cold” tumors into “hot” tumors

Immunotherapy has demonstrated effectiveness in only a subset of patients or specific cancer types, primarily attributable to the intricate immunosuppressive tumor microenvironment in solid tumors and the immune tolerance to single-agent therapy. Additionally, systemic delivery of immunotherapeutic drugs may result in severe autoimmune toxicity. Hence, an immediate requirement exists for a novel drug delivery system featuring precise targeting capabilities to manipulate the tumor microenvironment. This system aims to augment the efficacy of immunotherapy. By exploiting a delivery mechanism based on nanoparticles (NPs), the transport of immunomodulators to immune cells can be enhanced, thereby improving the efficiency of immunotherapy.

### Targeting the innate immune pathways in cancer immunotherapy with nanoparticles

Current cancer immunotherapy strategies primarily focus on targeting and boosting anti-tumor adaptive immune responses. However, recent data indicate that in cancer patients, activating the tolerant innate immune system could potentially offset tumor-induced immunosuppression and modify the tumor microenvironment. This suggests that stimulating innate immune responses as a new approach to immunotherapy may result in improved treatment outcomes. Nanoparticles play a crucial role in enhancing cancer immunotherapy by targeting various innate immune pathways. For instance, a nanoparticle complex named BO-112 consisting of Poly I:C and polyethyleneimine (PEI) induces type I interferon and CD8^+^ T cell infiltration into the tumor microenvironment, demonstrating potent anti-tumor activity.^851^ Studies have shown that local therapy with nanoparticles containing TLR7/8 agonists strongly activates macrophages, promoting proliferation of specific CD8^+^ T cells.^852^ Utilizing pH-responsive poly(lactic-co-glycolic acid) (PLGA) nanoparticles loaded with TLR7/8 agonists has enabled precise intratumoral drug delivery. This strategy has successfully boosted the activation of antigen-specific CD8^+^ T cells and NK cells, leading to enhanced anti-tumor effects in a murine melanoma model.^853^ Nanoparticles carrying TLR7/8 agonists, such as R848, show potential in enhancing antibody-dependent immunotherapy mediated by NK cells, potentially reshaping the tumor microenvironment and enhancing the effectiveness of cancer immunotherapy.^854^ Nanoparticles co-loaded with two Toll-like receptor (TLR) agonists demonstrate synergistic effects in promoting adaptive immune responses, functioning as a co-delivery system. For instance, polymer nanoparticles carrying both CpG-ODN and Poly(I:C) induce a more robust Th1 immune response when compared to nanoparticles containing only one adjuvant.^855^ Overall, nanoparticles present a promising approach to precisely and efficiently target innate immune pathways, such as NLR signaling, and enhance cancer immunotherapy by delivering antigens, adjuvants, and immune-modulating factors.

NLR functionality, exemplified by NOD1, can facilitate tumor cell apoptosis and contribute to tumor progression in head and neck squamous cell carcinoma.^856^ Targeting NLRs, especially NOD2, in cancer immunotherapy shows potential, and incorporating NOD2 agonists into nanoparticle carriers enhances their immunostimulatory and anti-cancer activities.^857^ Furthermore, the activation of RLRs, such as RIG-I and MDA5, plays a crucial role in antiviral defense mechanisms and may be significant in cancer treatment by promoting immunogenic cell death in tumor cells.^858^ Polymer nanoparticles delivering synthetic RIG-I agonists have shown potential anti-tumor effects, including promoting cell death and modulating the tumor microenvironment.^859^ Designed pH-responsive polymer nanoparticles have enhanced the delivery of RIG-I agonists, demonstrating increased immunogenic cell death, interferon production, and T cell infiltration in the tumor microenvironment.^860^ Research targeting the delivery of dual-function RIG-I agonists to enhance cancer immunotherapy strategies has been conducted.^861^ These strategies aim to improve treatment outcomes against tumors by leveraging immune responses, whether used alone or in combination with checkpoint inhibitors. Promising approaches for pancreatic cancer immunotherapy involve encapsulating dual-function dsRNA in lipid-calcium nanoparticles to induce pro-inflammatory Th1 responses and increase CD8^+^ T cell levels.^862^ The addition of Riboxxim to PLGA microparticles has been shown to enhance tumor-specific CD8^+^ T cell responses by activating TLR3 and RIG-I signaling pathways in both mouse and human dendritic cells.^863^ Another key class of RLRs, MDA5, can activate IRF3 and IFN-β production, leading to MHC-I upregulation and immune responses within the tumor microenvironment. Targeting MDA5 activation by including TLR3/ML8 agonists in nanoparticles has strengthened anti-tumor immunity, prolonged survival in a pancreatic cancer mouse model by increasing CD8^+^ T cell infiltration and immune cell activation.^864^ Additionally, the activation of the cGAS/STING pathway is crucial for cancer immunity and has been investigated as a promising therapeutic target. Various DNA sensors, including cGAS, can detect intracellular DNA, resulting in downstream signaling and interferon production.^862^ Targeting cGAS-STING with nano-delivery systems, such as CDN-Mn2^+^ particles and mesoporous silica nanoparticles, effectively activate anti-tumor immune responses and reshape the TME in mouse models.^865^ The delivery of a biodegradable mesoporous silica nanoparticle carrying STING agonists has significantly enhanced anti-tumor efficacy and promoted immune cell infiltration, thereby inhibiting tumor growth.^866^ Functionalized nanoparticles containing CDG have shown enhanced immune cell infiltration and improved anti-tumor effects in mouse models of breast cancer and melanoma.^867^ Utilizing nanoparticle carriers to target the cGAS/STING pathways offers a comprehensive strategy for modulating both innate and adaptive immune responses. These techniques showcase the capacity of nanotechnology to bolster cancer immunotherapy by directing crucial immune pathways and augmenting immune reactions against tumors. The amalgamation of STING-LNPs with anti-PD-1 treatment has exhibited mutually reinforcing anti-tumor properties in a melanoma experimental setting. A pioneering methodology encompasses encapsulating a STING agonist within the core of iron oxide nanoparticles, which are enveloped by inert gold nanostars.

### Nanoparticles targeting innate immune cells for cancer immunotherapy

**Fig. 8:**
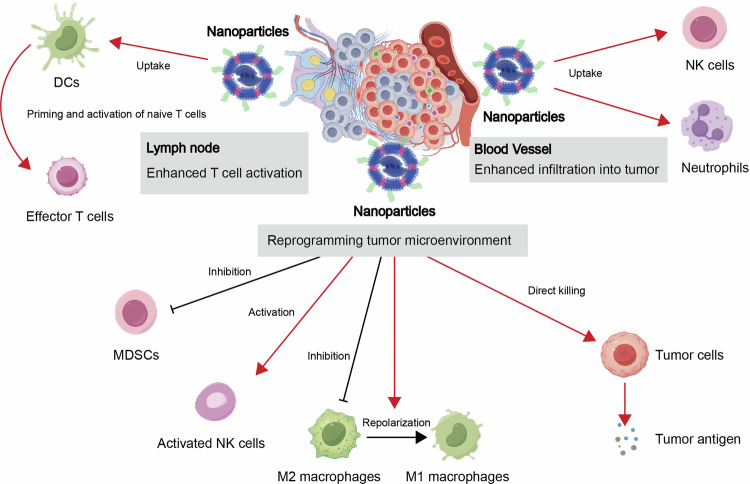
Nanoparticle-Mediated Tumor Microenvironment Intrinsic Immunomodulation Enhancing Cancer Immunotherapy. Engineered nanoparticles administered via subcutaneous or intravenous injection are internalized by either innate immune cells or tumor cells, releasing various payloads in lymph nodes, the tumor immune microenvironment, and vasculature. Nanoparticles possess the capacity to selectively target innate immune pathways, thereby augmenting innate immune responses against cancer. This is primarily due to the stimulation of innate immune cells by released agonists/antigens. This stimulation initiates the secretion of pro-immunogenic cytokines downstream, which subsequently enhances T-cell activation and infiltration in lymph node. Consequently, this process enhances the anti-tumoral immune responses of cytotoxic T lymphocytes (CTLs). Furthermore, nanoparticles effectively modulate the immunosuppressive tumor microenvironment (TME), improving tumors’ sensitivity to immunotherapy by engaging in specific interactions with innate immune cells. This includes elevating the presence of neutrophils and natural killer (NK) cells at tumor sites, diminishing the functions of M2 macrophages and myeloid-derived suppressor cells (MDSCs), transforming M2 macrophages into the M1 phenotype, and inciting the activation of NK cells. In this context, red arrows represent promotion, while black bars symbolize inhibition. This figure was created using Figdraw

Moreover, nanotechnology has been explored for its capability to reprogram the TME with the aim of targeting innate immune cells and enhancing cancer immunotherapy. Utilizing nanoparticles to modulate key innate immune cells, such as macrophages, has demonstrated promising potential in enhancing the body’s immune response against tumors. Researchers have investigated methods to directly manipulate innate immune cells such as macrophages, MDSCs, DCs, NK cells, and neutrophils as strategies to improve cancer immunotherapy (Fig. 8). Progress has been made in targeting innate immune cells with nanocarriers, combined with STING activation and immune receptor modulation, aiming to overcome therapy resistance and improve patient outcomes.^868^

The significance of TAMs in cancer immunotherapy underscores their plasticity and the presence of two conflicting subtypes: M1 TAMs activated by LPS and IFN-γ, and M2 TAMs activated by IL-4 and IL-13. The predominance of immunosuppressive M2 TAMs in the tumor microenvironment has been established. Strategies have been developed to regulate TAM functions through nanoparticles, such as reshaping M2 TAMs into M1 anti-tumor macrophages and blocking the CD47-SIRPα pathway to enhance cancer immunotherapy. Promising results have been demonstrated with targeted elimination of TAMs using strategies like dendritic polymer nanoparticles, bisphosphonate calcium nanoparticles, and silicon-coated gold nanoparticles.^869^ Furthermore, targeting signal-regulating monocytes derived macrophages infiltrating tumors with siRNA-loaded lipid or cationic polymer nanoparticles effectively promotes anti-tumor immune responses, such as through the CSF-1/CSF-1R and CCL2/CCR2 pathways. Various nanoparticle-based approaches have been discussed, including glucose-based liposomes, PBAE nanoparticles delivering M1 polarization factor mRNA, and nanoparticles redirecting M2 TAMs towards an anti-tumor M1 phenotype to enhance immunotherapy efficacy.^870^ Delivery of IL-12 through nanoparticles promotes immune modulation in the tumor microenvironment, while vesicles derived from M1 macrophages or bone marrow even help reprogram TAMs into anti-cancer phenotypes.^871^ Additionally, nanoparticles targeting the CD47-SIRPα axis like SNPACALR&aCD47 show enhanced effects in macrophage-mediated cancer immunotherapy by promoting phagocytosis of cancer cells and blocking immune escape signals.^872^ These diverse nanoparticle-based strategies aim to reshape TAMs and enhance cancer immunotherapy by immune modulation and promoting anti-tumor immune responses.

Targeting of immature MDSCs, which promote tumor growth, angiogenesis, and immune suppression, has been achieved through various nanoparticle-based approaches to eliminate or inhibit their functions, thereby enhancing the efficacy of cancer immunotherapy.^873^ Strategies include using synthetic nanoantibodies (SNAbs) to target MDSCs in a triple-negative breast cancer mouse model, PD-1 antibody-conjugated PLGA nanoparticles to reduce circulating and pulmonary MDSCs, and LPH nanoparticles carrying siRNA targeting HMGA1 to reduce MDSCs and increase colon cancer sensitivity to PD-L1 checkpoint inhibition.^874^ Additionally, zinc-doped iron oxide nanoparticles have been employed to convert immunosuppressive MDSCs into pro-inflammatory states, enhancing anti-tumor efficacy when used in conjunction with radiation therapy. Moreover, nanoparticles designed to switch MDSCs into anti-tumor modes increase the production of anti-tumor cytokines, activate T cell activity, and extend mouse lifespan.^875^ Targeting MDSCs with HDL-like nanoparticles in a melanoma metastasis mouse model leads to increased CD8^+^ T cell numbers, reduced regulatory T cell numbers, slowed tumor growth, reduced tumor burden, and extended survival.^876^ Combination therapy delivering anti-cancer drugs via nanoparticles and modulating MDSCs shows promise in improving outcomes of cancer immunotherapy. Nanoparticle-based approaches provide a precise and efficient way to modulate MDSCs in the tumor microenvironment, enhancing anti-tumor immune responses and improving the effectiveness of cancer treatment.

Dendritic cells, known as specialized antigen-presenting cells, possess the capacity to initiate initial T lymphocyte reactions to particular antigens, serving as vital mediators bridging innate and adaptive immune responses. When dendritic cells migrate to the lymph nodes, they process antigens that they have engulfed in order to present them on MHC class I or II molecules to T cells. This process entails the upregulation of MHC I/II molecules and the activation of co-stimulatory molecules. Concurrently, dendritic cells develop the capacity to generate pro-inflammatory cytokines and chemokines that are beneficial for stimulating T cells.^877^ Therefore, the mature state of dendritic cells is crucial for inducing T cell responses. Several strategies have been developed to activate and mobilize dendritic cells, co-delivering tumor antigens and adjuvants to dendritic cells to activate cytotoxic T lymphocytes (CTLs) and generate dendritic cell-based vaccines. Strategies to modulate dendritic cell function using nanoparticles have attracted significant attention. Certain nanoparticles, such as gold nanorods and carbon black nanoparticles, are recognized as adjuvants that can promote dendritic cell maturation and boost humoral and cellular immune responses through synergistic effects.^878–880^

Numerous approaches utilizing nanoparticles have been devised to enhance the immune reactions of NK cells towards cancer. For instance, in an in situ VX2 liver cancer model, monitoring through magnetic resonance imaging (MRI) revealed that locally delivered immunomodulatory nanocomplex microspheres (IMM-MS) via hepatic artery intervention selectively targeted liver tumors, significantly increasing NK cell infiltration at the site of liver tumors.^881^ Trivalent nanoparticles, referred to as a-EGFR/a-CD16/a-4-1BB NPs, have shown promising capabilities in recruiting and triggering NK cells towards attacking tumor cells with elevated levels of EGFR. The stimulation of NK cells facilitates anti-cancer immune responses. Notably, these nanoparticles are capable of transporting the cytotoxic chemotherapy agent paclitaxel, thus improving treatment efficacy.^882^ Systemic delivery of cationic liposomes carrying TUSC2 plasmid DNA in a Kras mutant mouse lung cancer model results in immune effect NK cell infiltration, providing survival benefits.^883^ Furthermore, chitosan particles carrying NKG2D and IL-21 genes have been shown to effectively activate NK and T cells, leading to inhibition of tumor growth and prolonged mice survival.^884^ Exosomes derived from dendritic cells also exhibit potent activation effects on NK cells.^885^ Research has demonstrated that Dex can stimulate the expansion and activation of NK cells in secondary lymphoid organs of mice by activating the interleukin 15 receptor alpha subunit (IL-15Ra) and NKG2D-dependent pathways. Significantly, Dex immunization shows potential in boosting NK cell count and efficacy in advanced-stage melanoma patients.^886^

The focus of the discussion is the role of neutrophils in cancer, highlighting different subtypes such as N1/N2 neutrophils, TANs, and PMN-MDSCs.^391^ The primary research focus lies in investigating neutrophils in animal models or studying the functions of neutrophils in peripheral blood in humans, while information regarding TANs in cancer patients is scarce.^887^ Neutrophils primarily engulf nanoparticles through phagocytosis, thereby enhancing prospects for improving cancer immunotherapy by delivering nanoparticles via neutrophils. Various approaches have been explored, with promising results achieved in inhibiting tumor recurrence with drug-carrying neutrophils, particularly in glioblastoma.^888^ Overall, precise targeting of the tumor immune microenvironment with nanoparticles holds promise in enhancing the effectiveness of cancer immunotherapy.

## Conclusion and perspectives

The impact of “cold” and “hot” tumor phenotypes on cancer immunotherapy has garnered significant attention, as transitioning from a “cold” to a “hot” tumor environment can substantially enhance treatment efficacy. Alterations in the interplay between tumor cells and immune cells have a profound influence on cancer’s evolution and advancement. Highlighting changes in the tumor immune microenvironment and immune functionality is crucial for enhancing treatment efficacy and improving survival rates among advanced-stage cancer patients. Utilizing various diagnostic approaches, such as molecular biology, cytology, histology, imaging, and artificial intelligence, allows for a comprehensive examination of tumor tissues, blood samples, and relevant clinical data from patients. This thorough analysis assists in identifying unique immune traits specific to individual patients. These methods will be applied innovatively in cancer immunotherapy to convert “cold” tumors into “hot” tumors, creating a favorable environment to enhance the effectiveness of immunotherapy, with significant clinical application potential.

The concept of personalized cancer immunotherapy has gained significant momentum in recent years. One of the primary challenges in achieving this ambitious objective is the incomplete understanding of cancer-immune interaction parameters and the absence of standardized measurement methods for most parameters, even when knowledge is available. Precise evaluation of these factors is crucial, as their significance varies among patients. Therefore, recognizing critical characteristics, whether immune system-related or tumor-specific, at the initial diagnosis stage is vital for developing effective classification methods that can guide subsequent therapeutic strategies. Various treatment approaches have been suggested to target or devise strategies for “hot,” “change,” and “cold” tumors. It is reasonable to anticipate a need for additional intervention methods for “cold” tumors. Importantly, all proposed treatment strategies ultimately incorporate immunotherapy to maximize efficacy. Given the pivotal role of T cells in fighting cancer, a comprehensive assessment of current T cell profiles and the immune microenvironment should be a standard practice to differentiate between individual cases, both in clinical settings and during early research phases. This data will facilitate translating research discoveries into practical clinical applications.

The increasing comprehension of tumor microenvironment (TME) mechanisms and therapeutic strategies has resulted in a significant rise in clinical trial opportunities within the realm of cancer immunology. Consequently, researchers and clinicians are presented with a multitude of potential intervention avenues aimed at developing integrated approaches that target intrinsic immune resistance in “cold” tumors. With the continual expansion of approved therapeutic protocols and the growing significance of molecular and immune phenotypes in diverse tumors, personalized treatment options are expected to become more widespread in clinical settings. A critical challenge lies in prioritizing treatment interventions and effectively combining a range of possible methods. Unfortunately, the lack of a comprehensive understanding of cancer-immune interactions leads combination therapy and dosage regimens to be mainly empirical or driven by pragmatism. Utilizing high-resolution molecular analysis, which includes single-cell genomics, multidimensional cellular phenotyping, and comprehensive topological assessment of tumor biopsies, enables the identification of diverse immune cell populations, as well as the molecular and functional statuses of immune cells within the TME, and their dynamic responses to treatments. Recognizing the pivotal role of immune cells in their dynamic interplay with tumor cells during immunotherapy is paramount. This understanding aids in formulating crucial pathway models that elucidate the correlation between cellular and tumor tissue states and the responses to specific interventions, thereby facilitating the design of rational combination strategies. Moreover, it emphasizes the significance of systemic benchmarks and real-time biopsies throughout the treatment process to capture the dynamic shifts in cellular composition, functional statuses, and the localization of cellular populations.

Timing plays a critical role in the efficacy of immune checkpoint inhibitors. The features of “cold” tumors, such as limited lymphocyte infiltration and a prevalence of immunosuppressive cells, present significant obstacles to immunotherapy. Resistance to immune checkpoint inhibitors, whether primary or acquired, remains a major limiting factor. Both host-related intrinsic and extrinsic factors, including environmental exposures, contribute to this resistance and necessitate a deeper understanding. Immune-related adverse events pose a complex challenge in immunotherapy. Effective management of these events is vital for patient safety, and ongoing research strives to develop more precise treatment strategies. In the realm of “cold” tumor treatment, the future appears promising for immunomodulators, opening up various innovative pathways. Personalized immunotherapy approaches—such as Precision Medicine, which focuses on tailoring treatment plans to individual patients based on their unique genetic and environmental traits—are gaining momentum. Predictive factors like CD8^+^ T cell concentration, tumor mutation burden, and PD-1 expression play a crucial role in guiding the customization of treatments. Neoantigens, tumor-specific mutated proteins, are promising targets for personalized cancer vaccines. Developing neoantigen vaccines tailored to each patient using mRNA technology and LNPs shows significant potential. Combination therapy—leveraging the synergistic effects of immune checkpoint blockade with immune cell therapies and novel strategies like cytokine-based bifunctional molecules—holds great promise in boosting treatment effectiveness, particularly in challenging “cold” tumor environments.
